# New Conjugates of Polyhydroxysteroids with Long-Chain Fatty Acids from the Deep-Water Far Eastern Starfish *Ceramaster patagonicus* and Their Anticancer Activity

**DOI:** 10.3390/md18050260

**Published:** 2020-05-15

**Authors:** Timofey V. Malyarenko, Alla A. Kicha, Olesya S. Malyarenko, Viktor M. Zakharenko, Ivan P. Kotlyarov, Anatoly I. Kalinovsky, Roman S. Popov, Vasily I. Svetashev, Natalia V. Ivanchina

**Affiliations:** 1 G.B. Elyakov Pacific Institute of Bioorganic Chemistry, Far Eastern Branch of the Russian Academy of Sciences, Pr. 100-let Vladivostoku 159, Vladivostok 690022, Russia; kicha@piboc.dvo.ru (A.A.K.); malyarenko.os@gmail.com (O.S.M.); kaaniw@piboc.dvo.ru (A.I.K.); prs_90@mail.ru (R.S.P.); ivanchina@piboc.dvo.ru (N.V.I.); 2 Department of Bioorganic Chemistry and Biotechnology, School of Natural Sciences, Far Eastern Federal University, Sukhanova Str. 8, Vladivostok 690000, Russia; rarf247@gmail.com (V.M.Z.); ivan_1999_19@icloud.com (I.P.K.); 3 A.V. Zhirmunsky National Scientific Center of Marine Biology, Far Eastern Branch of the Russian Academy of Sciences, 17 Palchevsky St., Vladivostok 690041, Russia; vsvetashev@mail.ru

**Keywords:** polyhydroxysteroidal esters, NMR spectra, fatty acids, starfish, *Ceramaster patagonicus*, cytostatic activity, soft agar assay, wound healing assay

## Abstract

Four new conjugates, esters of polyhydroxysteroids with long-chain fatty acids (**1**–**4**), were isolated from the deep-water Far Eastern starfish *Ceramaster patagonicus.* The structures of **1**–**4** were established by NMR and ESIMS techniques as well as chemical transformations. Unusual compounds **1**–**4** contain the same 5α-cholestane-3β,6β,15α,16β,26-pentahydroxysteroidal moiety and differ from each other in the fatty acid units: 5′*Z*,11′*Z*-octadecadienoic (**1**), 11′*Z*-octadecenoic (**2**), 5′*Z*,11′*Z*-eicosadienoic (**3**), and 7′*Z*-eicosenoic (**4**) acids. Previously, only one such steroid conjugate with a fatty acid was known from starfish. After 72 h of cell incubation, using MTS assay it was found that the concentrations of compounds **1**, **2**, and **3** that caused 50% inhibition of growth (IC_50_) of JB6 Cl41 cells were 81, 40, and 79 µM, respectively; for MDA-MB-231 cells, IC_50_ of compounds **1**, **2**, and **3** were 74, 33, and 73 µM, respectively; for HCT 116 cells, IC_50_ of compounds **1**, **2**, and **3** were 73, 31, and 71 µM, respectively. Compound **4** was non-toxic against tested cell lines even in three days of treatment. Compound **2** (20 µM) suppressed colony formation and migration of MDA-MB-231 and HCT 116 cells.

## 1. Introduction

Starfish (Echinodermata, Asteroidea) are a rich source of various secondary metabolites: peptides, fatty acids, polar steroids and their glycosides, carotenoids, quinone pigments, and also sphingolipids and their derivatives [1]. Polar steroid compounds are a major class of starfish secondary metabolites. They are polyhydroxysteroids; glycosides of polyhydroxysteroids (mono-, bi-, and triglycosides); cyclic glycosides; and asterosaponins –oligoglycosides containing 3β,6α-dihydroxysteroidal moieties with the a 9(11)-double bond and an *O*-sulfate group at C-3 [2,3,4,5,6,7,8,9]. Generally, polyhydroxysteroids have from four to nine hydroxyl groups: in the steroid nucleus at positions 3β (or more rarely 3α), 6α (or β), 8, 15α (or β), and 16β and, more rarely, at positions 4β, 5, 7α (or β), and 14; in the side chains at positions 26 and 24, or simultaneously in the side chain at 25 and 26 positions. The hydroxyl group can also be attached to C-28 or C-29 of the ergostane or stigmastane skeletons, respectively [2,3,4,5,6,7,8,9]. Starfish polyhydroxysteroids often occur in sulfated forms. Usually the sulfate group is located at C-3 or C-15 of the steroidal nucleus or at C-24 or C-26 of the side chain. Sometimes, starfish polyhydroxysteroids and related glycosides are found in the form of conjugates with amino acids. For example, several rare derivatives of starfish polyhydroxysteroids such as polyhydroxysteroid amides conjugated with taurine were isolated from the Arctic starfish *Asterias microdiscus* [10]. In addition, only one rare steroidal ester of polyhydroxysteroid with long-chain fatty acid, (25*S*)-5α-cholestane-3β,6α,7α,8,15α,16β-hexaol-26-yl 14′*Z*-eicosenoate, was isolated from the starfish *Asterina pectinifera* [11].

Starfish polar steroids have been reported to show a wide spectrum of biological activities, including cytotoxic, antiviral, antibacterial, neuritogenic, and anticancer effects [1,2,3,4,5,6,7,8,9]. Some starfish polyhydroxylated compounds have been shown to exhibit significant cytotoxicity. For example, certonardosterol D_2_ revealed cytotoxic effects with effective doses that inhibited a viability of 50% cells (ED_50_) against human lung cancer A549 (ED_50_ = 0.15 μg/mL), melanoma SK-MEL-2 (ED_50_ = 0.09 μg/mL), ovarian cancer SK-OV-3 (ED_50_ = 0.08 μg/mL), CNS cancer XF498 (ED_50_ = 0.07 μg/mL), and colon cancer HCT15 (ED_50_ = 0.01 μg/mL) cell lines [12]. However, (25*S*)-5α-cholestane-3β,6α,7α,8,15α,16β-hexaol-26-yl 14′*Z*-eicosenoate did not possess inhibitory activity against herpes simplex virus type 1 (HSV-1) and human liver carcinoma HepG2 cells in vitro [11].

Herein, we report the results of the structural elucidation of four new polyhydroxysteroidal esters (**1**–**4**) with fatty acids from the methanol chloroform ethanolic extract of the deep-water Far Eastern starfish *Ceramaster patagonicus* collected at the Sea of Okhotsk near Iturup Island. We examined the cytotoxic and cytostatic activities of **1**–**4** on mouse normal epidermal, human breast cancer, and colorectal carcinoma cells. In addition, the effects of these compounds on the colony formation and migration of human breast cancer and colorectal carcinoma cells were investigated using soft agar and wound healing assays.

## 2. Results and Discussion

### 2.1. The Isolation and Structure Elucidation of Compounds ***1***–***4*** from C. patagonicus

**Figure 1:**
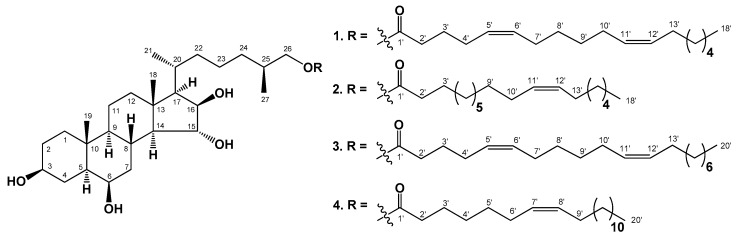
The structures of compounds **1**–**4** isolated from *C. patagonicus.*

The concentrated methanol chloroform ethanolic extract of *C. patagonicus* was partitioned between H_2_O and AcOEt/BuOH, and the organic layer was washed with cold acetone. The acetone-soluble part was subjected to separation by chromatography on silica gel column followed by HPLC on semi-preparative Diasfer-110-C18 column to obtain four new polyhydroxysteroidal compounds: (25*S*)-5α-cholestane-3β,6β,15α,16β-tetraol-26-yl 5′*Z*,11′*Z*-octadecadienoate (**1**), (25*S*)-5α-cholestane-3β,6β,15α,16β-tetraol-26-yl 11′*Z*-octadecenoate (**2**), (25*S*)-5α-cholestane-3β,6β,15α,16β-tetraol-26-yl 5′*Z*,11′*Z*-eicosadienoate (**3**), and (25*S*)-5α-cholestane-3β,6β,15α,16β-tetraol-26-yl 7′*Z*-eicosenoate (**4**) (Figure 1).

**Figure 2:**
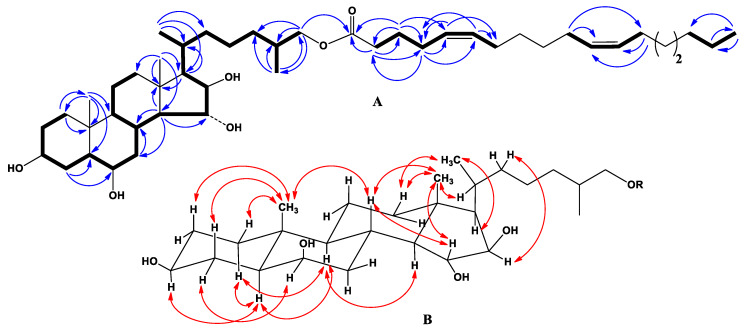
(**A**) ^1^H-^1^H COSY and key HMBC correlations for compound **1**. (**B**) Key ROESY correlations of steroidal moiety for compounds **1**–**4**.

The IR spectrum of compound **1** showed the presence of hydroxyl (3504 cm^−1^), ester carbonyl (1723 cm^−1^), and olefinic (1602 cm^−1^) groups. The molecular formula of compound **1** was determined to be C_45_H_78_O_6_ from the [M + Na]^+^ sodiated adduct ion peak at *m/z* 737.5695 in the (+)HRESIMS, the [(M − H) + AcOH]^−^ ion peak at *m/z* 773.5934, and the [M − H]^−^ deprotonated ion peak at *m/z* 713.5726 in the (–)HRESIMS (Figures S1 and S2). The ^1^H- and ^13^C-NMR spectroscopic data belonging to the tetracyclic moiety of **1** showed the resonances of protons and carbons of two angular methyls, CH_3_-18 and CH_3_-19 (*δ*_H_ 1.27 s, 1.47 s; *δ*_C_ 14.9, 16.2), and four oxygenated methines, HC-3 (*δ*_H_ 3.99 m; *δ*_C_ 71.1), HC-6 (*δ*_H_ 4.15 brs; *δ*_C_ 71.2), HC-15 (*δ*_H_ 4.40 brd (*J* = 10.0); *δ*_C_ 84.7), and HC-16 (*δ*_H_ 4.66 brd (*J* = 7.3); *δ*_C_ 82.2) (Table 1, Figures S4–S12). The NMR spectra of steroidal side chain indicated the existence of two secondary methyls, CH_3_-21 (*δ*_H_ 1.13 d (*J* = 6.8); *δ*_C_ 18.3) and CH_3_-27 (*δ*_H_ 0.94 d (*J* = 6.8); *δ*_C_ 17.0), and one oxygenated methylene, H_2_C-26 (*δ*_H_ 4.11 dd (*J* = 10.6, 5.4), 3.96 dd (*J* = 10.6, 6.8); *δ*_C_ 69.1) (Table 1, Figures S4–S12). The ^1^H-^1^H COSY and HSQC correlations attributable to steroidal moiety revealed the corresponding sequences of protons at C-1 to C-8; C-8 to C-12 through C-9 and C-11; C-8 to C-17 through C-14; C-17 to C-21, and C-20 to the end of the side chain (Figure 2A, Figures S13–S16). The key HMBC cross-peaks, such as H-1/C-10, C-19; H-4/C-5, C-6; H-7/C-8; H-14/C-7, C-8, C-13, C-15, C-18; H-17/C-13, C-20; H_3_-18/C-12, C-13, C-14, C-17; H_3_-19/C-1, C-5, C-9, C-10; H_3_-21/C-17, C-20, C-22; H_2_-26/C-24, C-25, C-27; and H_3_-27/C-24, C-25, C-26 confirmed the overall structure of the steroidal part of **1** (Figure 2A, Figures S17 and S18).

The key ROESY cross-peaks showed the common 5α/9α/10β/13β stereochemistry of the steroidal nucleus, 3β,6β,15α,16β-configurations of oxygenated substituents and 26-hydroxycholestane side chain in **1** (Figure 2B, Figures S19 and S20). The 20*R*-configuration was assumed on the basis of ROESY correlations of H_3_-18/H-20, H_β_-16/H-22, and H_3_-21/H_β_-12 (Figure 2B, Figures S19 and S20). ^1^H- and ^13^C-NMR data of the steroidal part of compound **1** were practically identical to those of (25*S*)-5α-cholestane-3β,6β,15α,16β,26-pentaol, which was isolated for the first time from the starfish *Hacelia attenuata* [13] and later from the starfish *C. patagonicus* [14], which confirmed the identity of the steroid parts of these compounds and indicated the 25*S*-configuration of the side chain of **1**. Based on these data, the steroidal moiety of **1** was determined as (20*R*,25*S*)-5α-cholestane-3β,6β,15α,16β,26-pentaol.

In addition, the ^1^H- and ^13^C-NMR spectra of compound **1** indicated the presence of one primary methyl, CH_3_-18′ (*δ*_H_ 0.88 t (*J* = 6.9); *δ*_C_ 14.0); four olefinic methines, HC-5′ (*δ*_H_ 5.44 m; *δ*_C_ 128.9), HC-6′ (*δ*_H_ 5.51 m; *δ*_C_ 130.9), HC-11′ (*δ*_H_ 5.48 m; *δ*_C_ 129.8), and HC-12′ (*δ*_H_ 5.49 m; *δ*_C_ 130.1); two characteristic allyl methylenes, H_2_C-4′ (*δ*_H_ 2.17 m; *δ*_C_ 26.7) and H_2_C-7′ (*δ*_H_ 2.10 m; *δ*_C_ 27.4); one ester carbonyl (*δ*_C_ 173.3); and one characteristic methylene, H_2_C-2′ (*δ*_H_ 2.43 t (*J* = 7.4); *δ*_C_ 33.7), located at the α-position from ester carbonyl group (Table 2, Figures S4–S12). The (–)ESIMS/MS of the ion [M − H]^−^ at *m/z* 713 contained the fragment ion peaks at *m/z* 449 [C_27_H_45_O_5_]^−^, corresponding to the loss of long-chain fatty acid residue, and at *m*/*z* 279 [C_18_H_31_O_2_]^−^, corresponding to the loss of steroidal moiety. The ^1^H-^1^H COSY and HSQC correlations attributable to fatty acid unit revealed the corresponding sequences of protons at C-2′ to C-7′, C-10′ to C-13′, and C-16′ to C-18′ (Figure 2A, Figures S13–S16), while the key HMBC cross-peaks, such as H-2′/C-1′, C-3′, C-4′; H-3′/C-1′, C-2′, C-4′, C-5′; H-4′/C-2′, C-5′, C-6′; H-5′/C-4′, C-7′; and H-6′/C-4′, C-7′, confirmed the localization of Δ^5(6)^-double bound in the fatty acid unit of **1** (Figure 2A, Figures S17 and S18). The geometry of the double bond in the long-chain fatty acids can be determined on the basis of the ^13^C-NMR chemical shift of the methylene carbon adjacent to the olefinic carbon (*δ*_C_ ≈ 27 for (*Z*) isomers and *δ*_C_ ≈ 32 for (*E*) isomers). ^13^C-NMR spectrum of compound **1** indicated the presence of four characteristic allyl carbons: C-4′ (*δ*_C_ 26.7), C-7′ (*δ*_C_ 27.4), C-10′ (*δ*_C_ 27.4), and C-13′ (*δ*_C_ 27.1). Thus, the olefin groups in **1** were determined to have *cis* (*Z*) geometry. Compound **1** was methanolyzed with methanolic hydrochloric acid to give the fatty acid methyl esters (FAME-1) of **1**. Additionally, 4,4-dimethyloxazoline derivatives (DMOXs-1) of fatty acids of compound **1** were prepared from FAME-1 according to the procedure described previously [15]. This method allows for determining double bond in polyunsaturated fatty acids. Fatty acids were identified by equivalent chain length (ECL) values in GC analysis and mass spectra of FAME-1 and DMOXs-1 derivatives in GC-MS [15,16]. GC-MS analysis showed the existence of one major component, which was characterized as methyl 5′*Z*,11′*Z*-octadecadienoate. Minor components and their percentage are shown in Table 3. On the basis of all the above-mentioned data, the structure of **1** was determined to be (25*S*)-5α-cholestane-3β,6β,15α,16β-tetraol-26-yl 5′*Z*,11′*Z*-octadecadienoate. It should be noted that NMR, MS, TLC, and HPLC analyses indicated the homogeneity of compounds **1**–**4**; however, GLC-MS analysis of methyl ester of fatty acids **1**–**4** showed the presence of other fatty acids.

The IR spectrum of compound **2** showed the presence of hydroxyl (3504 cm^−1^), ester carbonyl (1723 cm^−1^), and olefinic (1602 cm^−1^) groups. The molecular formula of compound **2** was determined to be C_45_H_80_O_6_ from the [M + Na]^+^ sodiated adduct ion peak at *m/z* 739.5852 in the (+)HRESIMS, the [(M − H) + AcOH]^−^ ion peak at *m/z* 775.6096, and the [M − H]^−^ deprotonated ion peak at *m/z* 715.5889 in the (–)HRESIMS (Figures S21 and S22). The comparison of the molecular masses of **1** and **2** showed that the difference between **1** and **2** is 2 atomic mass units (amu). The comparison of ^1^H- and ^13^C-NMR spectra and application of extensive 2D NMR analysis of compounds **1** and **2**–**4** exhibited that steroidal moieties of **2**–**4** are identical to that of compound **1**, while long-chain fatty acid residues of **1**–**4** differ from each other by the length of the hydrocarbon chains and the number and positions of double bonds (Figure 1, Table 1 and Table 2).

The ^1^H- and ^13^C-NMR spectra of fatty acid residue of compound **2** indicated the existence of one primary methyl, CH_3_-18′ (*δ*_H_ 0.87 t (*J* = 6.9); *δ*_C_ 14.0); two olefinic methines, HC-11′ and HC-12′ (2H, each *δ*_H_ 5.49 m; *δ*_C_ 130.0); two characteristic allyl methylenes, H_2_C-10′ (*δ*_H_ 2.11 m; *δ*_C_ 27.3) and H_2_C-13′ (*δ*_H_ 2.11 m; *δ*_C_ 27.1); one ester carbonyl (*δ*_C_ 173.3); and one characteristic methylene, H_2_C-2′ (*δ*_H_ 2.40 t (*J* = 7.5); *δ*_C_ 34.3), located at the α-position from ester carbonyl group (Table 2, Figures S24 and S25). The (–)ESIMS/MS of the ion [M − H]^−^ at *m/z* 715 contained the fragment ion peaks at *m/z* 449 [C_27_H_45_O_5_]^−^, corresponding to the loss of long-chain fatty acid, and *m*/*z* 281 [C_18_H_33_O_2_]^−^, corresponding to the loss of steroidal moiety. Difference of 2 amu between fragment ion peaks at *m*/*z* 281 [C_18_H_33_O_2_]^−^ and 279 [C_18_H_31_O_2_]^−^ in the (–)ESIMS/MS mass spectra of compounds **2** and **1** indicated the absence of one double bond in the fatty acid moiety of compound **2** compared to **1** (Figures S3 and S23). The comparison of ^1^H- and ^13^C-NMR data of **2** and **1** also confirmed this conclusion (Table 2, Figures S4–S12 and S24, S25). The *cis* (*Z*) geometry of the double bond in the long-chain fatty acid moiety of **2** can be determined on the basis of the ^13^C-NMR chemical shift of the C-10′ (*δ*_C_ 27.3) and C-13′ (*δ*_C_ 27.1).

Fatty acid units in **2** were identified by ECL values of fatty acids in GC analysis and mass spectra of FAME-2 and DMOXs-2 derivatives in GC-MS [16] similar to compound **1**. GC-MS analysis showed the existence of one major component, which was identified as methyl 11′*Z*-octadecenoate. Minor components and their percentage are shown in Table 3. Thereby, the structure of **2** was established to be (25*S*)-5α-cholestane-3β,6β,15α,16β-tetraol-26-yl 11′*Z*-octadecenoate.

The IR spectrum of compound **3** showed the presence of hydroxyl (3510 cm^−1^), ester carbonyl (1723 cm^−1^), and olefinic (1603 cm^−1^) groups. The molecular formula of compound **3** was determined to be C_47_H_82_O_6_ from the [M + Na]^+^ sodiated adduct ion peak at *m/z* 765.6008 in the (+)HRESIMS, the [(M − H) + AcOH]^−^ ion peak at *m/z* 801.6254, and the [M − H]^−^ deprotonated ion peak at *m/z* 741.6044 in the (–)HRESIMS (Figures S30 and S31). The comparison of the molecular masses of **1** and **3** showed that the difference between them is 28 amu. At the same time, the ^1^H- and ^13^C-NMR spectra of compounds **1** and **3** were almost identical (Table 1 and Table 2, Figures S4–S12 and S33, S34). The (–)ESIMS/MS of the ion [M − H]^−^ at *m/z* 741 contained the fragment ion peaks at *m*/*z* 449 [C_27_H_45_O_5_]^−^, corresponding to the loss of long-chain fatty acid, and *m*/*z* 307 [C_20_H_35_O_2_]^−^, corresponding to the loss of steroidal moiety. Fatty acid units in **3** were identified by ECL values of fatty acids in GC analysis and mass spectra of FAME-3 and DMOXs-3 derivatives in GC-MS [15,16]. GC-MS analysis showed the existence of one major component, which was identified as methyl 5′*Z*,11′*Z*-eicosadienoate. Accordingly, the structure of **3** was determined to be (25*S*)-5α-cholestane-3β,6β,15α,16β-tetraol-26-yl 5′*Z*,11′*Z*-eicosadienoate. The *cis* (*Z*) geometry of the double bond in the long-chain fatty acid moiety of **3** can be determined on the basis of the ^13^C-NMR chemical shift of the C-10′ (*δ*_C_ 27.3) and C-13′ (*δ*_C_ 27.1).

The IR spectrum of compound **4** showed the presence of hydroxyl (3515 cm^−1^), ester carbonyl (1723 cm^−1^), and olefinic (1602 cm^−1^) groups. The molecular formula of compound **4** was determined to be C_47_H_84_O_6_ from the [M + Na]^+^ sodiated adduct ion peak at *m/z* 767.6157 in the (+)HRESIMS, the [(M − H) + AcOH]^−^ ion peak at *m/z* 803.6409, and the [M − H]^−^ deprotonated ion peak at *m/z* 743.6199 in the (–)HRESIMS (Figures S39 and S40). The comparison of the molecular masses of **3** and **4** showed the difference of 2 amu between **3** and **4**. The (–)ESIMS/MS of the ion [M − H]^−^ at *m/z* 743 contained the fragment ion peaks at *m*/*z* 449 [C_27_H_45_O_5_]^−^, corresponding to the loss of long-chain fatty acid, and *m*/*z* 309 [C_20_H_37_O_2_]^−^, corresponding to the loss of steroidal moiety. Fatty acids were identified in the same manner as for other compounds [15,16]. GC-MS analysis showed the existence of one major component, which was characterized as methyl 7′*Z*-eicosenoat. On the basis of these results, the structure of **4** was determined to be (25*S*)-5α-cholestane-3β,6β,15α,16β-tetraol-26-yl 7′*Z*-eicosenoate.

### 2.2. In Vitro Anticancer Activity of Compounds ***1**–**4***

#### 2.2.1. The Cytotoxic Activity of Compounds **1**–**4** against Normal and Cancer Cells

At the first stage of biological activity evaluation, the cytotoxicity of compounds **1**–**4** against mouse normal epidermal JB6 Cl41 cells, human breast cancer MDA-MB-231 cells, and colorectal carcinoma HCT 116 cells was determined by MTS assay, which is based on the cleavage of (3-(4,5-dimethylthiazol-2-yl)-5-(3-carboxymethoxyphenyl)-2-(4-sulfophenyl)-2H-tetrazolium) (MTS reagent) into a formazan product soluble in tissue culture medium. It was found that compounds **1**–**4** suppressed the cell viability by less than 15% at 100 µM after 24 h of incubation (Figure S48).

**Figure 3:**
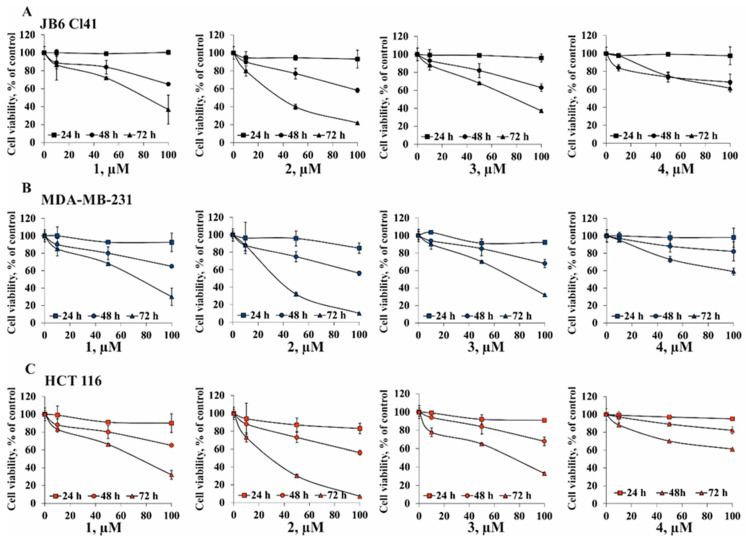
The cytostatic activity of compounds **1**–**4** against normal epidermal JB6 Cl41 cells, breast cancer MDA-MB-231 cells, and colorectal carcinoma HCT 116 cells. (**A**) JB6 Cl41, (**B**) MDA-MB-231, or (**C**) HCT 116 cells were treated with compounds **1**–**4** at concentrations of 1–100 µM for 24, 48, and 72 h. Cell viability was estimated using the MTS assay. Data are represented as the mean ± SD as determined from triplicate experiments.

Many natural compounds do not possess direct cytotoxic activity but are able to suppress cell viability time-dependently [17]. That is why the cytostatic activity of compounds **1**–**4** was determined against JB6 Cl41, MDA-MB-231, and HCT 116 cell lines for 24, 48, and 72 h. After 72 h cell incubation, it was shown that the concentrations of compounds **1**, **2**, and **3** that caused 50% inhibition of growth (IC_50_) of JB6 Cl41 cells were 81, 40, and 79 µM, respectively (Figure 3A); for MDA-MB-231 cells, IC_50_ concentrations of compounds **1**, **2**, and **3** were 74, 33, and 73 µM, respectively; for HCT 116 cells, IC_50_ concentrations of compounds **1**, **2**, and **3** were 73, 31, and 71 µM, respectively (Figure 3). The cytostatic effect of **2** was more prominent in colorectal carcinoma HCT 116 cells.

It should be noted that investigated compounds did not exert a selective effect on cancer cells, because the viability of normal cells was suppressed as well. Therefore we checked the assumption whether compounds **1**–**4** were able to influence the process of carcinogenesis (colony formation, growth, and migration of cancer cells) at the non-toxic concentration of 20 µM.

#### 2.2.2. The Effect of Compounds **1**–**4** on the Colony Formation and Growth of Human Cancer Cells

The formation of colonies is one of the most stringent characteristics for malignant transformation in cells [18]. In the present study the soft agar assay was used to investigate the effect of compounds **1**–**4** on capability of cancer cells to form colonies.

**Figure 4:**
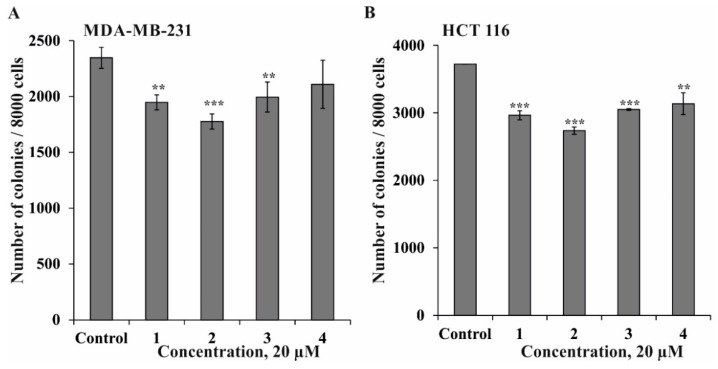
The effect of compounds **1**–**4** on colony formation in human cancer cells. MDA-MB-231 (**A**) or HCT 116 cells (**B**) (2.4 × 10^4^) were treated with or without investigated compounds (20 µM) and applied onto 0.3% Basal Medium Eagle (BME) agar containing 10% FBS, 2 mM L-glutamine, and 25 µg/mL gentamicin. After 14 days of incubation, the number of colonies was evaluated under a microscope with the aid of the ImageJ software program. Results are expressed as the mean ± standard deviation (SD). The asterisks (** *p* < 0.01, *** *p* < 0.001) indicate a significant decrease in colony number of cancer cells treated by compounds compared with control.

It was found that compounds **1**, **2**, **3**, and **4** inhibited colony formation in MDA-MB-231 cells by 17%, 26%, 15%, and 10%, respectively. Meanwhile, compounds **1**, **2**, **3**, and **4** inhibited colony formation in HCT 116 cells by 20%, 27%, 18%, and 16%, respectively. Compound **2** possessed comparable inhibitory activity against colony formation of both types of cancer cell lines (Figure 4).

#### 2.2.3. The Effect of Compounds **1**–**4** on Migration of Human Cancer Cells

**Figure 5:**
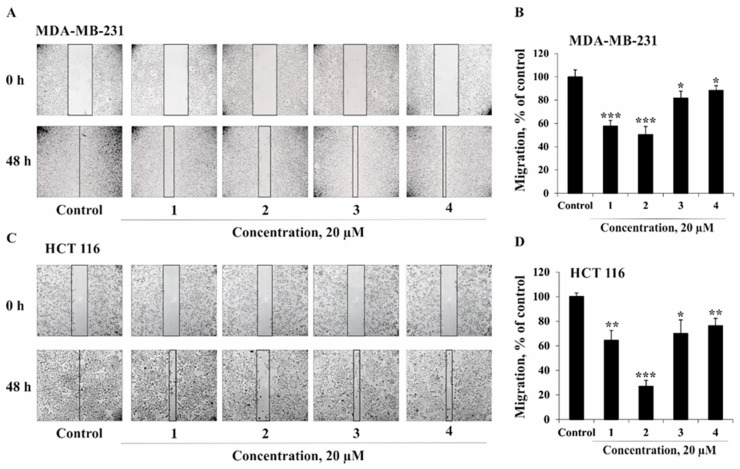
The effect of compounds **1**–**4** on migration of human cancer cells. MDA-MB-231 (**A**,**B**) and HCT 116 (**C**,**D**) cells migration distance was estimated by measuring the width of the wound and expressed as a percentage of each control for the mean of wound closure area. All experiments were repeated at least three times in each group (n = 18 for control and each compound, n—quantity of photos). The magnification of representative photos is ×10. Results are expressed as the mean ± standard deviation (SD). The asterisks (* *p* < 0.05, ** *p* < 0.01, *** *p* < 0.001) indicate a significant decrease in migration of cells treated with compounds compared with the control.

The metastasis process is proven to be the leading cause of cancer-related death. Metastasis is a multistep process that includes migration and invasion of cancer cells, hallmarks of malignancy [19]. Therefore, we investigated the ability of compounds **1**–**4** to inhibit the migration of breast cancer MDA-MB-231 cells and colorectal carcinoma HCT 116 cells with high metastatic potential. It was demonstrated that compounds **1** and **2** (at concentration 20 µM) were able to prevent migration of MDA-MB-231 cells by 42% and 50%, respectively, compared to control after 48 h of incubation (Figure 5). Compounds **3** and **4** possessed moderate inhibitory activity against migration of MDA-MB-231 cells. On the other hand, the migration of HCT 116 cells was almost completely inhibited by compound **2** (with the percentage of migration prevention being 73%). Compounds **1**, **3**, and **4** prevented HCT 116 cell migration by 36%, 30%, and 24%, respectively (Figure 5). Compounds **1**, **3**, and **4** prevented HCT 116 cell migration by 36%, 30%, and 24%, respectively (Figure 5).

## 3. Materials and Methods

### 3.1. General Procedures

Optical rotations were determined on a PerkinElmer 343 polarimeter (Waltham, MA, USA). IR spectra were determined on a Bruker OPUS Vector-22 infrared spectrophotometer in CDCl_3_. The ^1^H- and ^13^C-NMR spectra were recorded on Bruker Avance III 700 spectrometer (Bruker, Germany) at 700.13 and 176.04 MHz, respectively, and chemical shifts were referenced to the corresponding residual solvent signal (*δ*_H_ 3.30/*δ*_C_ 49.0 for CD_3_OD). The HRESIMS spectra were recorded on a Bruker Impact II Q-TOF mass spectrometer (Bruker, Germany); the samples were dissolved in MeOH (*c* 0.001 mg/mL). HPLC separations were carried out on an Agilent 1100 Series chromatograph (Agilent Technologies, Santa Clara, CA, USA) equipped with a differential refractometer; Discovery C18 (5 µm, 250 × 10 mm, Supelco, Bellefonte, PA, USA) column was used. GC and GC-MS analyses were performed on a GC 2010 chromatograph with a flame ionization detector and a gas chromatograph couple to a mass spectrometer GCMS-QP5050, both from Shimadzu (Japan). Fused silica capillary columns Supelcowax 10 and MDN-5S (both columns 30 m, 0.25 mm ID, 0.25 µm film, Supelco, PA, USA) were used in the apparatus. Low-pressure liquid column chromatography was carried out with the Si gel KSK (50–160 µm, Sorbpolimer, Krasnodar, Russia). Sorbfil Si gel plates (4.5 × 6.0 cm, 5–17 µm, Sorbpolimer, Krasnodar, Russia) were used for thin-layer chromatography.

### 3.2. Animal Material

Specimens of *Ceramaster patagonicus* Sladen, 1889 (order Valvatida, family Goniasteridae), were collected at a depth of 150–300 m in the Sea of Okhotsk near Iturup Island during 42nd scientific cruise of the research vessel *Akademik Oparin*, in August 2012. Species identification was carried out by B. B. Grebnev (G. B. Elyakov Pacific Institute of Bioorganic Chemistry FEB RAS, Vladivostok, Russia). A voucher specimen (No. 042-67) is on deposit at the marine specimen collection of the G. B. Elyakov Pacific Institute of Bioorganic Chemistry FEB RAS, Vladivostok, Russia.

### 3.3. Extraction and Isolation

The fresh animals of *C. patagonicus* (3 kg, crude weight) were chopped into small pieces and extracted with CHCl_3_:MeOH (2:1) followed by further extraction with CHCl_3_:MeOH (1:1) and EtOH. The combined extracts were concentrated in vacuo to give a residue of 159.5 g. This residue was partitioned between H_2_O (1.5 L) and AcOEt:BuOH (2:1) (4.5 L), and the organic layer was concentrated in vacuo to give the less polar fraction (51.5 g), which was washed with cold acetone (1 L). The acetone-soluble part (28.5 g) was chromatographed over a silica gel column (19 × 4.5 cm) using CHCl_3_, CHCl_3_:MeOH (97:3), and CHCl_3_:MeOH (9:1) to yield four fractions: 1 (932 mg), 2 (486 mg), 3 (735 mg), and 4 (1.04 g). Fractions 1–4 were further chromatographed over a Si gel column (10 × 4 cm) using *n*-hexane:AcOEt:MeOH (stepwise gradient, 6:3:0.1→6:3:0.7, *v/v/v*) to yield six subfractions: 21 (123 mg), 31 (475 mg), 32 (231 mg), 41 (55 mg), 42 (212 mg), and 43 (570 mg), which were then analyzed by TLC in the eluent system CHCl_3_:MeOH:H_2_O (8:1:0.1, *v/v/v*). Subfractions 21–43 mainly contained the ceramides, cerebrosides, admixtures of pigments, and concomitant other lipids. HPLC separation of subfraction 32 (231 mg) on a Diasfer-110-C18 column (2.5 mL/min) with MeOH as an eluent yielded pure **1** (4.4 mg, R_t_ 42.3 min), **2** (2.5 mg, R_t_ 51.9 min), **3** (3.9 mg, R_t_ 55.7 min), and **4** (2.9 mg, R_t_ 72.5 min).

### 3.4. Compound Characterization Data

(25*S*)-5α-Cholestane-3β,6β,15α,16β-tetraol-26-yl 5′*Z*,11′*Z*-octadecadienoate (**1**): Amorphous powder; [α]_D_^25^: +8.1 (*c* 0.44, MeOH); IR (CDCl_3_) *ν*_max_ 3504, 2929, 2856, 1723, 1602, 1459, 1379, 1216, 1042 cm^−1^; (+)HRESIMS *m/z* 737.5695 [M + Na]^+^ (calcd for C_45_H_78_O_6_Na, 737.5695); (–)HRESIMS *m/z* 773.5934 [(M − H) + AcOH]^−^ (calcd for C_47_H_81_O_8_, 773.5937), 713.5726 [M − H]^−^ (calcd for C_45_H_77_O_6_, 713.5729); (–)ESIMS/MS of the ion at *m*/*z* 713: *m*/*z* 449 [C_27_H_45_O_5_]^−^, 279 [C_18_H_31_O_2_]^−^; ^1^H- and ^13^C-NMR data, see Table 1 and Table 2.

(25*S*)-5α-Cholestane-3β,6β,15α,16β-tetraol-26-yl 11′*Z*-octadecenoate (**2**): Amorphous powder; [α]_D_^25^: +14.1 (*c* 0.25, MeOH); IR (CDCl_3_) *ν*_max_ 3504, 2929, 2856, 1723, 1602, 1465, 1379, 1260, 1216, 1043 cm^−1^; (+)HRESIMS *m/z* 739.5852 [M + Na]^+^ (calcd for C_45_H_80_O_6_Na, 739.5847); (–)HRESIMS *m/z* 775.6096 [(M − H) + AcOH]^−^ (calcd for C_47_H_83_O_8_, 775.6093); *m/z* 715.5889 [M − H]^−^ (calcd for C_45_H_79_O_6_, 715.5882); (–)ESIMS/MS of the ion at *m*/*z* 715: *m*/*z* 449 [C_27_H_45_O_5_]^−^, 281 [C_18_H_33_O_2_]^−^; ^1^H- and ^13^C-NMR data, see Table 1 and Table 2.

(25*S*)-5α-Cholestane-3β,6β,15α,16β-tetraol-26-yl 5′*Z*,11′*Z*-eicosadienoate (**3**): Amorphous powder; [α]_D_^25^: +9.5 (*c* 0.39, MeOH); IR (CDCl_3_) *ν*_max_ 3510, 2929, 2856, 1723, 1603, 1493, 1465, 1379, 1365, 1248, 1216, 1189, 1080, 1042 cm^−1^; (+)HRESIMS *m/z* 765.6008 [M + Na]^+^ (calcd for C_47_H_82_O_6_Na, 765.6004); (–)HRESIMS *m/z* 801.6254 [(M − H) + AcOH]^−^ (calcd for C_49_H_85_O_8_, 801.6250); *m/z* 741.6044 [M − H]^−^ (calcd for C_47_H_81_O_6_, 741.6039); (–)ESIMS/MS of the ion at *m*/*z* 741: *m*/*z* 449 [C_27_H_45_O_5_]^−^, 307 [C_20_H_35_O_2_]^−^; ^1^H- and ^13^C-NMR data, see Table 1 and Table 2.

(25*S*)-5α-Cholestane-3β,6β,15α,16β-tetraol-26-yl 7′*Z*-eicosenoate (**4**): Amorphous powder; [α]_D_^25^: +15.4 (*c* 0.29, MeOH); IR (CDCl_3_) *ν*_max_ 3515, 2929, 2856, 1723, 1602, 1466, 1378, 1261, 1204, 1043 cm^−1^; (+)HRESIMS *m/z* 767.6157 [M + Na]^+^ (calcd for C_47_H_84_O_6_Na, 767.6160); (–)HRESIMS *m/z* 803.6409 [(M − H) + AcOH]^−^ (calcd for C_49_H_87_O_8_, 803.6406); *m/z* 743.6199 [M − H]^−^ (calcd for C_47_H_83_O_6_, 743.6195); (–)ESIMS/MS of the ion at *m*/*z* 743: *m*/*z* 449 [C_27_H_45_O_5_]^−^, 309 [C_20_H_37_O_2_]^−^; ^1^H- and ^13^C-NMR data, see Table 1 and Table 2.

### 3.5. Methanolysis and Preparation of 4,4-Dimethyloxazoline Derivatives of Fatty Acids

Compounds **1**–**4** (1 mg) were heated with 1 N HCl in 80% aq MeOH (1.0 mL) at 80 °C for 4 h. The reaction mixtures were then extracted with *n*-hexane, and the extracts were concentrated in vacuo to yield FAME-1–FAME-4. The 4,4-Dimethyloxazoline derivatives of fatty acids of compounds **1** and **4** were prepared from FAME-1 and FAME-4 according to the procedure described previously [15].

### 3.6. FAME and DMOX Analysis.

FAMEs were analyzed on Supelcowax 10 columns at 200 °C. DMOX derivatives analyzed on a nonpolar MDN-5S column, where the temperature program ranged from 200 to 260 °C at 2 °C/min. Helium was used as the carrier gas at a linear velocity of 30 cm/s. Mass spectra were recorded at 70 eV. Mass spectra were compared with the NIST library and internet fatty acid mass spectra archive site.

### 3.7. Bioactivity Assay

#### 3.7.1. Reagents

Phosphate buffered saline (PBS), L-glutamine, penicillin–streptomycin solution (10,000 U/mL, 10 µg/mL) were from Sigma-Aldrich (St. Louis, MO, USA). MTS reagent (3-(4,5-dimethylthiazol-2-yl)-5-(3-carboxymethoxyphenyl)-2-(4-sulfophenyl)-2H-tetrazolium) was purchased from Promega (Madison, WI, USA). The Basal Medium Eagle (BME), Minimum Essential Medium Eagle (MEM), Dulbecco’s Modified Eagle’s Medium (DMEM), McCoy’s 5A Modified Medium (McCoy’s 5A), trypsin, fetal bovine serum (FBS), and agar were purchased from Thermo Fisher Scientific (Waltham, MA, USA).

#### 3.7.2. Cell Cultures

Mouse epidermal JB6 Cl41 (ATCC No. CRL-2010), human breast cancer MDA-MB-231 (ATCC HTB-26), and colorectal carcinoma HCT 116 (ATCC CCL-247) cell lines were cultured in MEM, DMEM, and McCoy’s 5A medium supplemented with 5%, 10%, and 10% FBS, respectively, and 1% penicillin–streptomycin solution. The cell cultures were maintained at 37 °C in humidified atmosphere containing 5% CO_2_.

#### 3.7.3. Compounds Preparation

Compounds **1**–**4** were dissolved in DMSO to prepare stock concentrations of 20 mM. Cells were treated with serially diluted compounds (culture medium used as diluent) to give the intended final concentrations (1, 10, 20, 50, and 100 µM). Solvent tolerance testing up to 0.5% of DMSO in control cells under identical conditions confirmed that the viability of all cell lines was unaffected. The vehicle control was the cells treated with equivalent volume of DMSO for all presented experiments.

#### 3.7.4. Cell Viability Assay

To determine the cytotoxicity of compounds **1**–**4**, JB6 Cl41, MDA-MB-231, and HCT 116 cells (1.0 × 10^4^) were seeded in 200 µL of complete MEM/5% FBS, DMEM/10% FBS, and McCoy’s 5A/10% FBS medium, respectively, and incubated for 24 h at 37 °C in 5% CO_2_ incubator. The attached cells were incubated with fresh medium containing various concentrations of **1**–**4** (0–100 µM) or equivalent volume of DMSO (control) for additional 24 h. Subsequently, the cells were incubated with 15 µL MTS reagent for 3 h, and the absorbance of each well was measured at 490/630 nm using Power Wave XS microplate reader (BioTek, Winooski, VT, USA). All experimental conditions were assessed in triplicate.

To determine the effect of compounds **1**–**4** on cell proliferation, the tested cell lines (8 × 10^3^ cells/200 µL) were treated with tested compounds at concentrations of 1, 10, 50, and 100 µM or equivalent volume of DMSO (control) and incubated for additional 24, 48, and 72 h at 37 °C in 5% CO_2_. MTS reagent (20 µL) was added to each well, and the cells were incubated for additional 3 h in 5% CO_2_ incubator. Absorbance was measured at 490/630 nm by microplate reader. All experimental conditions were assessed in triplicate.

#### 3.7.5. Soft Agar Assay

To estimate the effects of **1**–**4** on colony formation (phenotype expression), MDA-MB-231 and HCT 116 cells (2.4 × 10^4^ cells/200 µL) were treated equivalent volume of DMSO (control) or with compounds **1**–**4** (20 µM) in 1 mL of 0.3% Basal Medium Eagle (BME) agar containing 10% FBS, 2 mM L-glutamine, and 25 µg/mL gentamicin. The cultures were maintained in a 37 °C, 5% CO_2_ incubator for 14 days, and the cell colonies were scored using a Motic AE 20 microscope (XiangAn, Xiamen, China) and ImageJ software bundled with 64-bit Java 1.8.0_112 (NIH, Bethesda, MD, USA).

#### 3.7.6. Wound Healing Assay

MDA-MB-231 and HCT 116 cells (3 × 10^5^ cells/mL) were seeded into six-well plates and grown to 80% confluence for 24 h. After removing the culture medium, the cells’ monolayer was scraped with a 200 µL sterile pipette tip to create a straight scratch. Then, MDA-MB-231 and HCT 116 cells were treated with equivalent volume of DMSO (control) or **1**–**4** at concentration of 20 µM and incubated for 48 h. All experiments were conducted in triplicate for each group. For the image analysis, cell migration into the wound area was photographed at the stages of 0 and 48 h using a Motic AE 20 microscope and ImageJ software. The cell migration distance was estimated by measuring the width of the wound and expressed as a percentage of each control for the mean wound closure area.

#### 3.7.7. Statistical Analysis

All assays were performed using least three independent experiments. Results are expressed as the mean ± standard deviation (SD). Student’s *t*-test was used to evaluate the data with the following significance levels: * *p* < 0.05, ** *p* < 0.01, *** *p* < 0.001.

## 4. Conclusions

Four new steroidal conjugates, esters of polyhydroxysteroids with long-chain fatty acids (**1**–**4**), were isolated from the Far Eastern starfish *C. patagonicus.* Unusual compounds **1**–**4** contain the same 5α-cholestane-3β,6β,15α,16β,26-pentahydroxysteroidal moiety and differ from each other in fatty acid residues: 5′*Z*,11′*Z*-octadecadienoic (**1**), 11′*Z*-octadecenoic (**2**), 5′*Z*,11′*Z*-eicosadienoic (**3**), and 7′*Z*-eicosenoic (**4**) acid units. It should be noted that the isolated conjugates **1**–**4** have a shared steroidal part and differ in the composition of fatty acid residues. The question arises about the biological role of the extracted compounds. Previously we have been found that starfish polyhydroxylated steroids were presented mainly in digestive organs of starfishes during the whole year, and their maximum concentration coincided with periods of active nutrition of these animals [20,21]. Recently, we confirmed the digestive function of polyhydroxysteroids, when studying the distribution of polar steroids in various organs of the starfish *Lethasterias fusca* using the nLC/CSI–QTOF–MS method, and the highest level of polar steroids was found in the stomach and the pyloric caeca [22] Thus, it can be assumed that polyhydroxysteroids can bind food fatty acids and participate in their transport to peripheral tissues, like cholesterol of vertebrates and humans. This assumption is partially confirmed by the heterogeneous composition of fatty acids in compounds **1**–**4**, since saturated, mono- and di-unsaturated C16, C18, and C20 fatty acids were found together with the main components. The isolation of conjugates of polyhydroxysteroids and fatty acids from the Far Eastern starfish *C. patagonicus* is a very interesting finding; to the best of our knowledge, hypotheses about the possible transport role of polyhydroxysteroids have not been put forward. At the same time, this assumption requires confirmation by experimental data.

We have expanded the data on the biological activity of these unique compounds—conjugates of polyhydroxysteroids and fatty acids. It was shown that tested compounds **1**–**4** possessed cytostatic activity against normal JB6 Cl41 cells and cancer MDA-MB-231 and HCT 116 cell lines. The compounds **1**–**4** at low concentration of 20 µM were able to suppress the colony formation in MDA-MB-231 and HCT 116 cells and almost completely prevent the migration of human breast and colorectal cancer cells. It should be noted that compound **2** was the most active in all experiments performed. This is likely due to the presence of an 11′*Z*-octadecenoic acid residue in its structure. Unfortunately, the lack of selectivity of the investigated compounds against normal and cancer cells was determined, which can be limit their possible practical use. However, the identification of these novel compounds and an initial characterization of their biological activity, performed for the first time, might be helpful to other researchers working on the development of conjugates of polyhydroxysteroids and fatty acids.

## Figures and Tables

**Table 1:** ^1^H- (700.13 MHz) and ^13^C- (176.04 MHz) NMR chemical shifts of the steroidal moiety of **1**–**4** in C_5_D_5_N at 30 °C, with *δ* in ppm and *J* values in Hz.

Position	*δ*_H_	*δ*_C_	Position	*δ*_H_	*δ*_C_
1	1.76 dt (11.4, 3.5) 1.10 m	39.1	15	4.40 brd (10.0)	84.7
2	2.13 m 1.86 m	32.5	16	4.66 brd (7.3)	82.2
3	3.99 m	71.1	17	1.55 dd (11.1, 7.3)	59.3
4	2.45 m 2.04 brd (12.2)	37.0	18	1.27 s	14.9
5	1.35 m	48.3	19	1.47 s	16.2
6	4.15 brd (2.6)	71.2	20	2.35 m	30.0
7	2.95 dt (14.2, 3.5) 1.82 m	41.2	21	1.13 d (6.8)	18.3
8	2.55 qd (11.1, 3.5)	30.8	22	1.92 m 1.28 m	36.4
9	0.96 m	54.9	23	1.60 m 1.39 m	23.9
10	−	36.0	24	1.41 m 1.17 m	34.0
11	1.64 m 1.59 m	21.2	25	1.82 m	32.8
12	2.10 m 1.36 m	41.1	26	4.11 dd (10.6, 5.4) 3.96 dd (10.6, 6.8)	69.1
13	−	43.9	27	0.94 d (6.8)	17.0
14	1.45 t (10.4)	61.0			

δ_H_-chemical shift of proton (ppm); δ_C_-chemical shift of carbon (ppm); s-singlet; d-doublet; t-triplet; m-multiplet; brd-broad doublet; dd-doublet of doublets; dt-doublet of triplets; qd-quartet of doublets.

**Table 2:** ^1^H- (700.13 MHz) and ^13^C- (176.04 MHz) NMR chemical shifts of the fatty acid units of **1**–**4** in C_5_D_5_N at 30 °C, with *δ* in ppm and *J* values in Hz.

Position	1		2		3		4	
	*δ*_H_	*δ*_C_	*δ*_H_	*δ*_C_	*δ*_H_	*δ*_C_	*δ*_H_	*δ*_C_
1′	−	173.3	−	173.3	−	173.1	−	173.3
2′	2.43 t (7.4)	33.7	2.40 t (7.5)	34.3	2.43 t (7.2)	33.6	2.40 t (7.5)	34.2
3′	1.80 m	25.2	1.70 m	25.1	1.80 m	25.2	1.71 m	25.1
4′	2.17 m	26.7			2.17 q (7.2)	26.7		
5′	5.44 m	128.9			5.46 m	128.9		
6′	5.51 m	130.9			5.51 m	130.9	2.11 m	27.3
7′	2.10 m	27.4			2.10 m	27.4	5.50 m	130.2
10′	2.11 m	27.4	2.11 m	27.3	2.11 m	27.4	5.48 m	129.7
11′	5.48 m	129.8	5.49 m	130.0	5.49 m	129.8	2.11 m	27.1
12′	5.49 m	130.1	5.49 m	130.0	5.50 m	130.1		
13′	2.11 m	27.1	2.11 m	27.1	2.11 m	27.1		
16′ or 18′	1.25 m	31.7	1.25 m	31.8	1.25 m	31.9	1.25 m	31.8
17′ or 19′	1.28 m	22.7	1.28 m	22.8	1.28 m	22.7	1.28 m	22.8
18′ or 20′	0.88 t (6.9)	14.0	0.87 t (6.9)	14.0	0.88 t (7.0)	14.0	0.89 t (6.9)	14.0

**Table 3:** Fatty acid composition of compounds **1**–**4** based on GC-MS analysis.

Fatty Acids	Content, %			
	1	2	3	4
16:0	14.91	9.36	11.83	5.88
Δ^7^-16:1	5.11	0.75	5.06	1.07
18:0	11.23	8.92	8.13	8.03
Δ^5^-18:1	1.62	4.62	17.05	0.57
Δ^9^-18:1	5.97	6.53	6.65	4.83
Δ^11^-18:1	4.06	**51.16**	3.92	2.05
Δ^5,11^-18:2	**53.31**		3.73	6.14
Δ^7^-20:1		8.86	3.50	**66.67**
Δ^9^-20:1			0.63	3.88
Δ^5,11^-20:2	3.78	9.79	**39.51**	0.87
**Total**	100.00	100.00	100.00	100.00

## Supplementary Materials

The following are available online at https://www.mdpi.com/1660-3397/18/5/260/s1. Copies (+)HRESIMS (Figures S1, S21, S30, and S39), (–)HRESIMS (Figures S2, S22, S31, and S40), (–)ESIMS/MS (Figures S3, S23, S32, and S41), ^1^H-NMR (Figures S4–S8, S24, S33, and S42), ^13^C-NMR (Figures S9–S12, S25, S34, and S43), COSY (Figures S13, S14, S26, S35, and S44), HSQC (Figures S15, S16, S27, S36, and S45), HMBC (Figures S17, S18, S28, S37, and S46), and ROESY (Figures S19, S20, S29, S38, and S47) spectra of compounds **1**, **2**, **3**, and **4**, respectively.
