# Supplementary material for: New Conjugates of Polyhydroxysteroids with Long-Chain Fatty Acids from the Deep-Water Far Eastern Starfish Ceramaster patagonicus and Their Anticancer Activity
Source: Mar Drugs. 2020 May 15;18(5):260. doi: 10.3390/md18050260 (PMC7281419; doi:10.3390/md18050260)

Supplementary Materials

New Conjugates of Polyhydroxysteroids with Long-Chain Fatty Acids from the Deep-Water Far Eastern Starfish *Ceramaster patagonicus* and Their Anticancer Activity

Timofey V. Malyarenko[1,2*], Alla A. Kicha[1], Olesya S. Malyarenko[1], Viktor M. Zakharenko[2], Ivan P. Kotlyarov[2], Anatoly I. Kalinovsky[1], Roman S. Popov[1], Vasily I. Svetashev[3], and Natalia V. Ivanchina[1]

1 G.B. Elyakov Pacific Institute of Bioorganic Chemistry, Far Eastern Branch of the Russian Academy of Sciences, Pr. 100-let Vladivostoku 159, 690022, Vladivostok, Russia; kicha@piboc.dvo.ru (A.A.K.); malyarenko.os@gmail.com (O.S.M.); kaaniw@piboc.dvo.ru (A.I.K.); prs_90@mail.ru (R.S.P.); ivanchina@piboc.dvo.ru (N.V.I.).

2 Department of Bioorganic Chemistry and Biotechnology, School of Natural Sciences, Far Eastern Federal University, Sukhanova str. 8, 690000, Vladivostok, Russia; rarf247@gmail.com (V.M.Z.); ivan_1999_19@icloud.com (I.P.K.).

3 A.V. Zhirmunsky National Scientific Center of Marine Biology, Far Eastern Branch of the Russian Academy of Sciences, 17 Palchevsky st., 690041, Vladivostok, Russia; vsvetashev@mail.ru (V.I.S.).

List

Figure S1. (+)-HRESIMS spectrum of compound **1**.

Figure S2. (–)-HRESIMS spectrum of compound **1**.

Figure S3. (–)-ESIMS/MS spectrum of compound **1**.

Figure S4. ^{1}H-NMR spectrum of compound **1** in C_5D_5N.

Figure S5. Enlarged-1 ^{1}H-NMR spectrum of compound **1** in C_5D_5N.

Figure S6. Enlarged-2 ^{1}H-NMR spectrum of compound **1** in C_5D_5N.

Figure S7. Enlarged-3 ^{1}H-NMR spectrum of compound **1** in C_5D_5N.

Figure S8. Enlarged-4 ^{1}H-NMR spectrum of compound **1** in C_5D_5N.

Figure S9. ^{13}C-NMR spectrum of compound **1** in C_5D_5N.

Figure S10. Enlarged-1 ^{13}C-NMR spectrum of compound **1** in C_5D_5N.

Figure S11. Enlarged-2 ^{13}C-NMR spectrum of compound **1** in C_5D_5N.

Figure S12. Enlarged-3 ^{13}C-NMR spectrum of compound **1** in C_5D_5N.

Figure S13. ^{1}H-^{1}H-COSY spectrum of compound **1** in C_5D_5N.

Figure S14. Enlarged ^{1}H-^{1}H-COSY spectrum of compound **1** in C_5D_5N.

Figure S15. HSQC spectrum of compound **1** in C_5D_5N.

Figure S16. Enlarged of HSQC spectrum of compound **1** in C_5D_5N.

Figure S17. HMBC spectrum of compound **1** in C_5D_5N.

Figure S18. Enlarged of HMBC spectrum of compound **1** in C_5D_5N.

Figure S19. ROESY spectrum of compound **1** in C_5D_5N.

Figure S20. Enlarged of ROESY spectrum of compound **1** in C_5D_5N.

Figure S21. (+)-HRESIMS spectrum of compound **2**.

Figure S22. (–)-HRESIMS spectrum of compound **2**.

Figure S23. (–)-ESIMS/MS spectrum of compound **2**.

Figure S24. ^{1}H-NMR spectrum of compound **2** in C_5D_5N.

Figure S25. ^{13}C-NMR spectrum of compound **2** in C_5D_5N.

Figure S26. ^{1}H-^{1}H-COSY spectrum of compound **2** in C_5D_5N.

Figure S27. HSQC spectrum of compound **2** in C_5D_5N.

Figure S28. HMBC spectrum of compound **2** in C_5D_5N.

Figure S29. ROESY spectrum of compound **2** in C_5D_5N.

Figure S30. (+)-HRESIMS spectrum of compound **3**.

Figure S31. (–)-HRESIMS spectrum of compound **3**.

Figure S32. (–)-ESIMS/MS spectrum of compound **3**.

Figure S33. ^{1}H-NMR spectrum of compound **3** in C_5D_5N.

Figure S34. ^{13}C-NMR spectrum of compound **3** in C_5D_5N.

Figure S35. ^{1}H-^{1}H-COSY spectrum of compound **3** in C_5D_5N.

Figure S36. HSQC spectrum of compound **3** in C_5D_5N.

Figure S37. HMBC spectrum of compound **3** in C_5D_5N.

Figure S38. ROESY spectrum of compound **3** in C_5D_5N.

Figure S39. (+)-HRESIMS spectrum of compound **4**.

Figure S40. (–)-HRESIMS spectrum of compound **4**.

Figure S41. (–)-ESIMS/MS spectrum of compound **4**.

Figure S42. ^{1}H-NMR spectrum of compound **4** in C_5D_5N.

Figure S43. ^{13}C-NMR spectrum of compound **4** in C_5D_5N.

Figure S44. ^{1}H-^{1}H-COSY spectrum of compound **4** in C_5D_5N.

Figure S45. HSQC spectrum of compound **4** in C_5D_5N.

Figure S46. HMBC spectrum of compound **4** in C_5D_5N.

Figure S47. ROESY spectrum of compound **4** in C_5D_5N.

Figure S48. Cytotoxicity compounds **1–4** after 24 h.

Figure S1. (+)-HRESIMS spectrum of compound **1**.

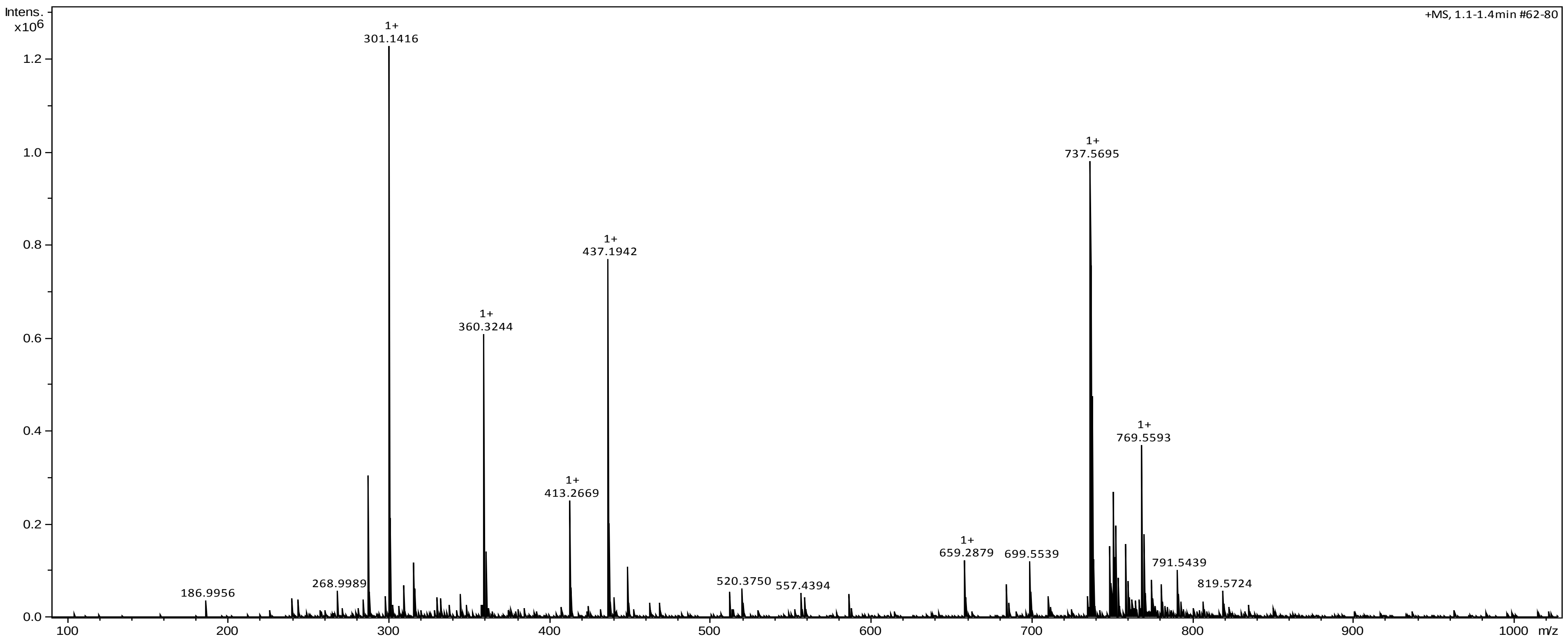

Figure S2. (–)-HRESIMS spectrum of compound **1**.

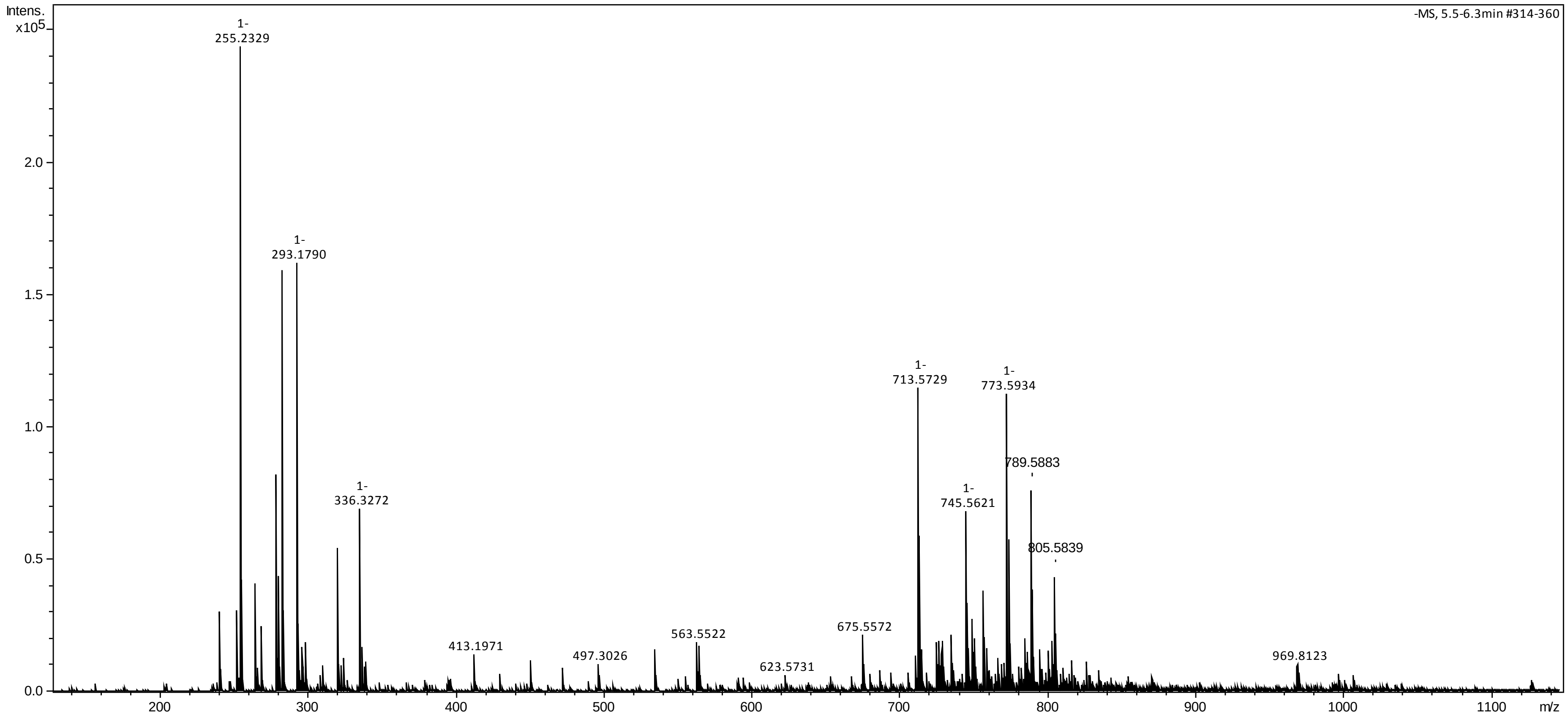

Figure S3. (–)-ESIMS/MS spectrum of compound **1**.

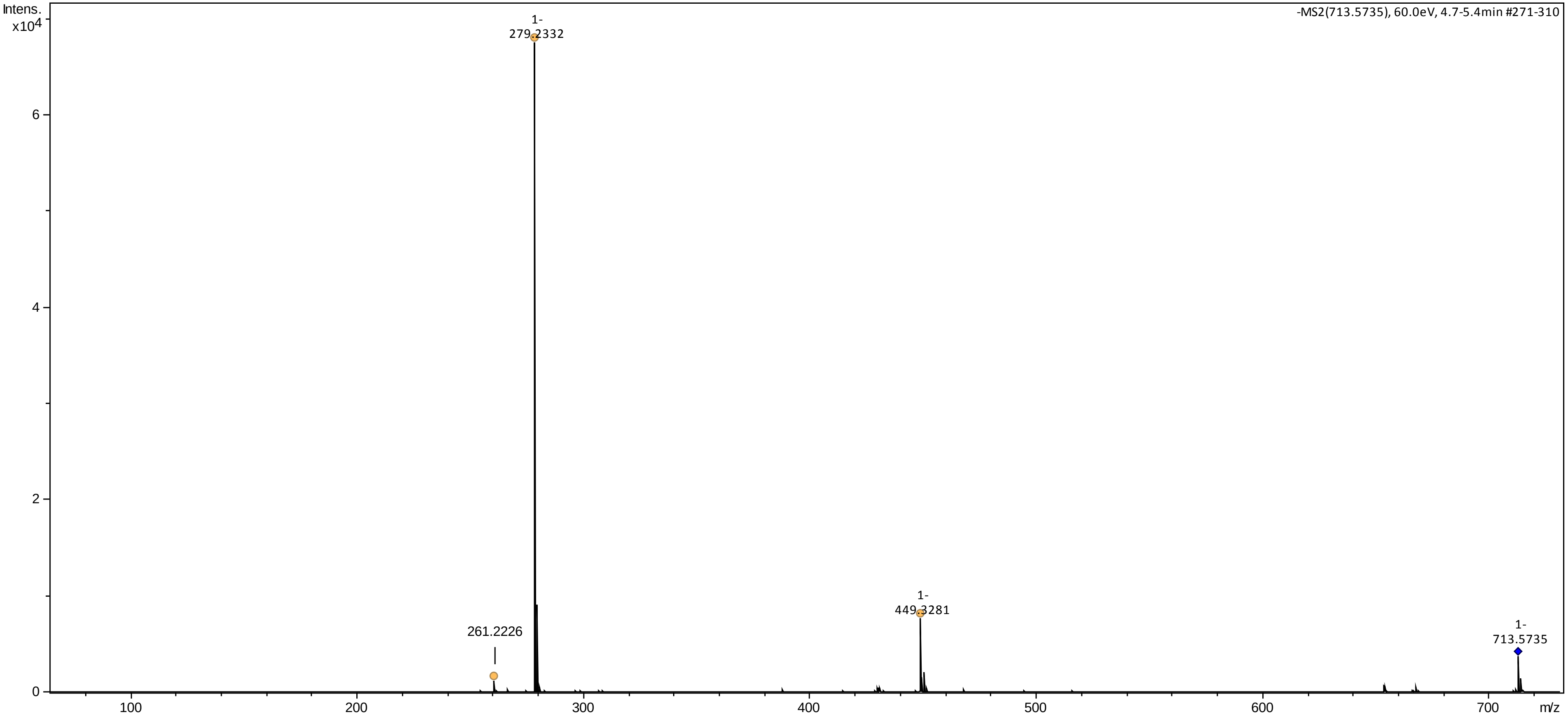

Figure S4. ^{1}H-NMR spectrum of compound **1** in C_5D_5N.

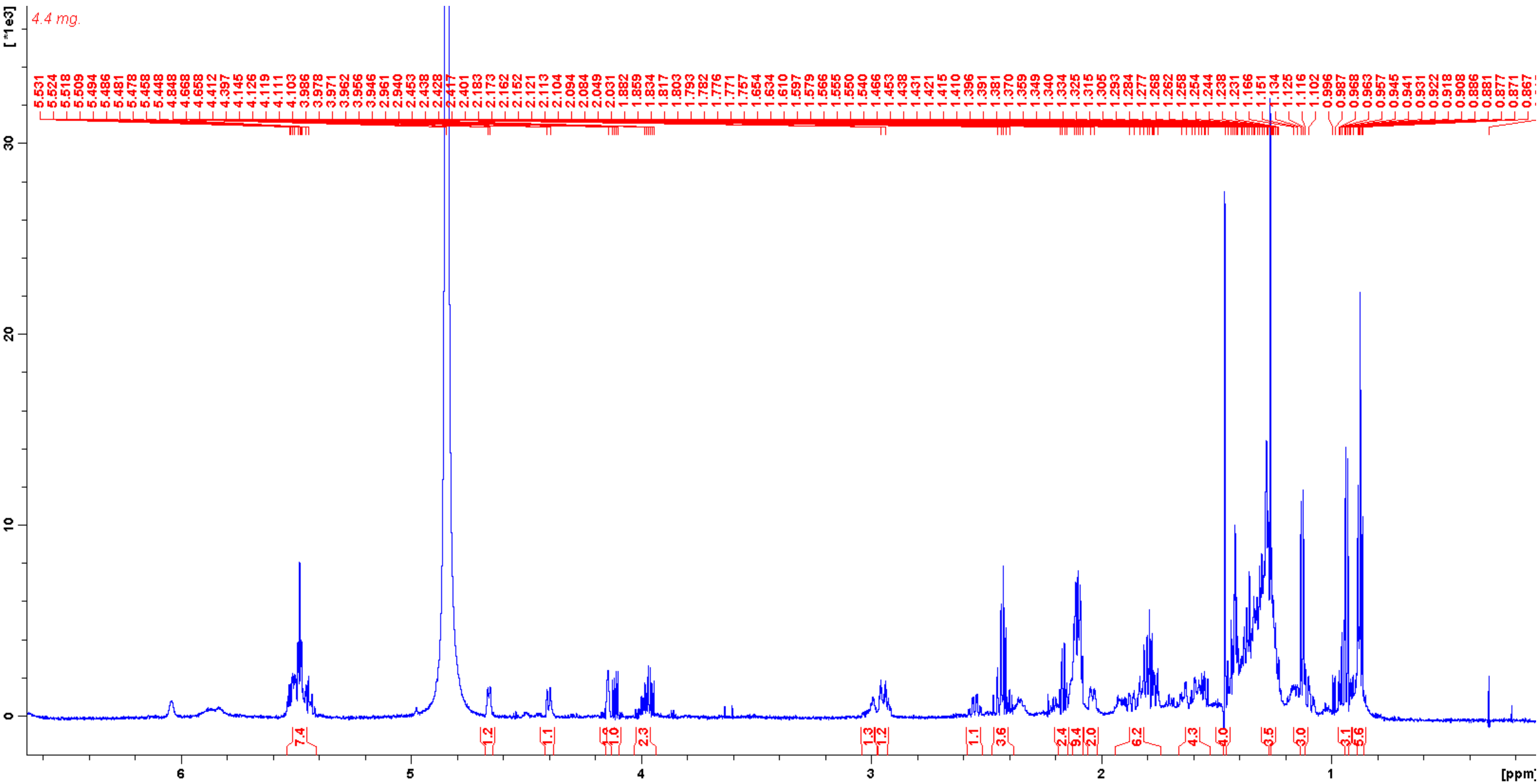

Figure S5. Enlarged-1 ^{1}H-NMR spectrum of compound **1** in C_5D_5N.

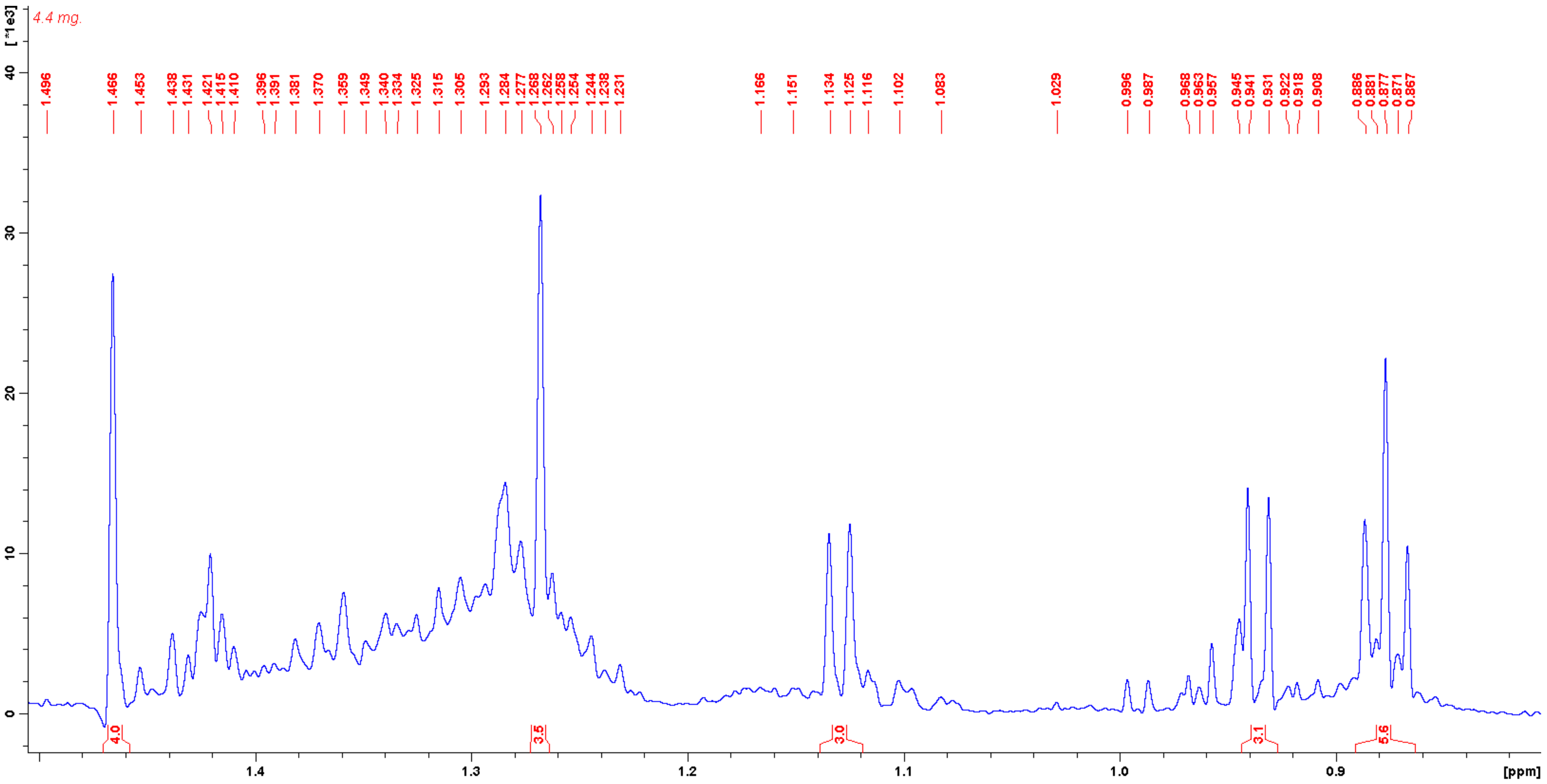

Figure S6. Enlarged-2 ^{1}H-NMR spectrum of compound **1** in C_5D_5N.

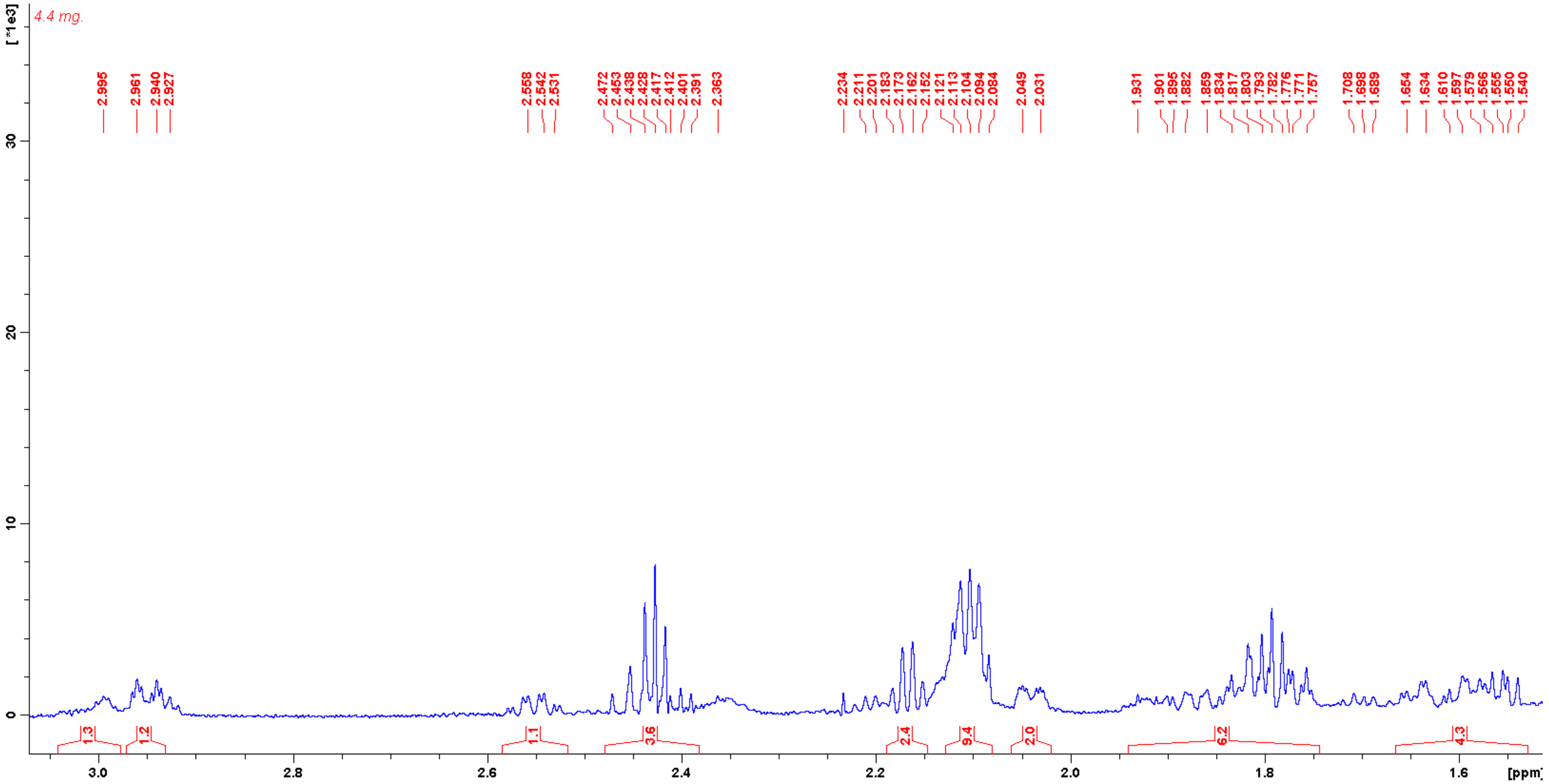

Figure S7. Enlarged-3 ^{1}H-NMR spectrum of compound **1** in C_5D_5N.

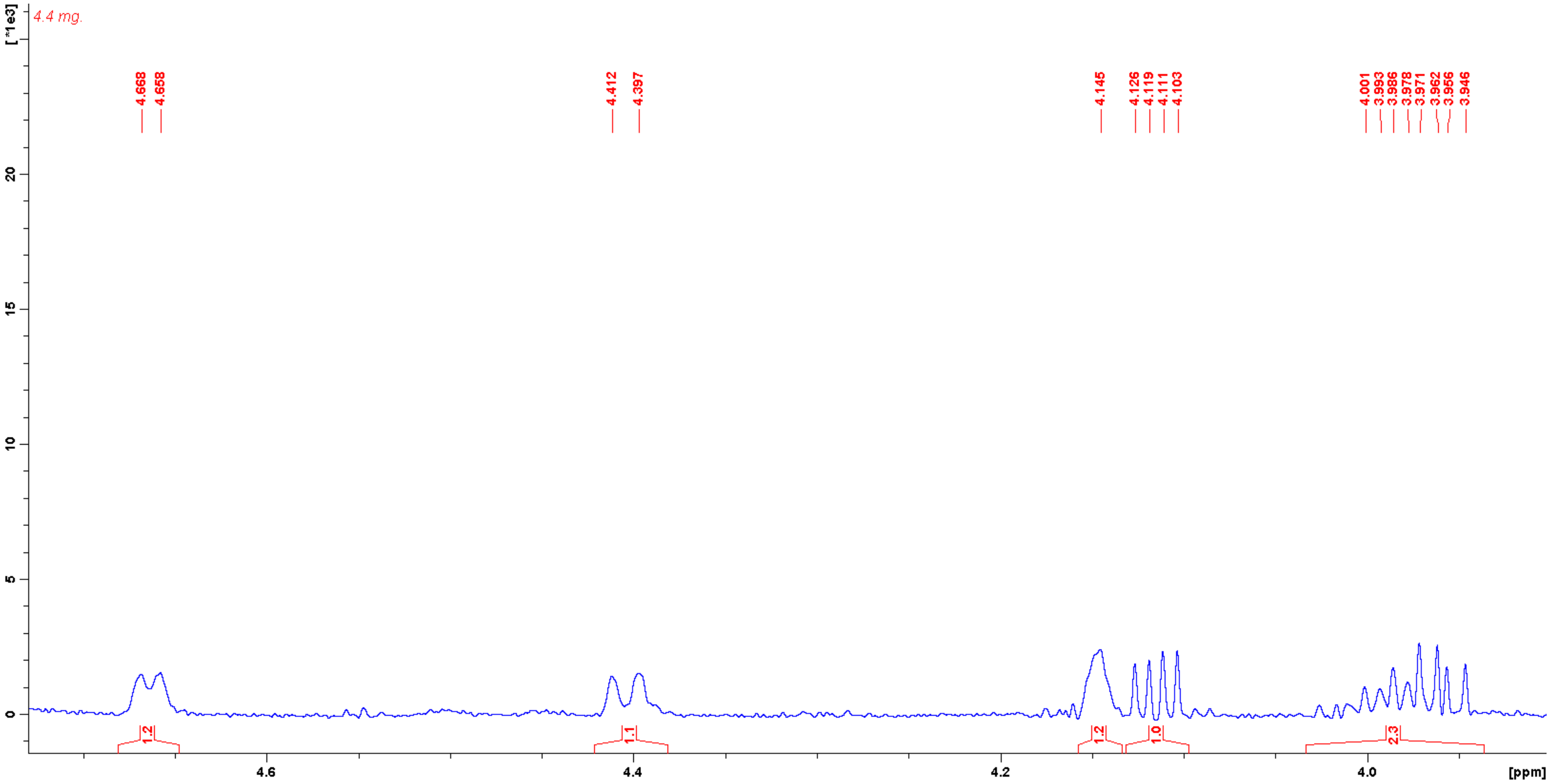

Figure S8. Enlarged-4 ^{1}H-NMR spectrum of compound **1** in C_5D_5N.

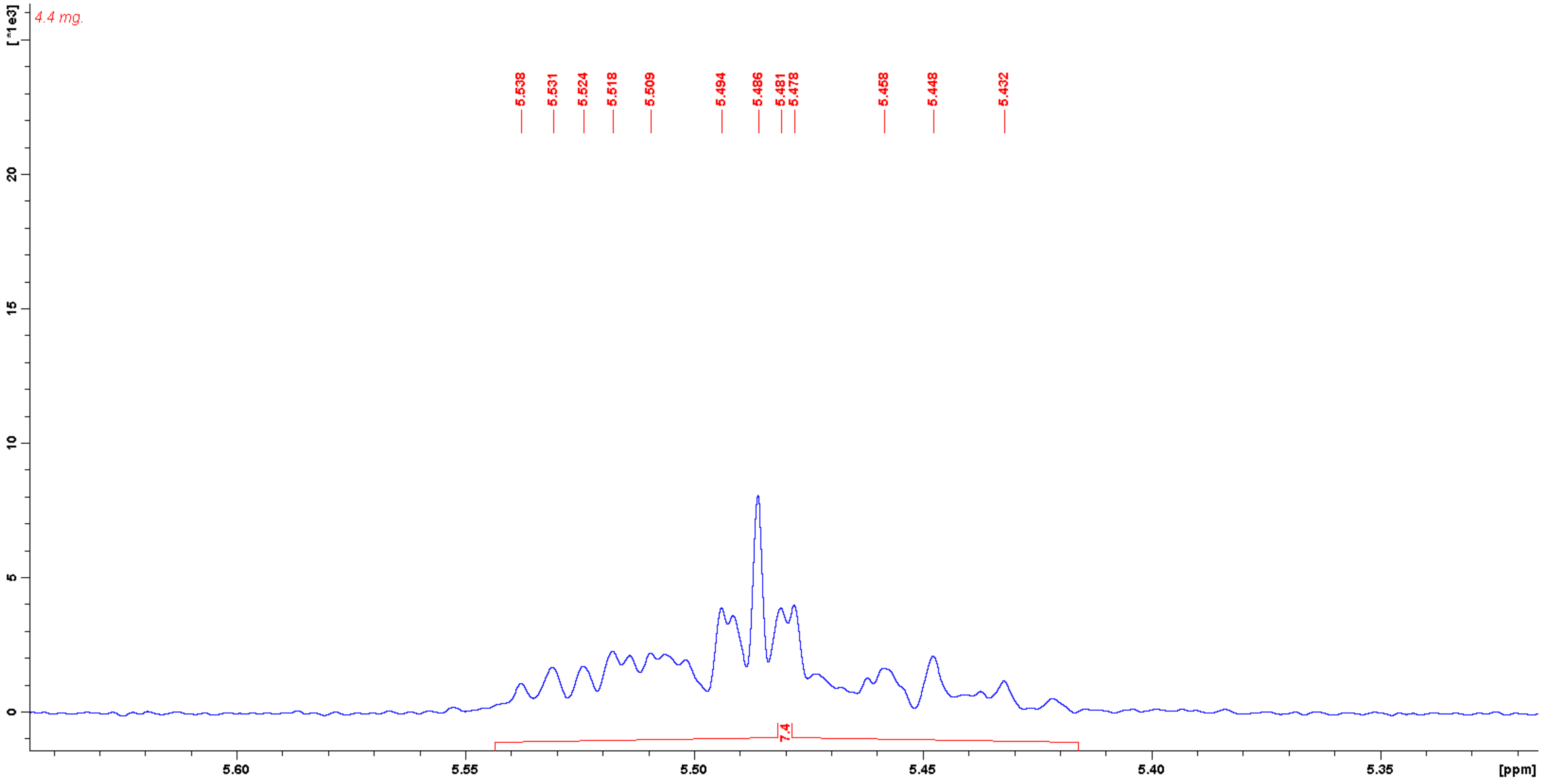

Figure S9. ^{13}C-NMR spectrum of compound **1** in C_5D_5N.

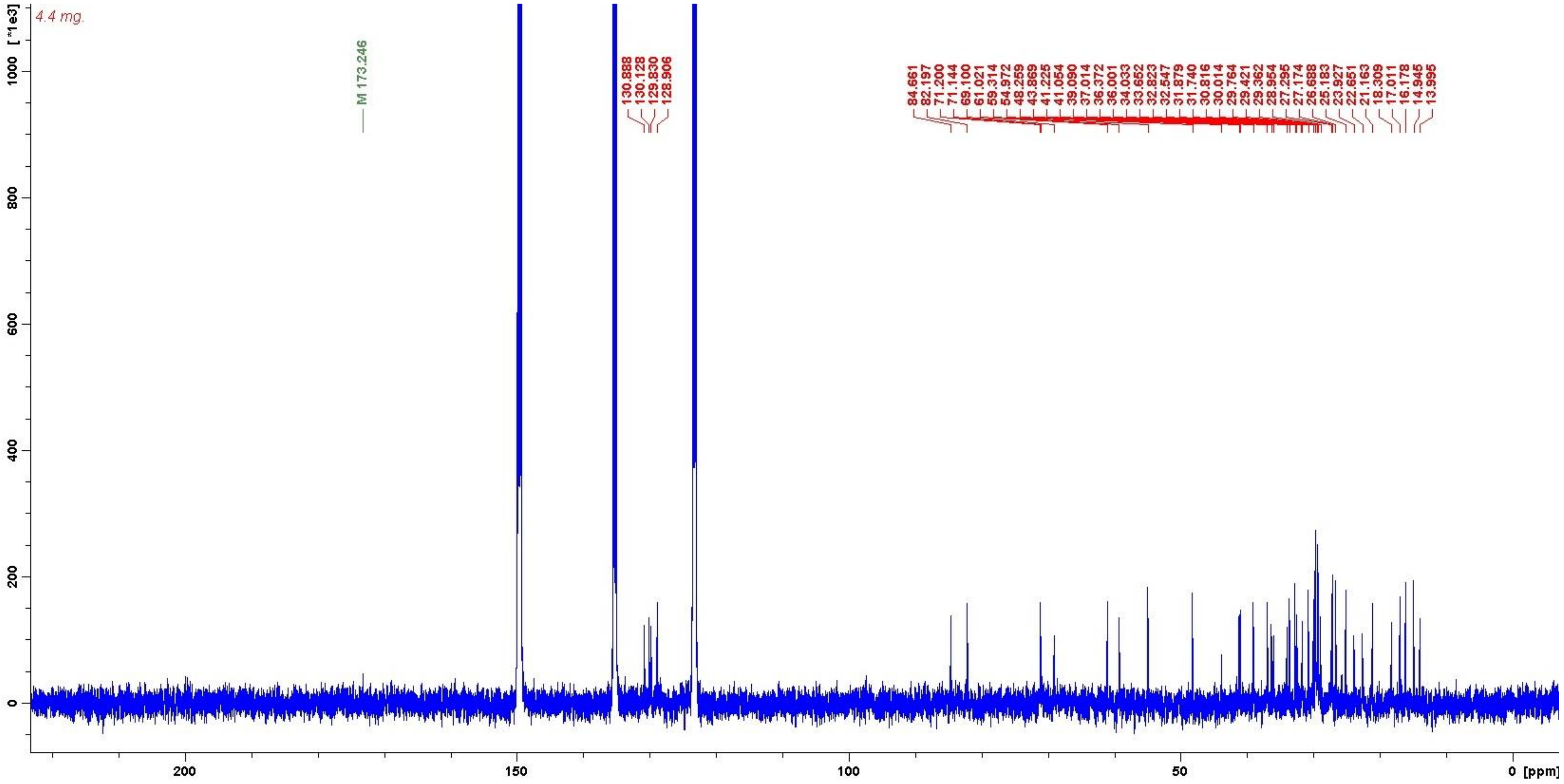

Figure S10. Enlarged-1 ^{13}C-NMR spectrum of compound **1** in C_5D_5N.

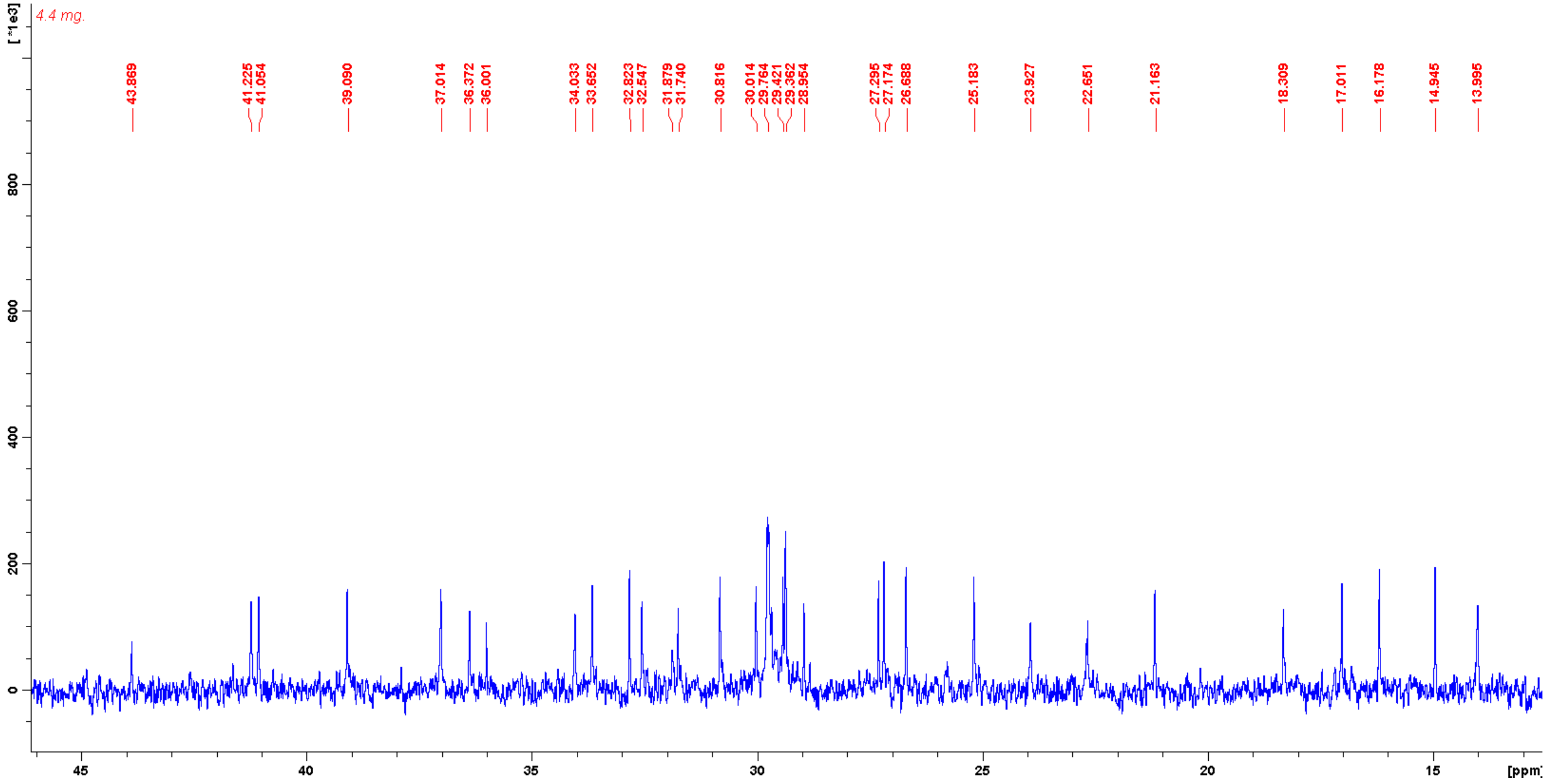

Figure S11. Enlarged-2 ^{13}C-NMR spectrum of compound **1** in C_5D_5N.

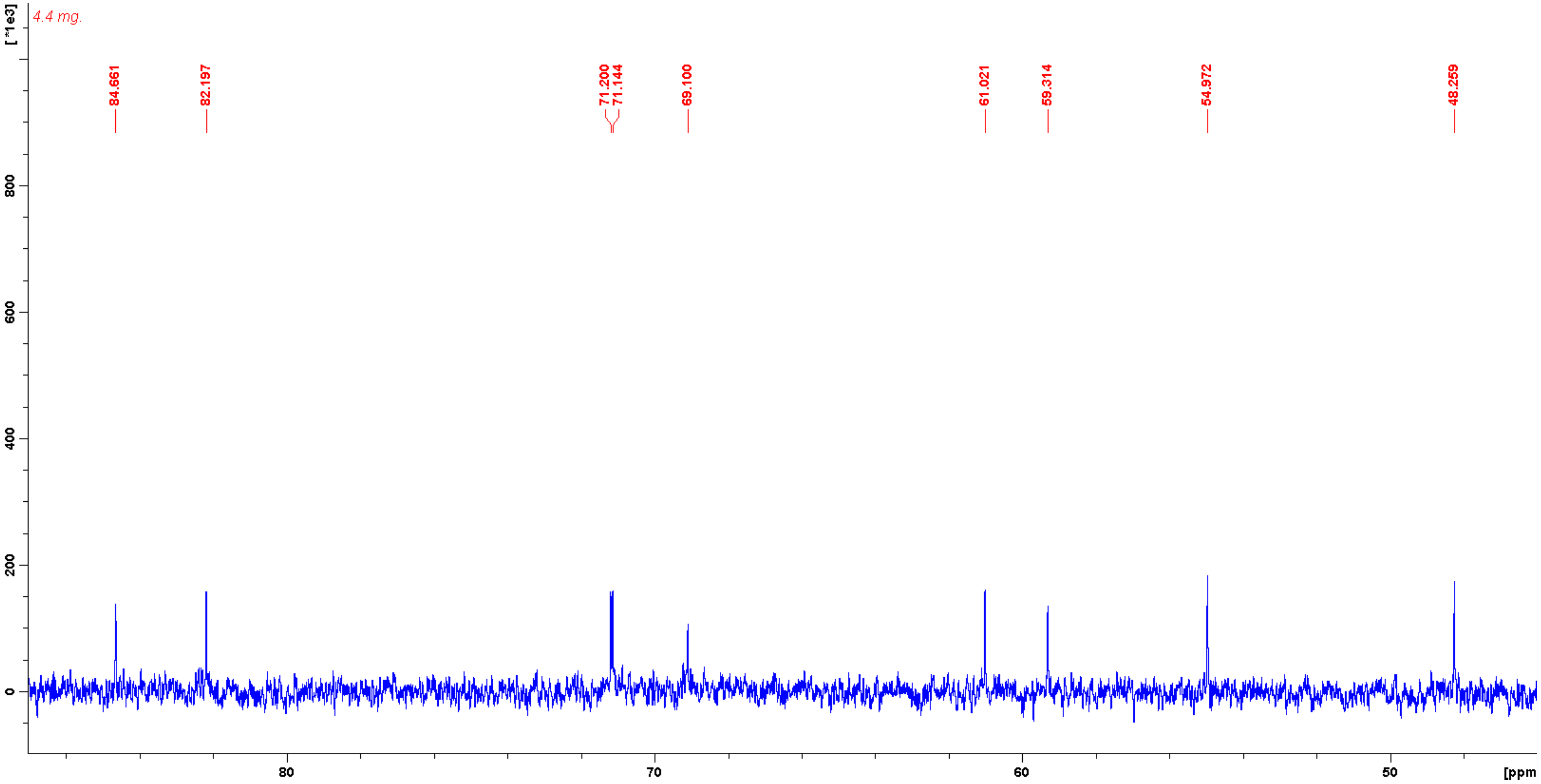

Figure S12. Enlarged-3 ^{13}C-NMR spectrum of compound **1** in C_5D_5N.

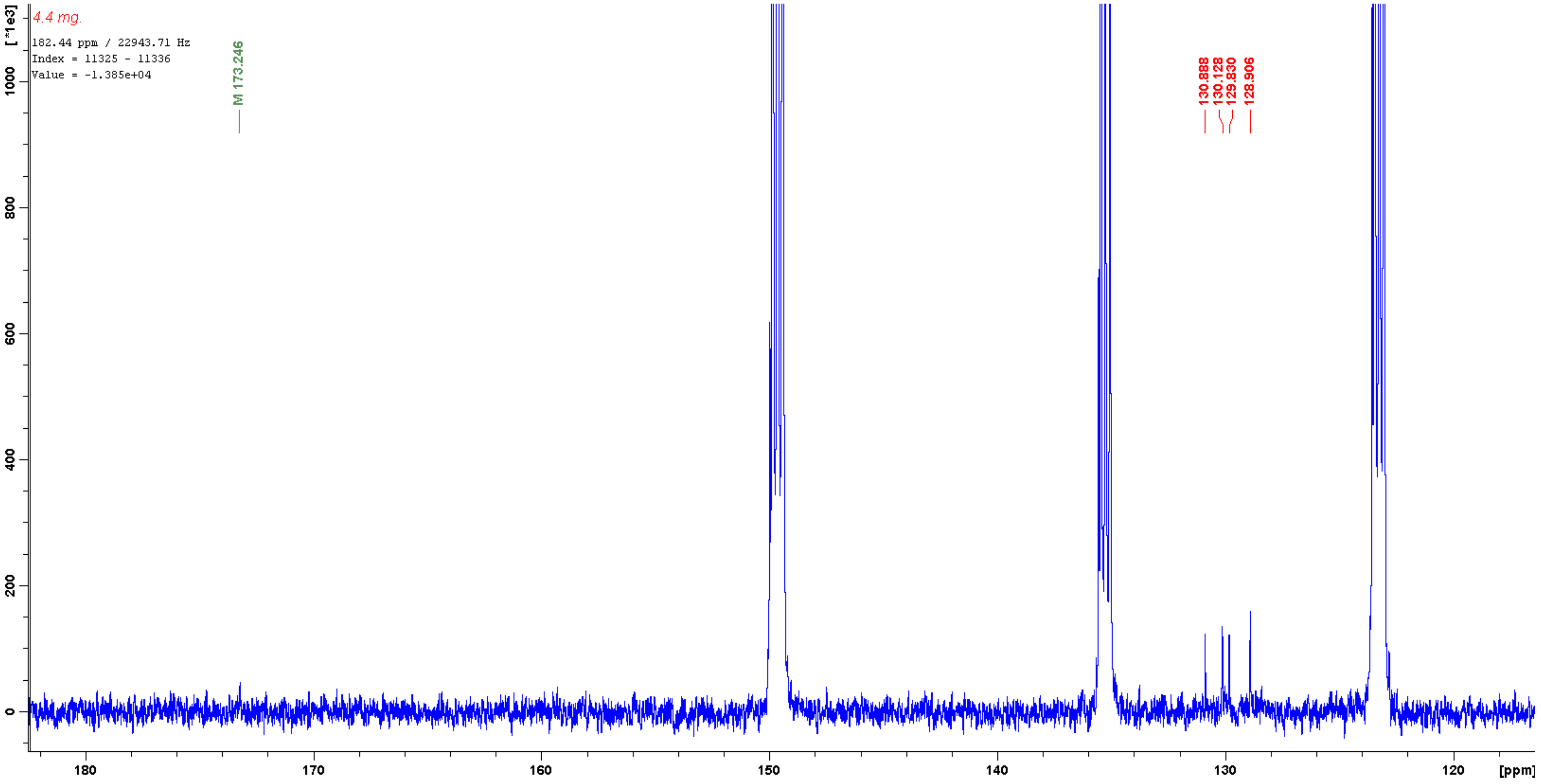

Figure S13. ^{1}H-^{1}H-COSY spectrum of compound **1** in C_5D_5N.

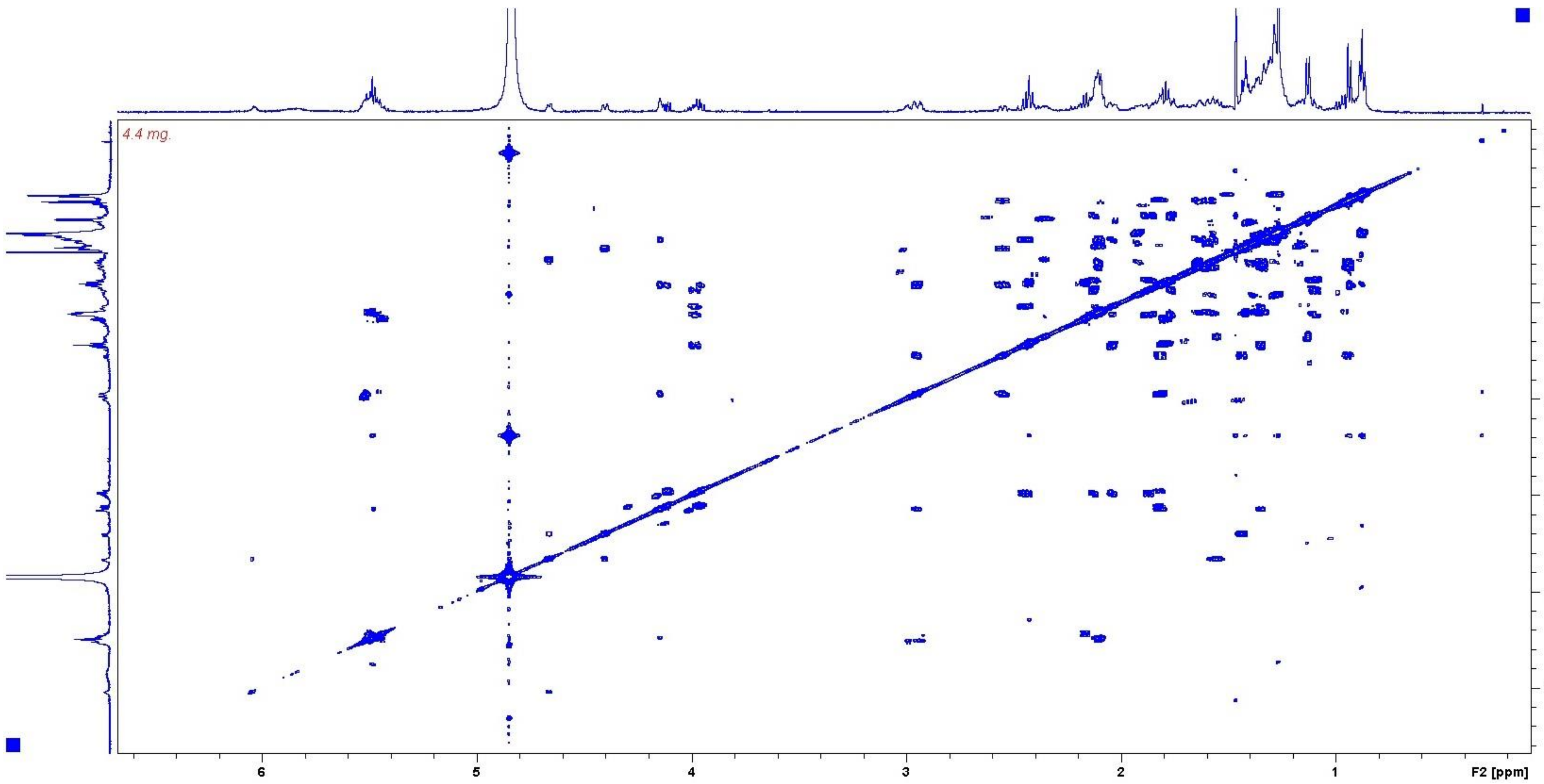

Figure S14. Enlarged ^{1}H-^{1}H-COSY spectrum of compound **1** in C_5D_5N.

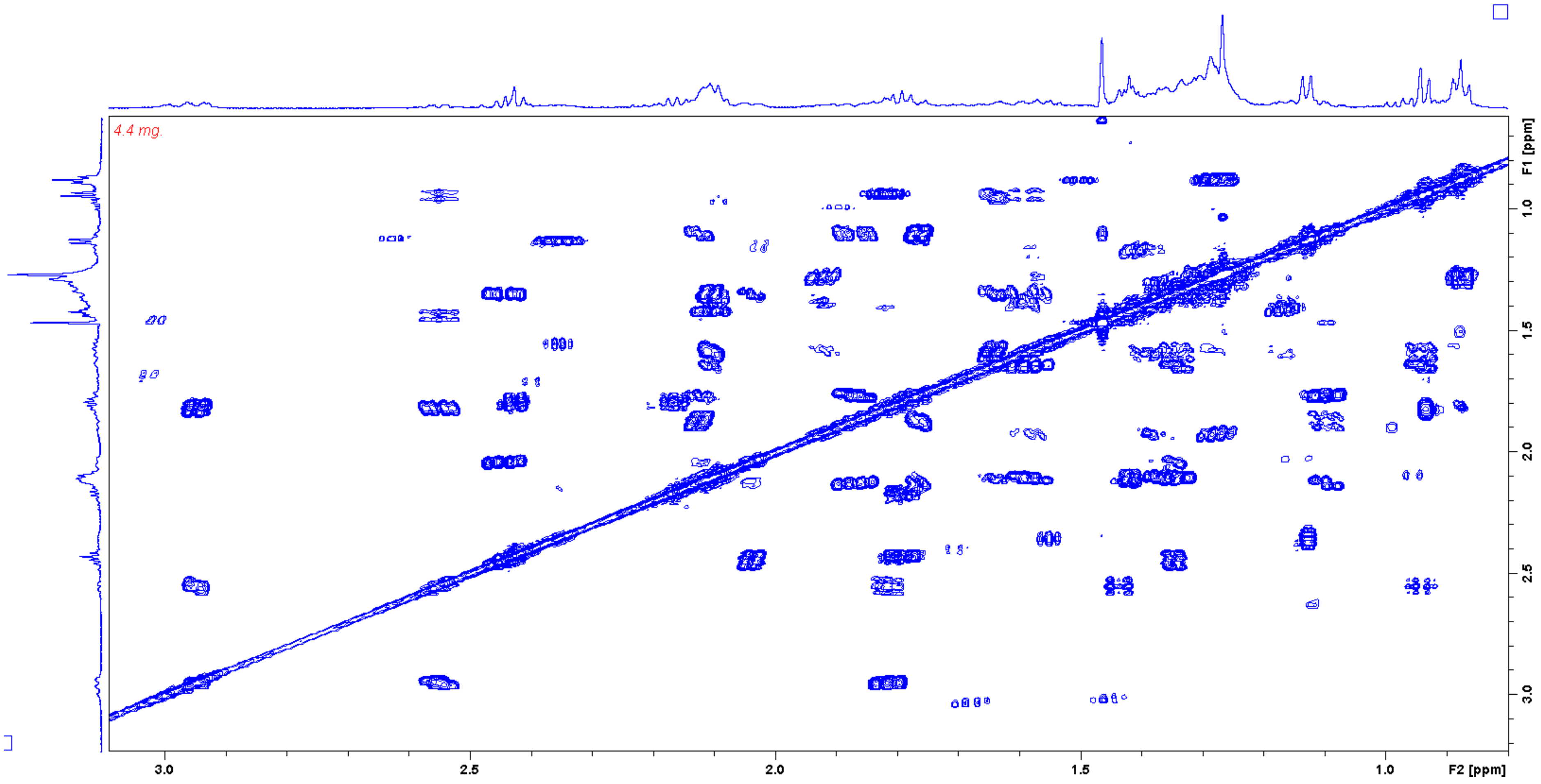

Figure S15. HSQC spectrum of compound **1** in C_5D_5N.

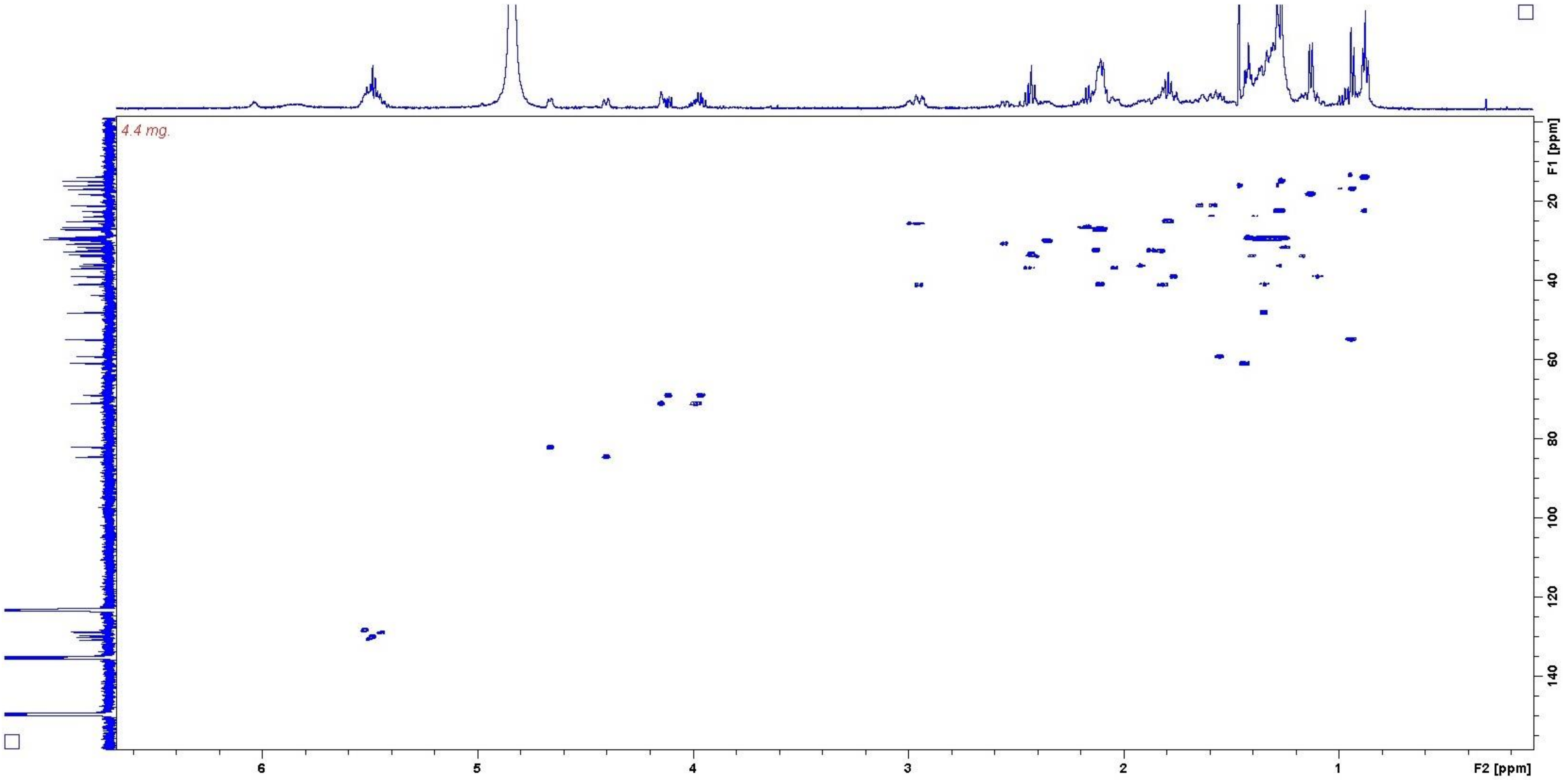

Figure S16. Enlarged HSQC spectrum of compound **1** in C_5D_5N.

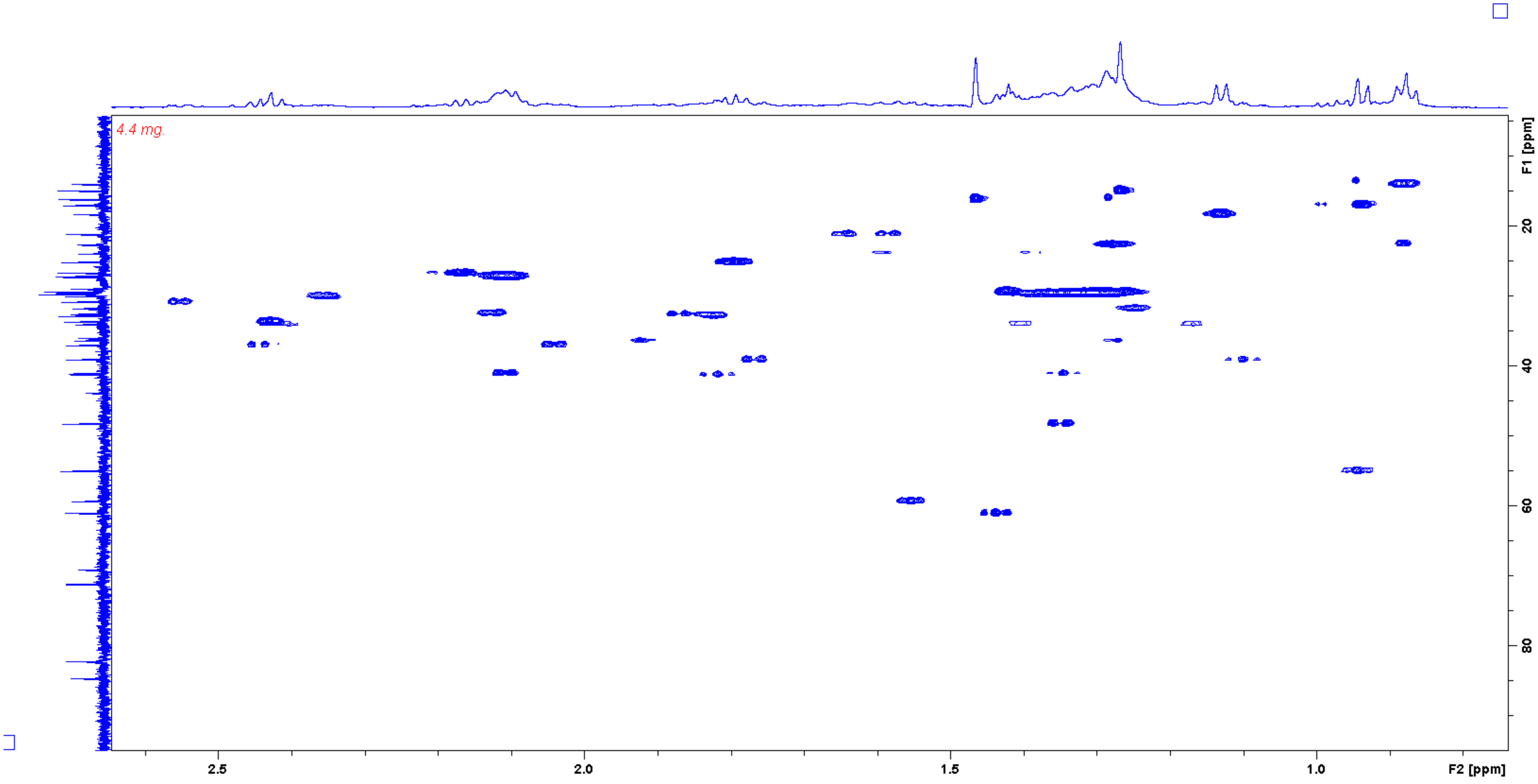

Figure S17. HMBC spectrum of compound **1** in C_5D_5N.

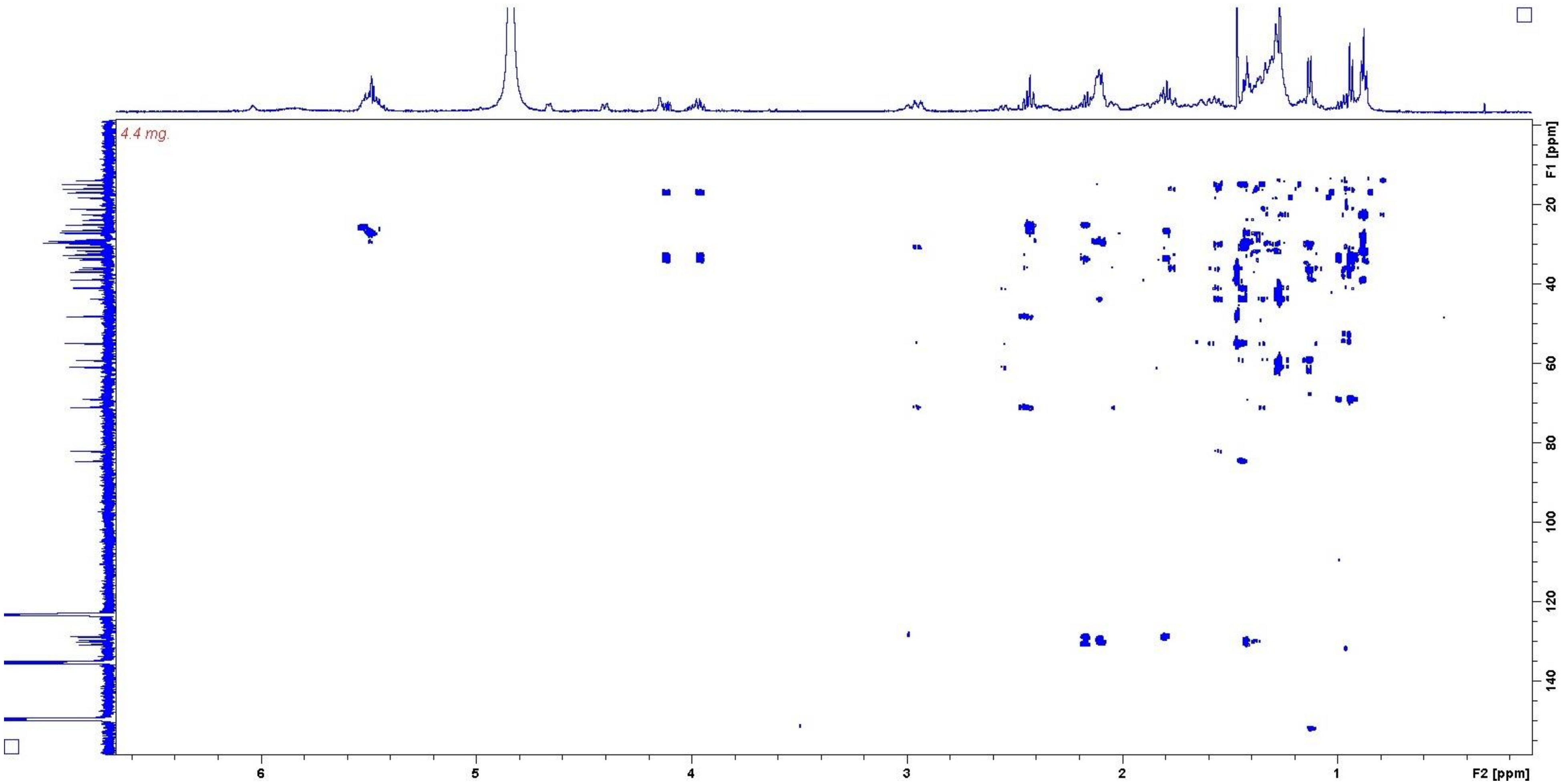

Figure S18. Enlarged HMBC spectrum of compound **1** in C_5D_5N.

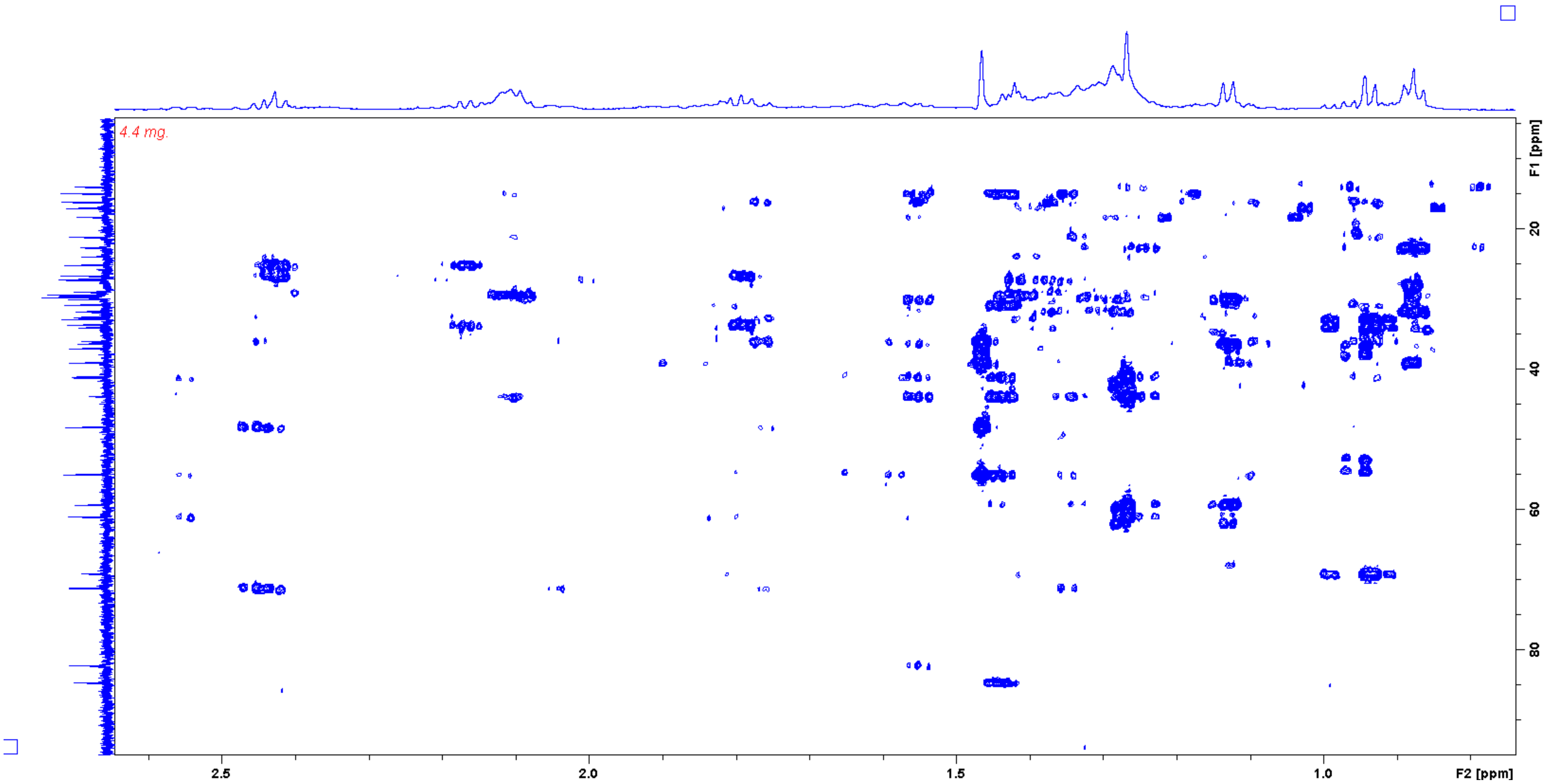

Figure S19. ROESY spectrum of compound **1** in C_5D_5N.

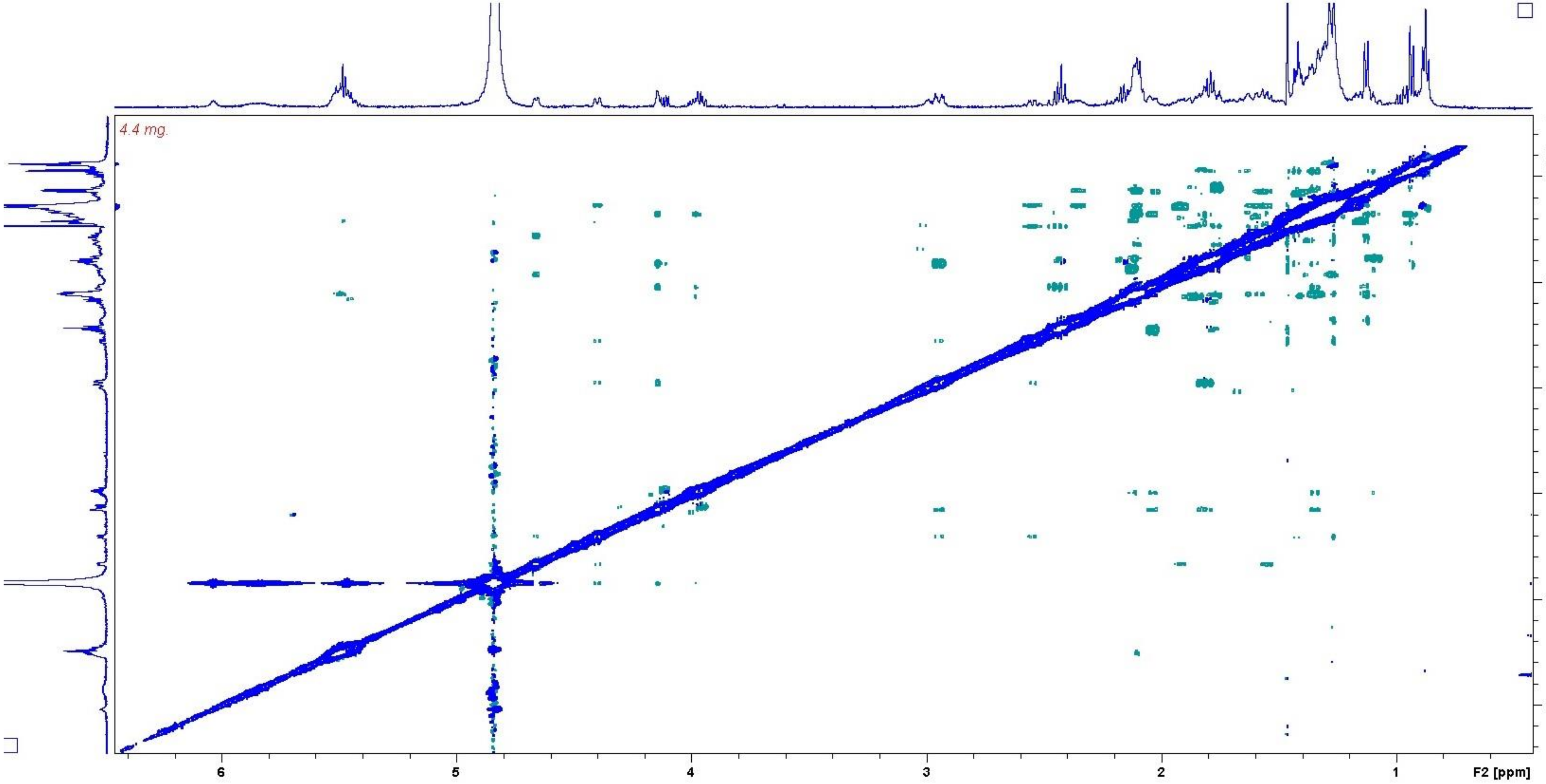

Figure S20. Enlarged ROESY spectrum of compound **1** in C_5D_5N.

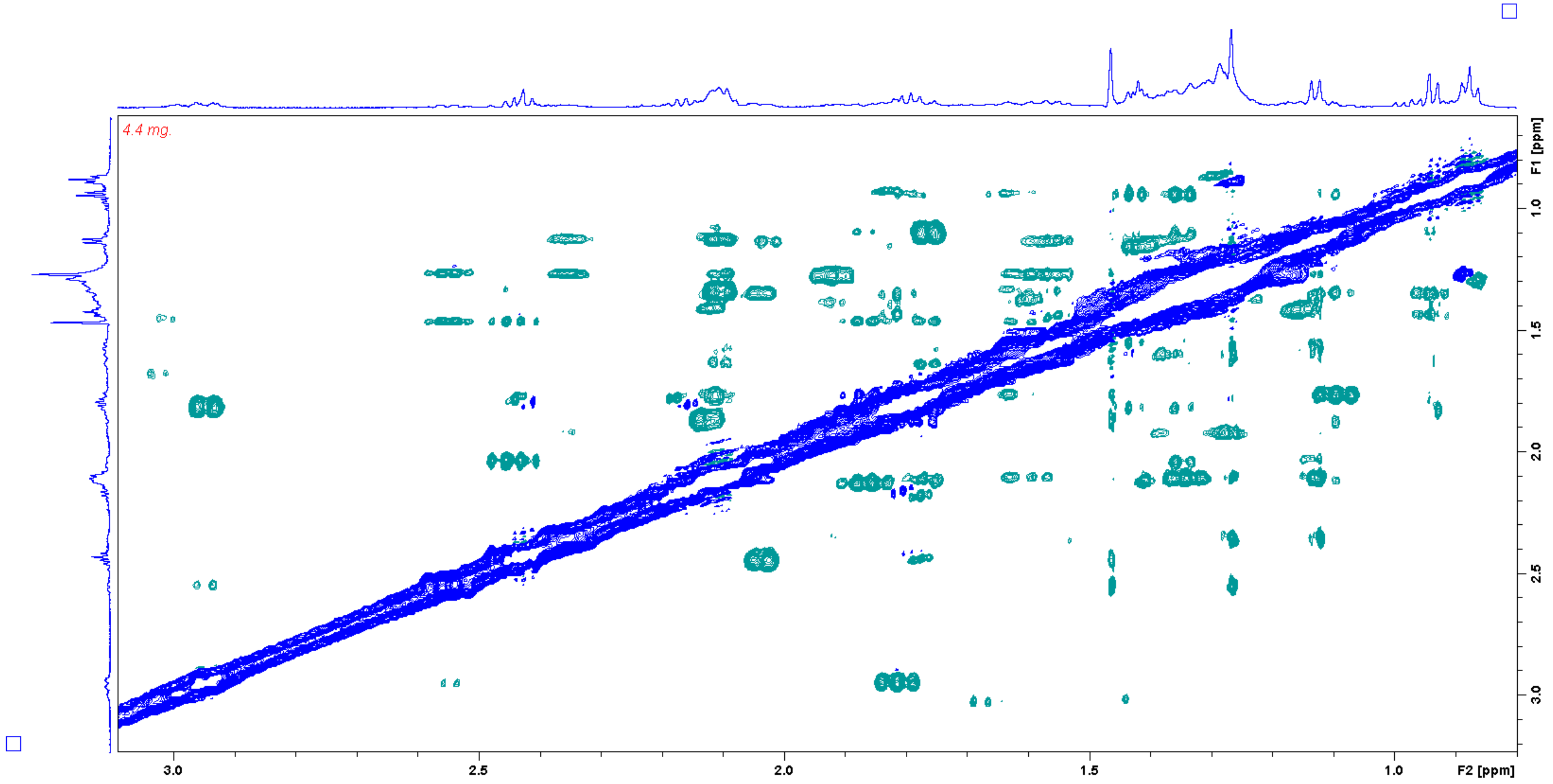

Figure S21. (+)-HRESIMS spectrum of compound **2**.

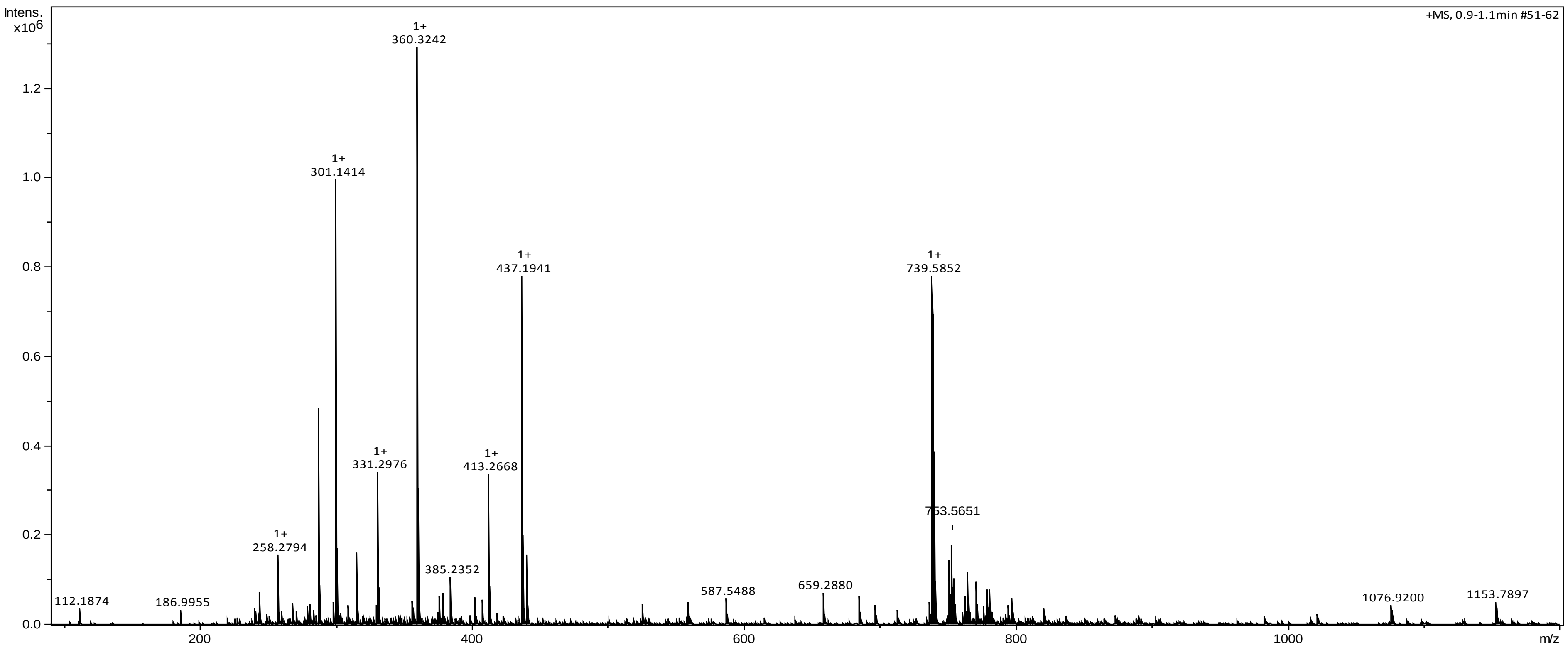

Figure S22. (–)-HRESIMS spectrum of compound **2**.

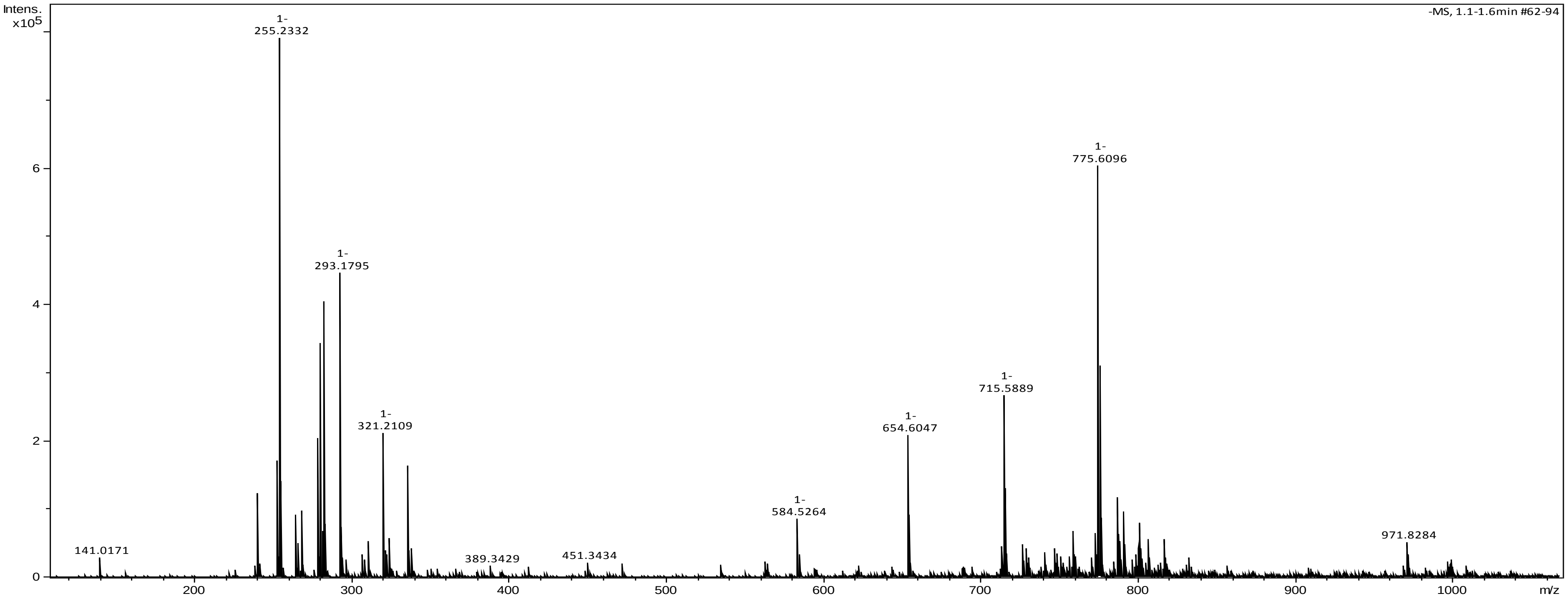

Figure S23. (–)-ESIMS/MS spectrum of compound **2**.

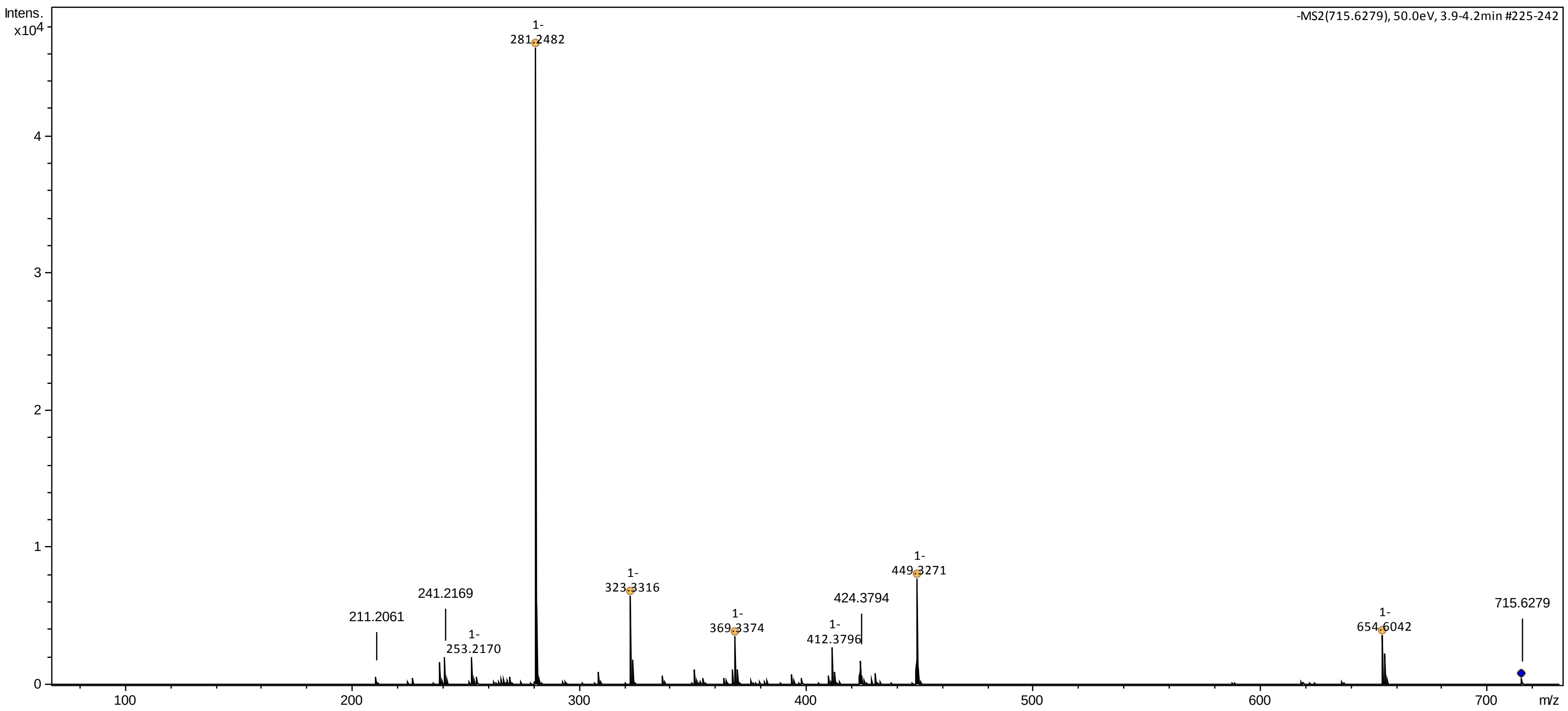

Figure S24. ^{1}H-NMR spectrum of compound **2** in C_5D_5N.

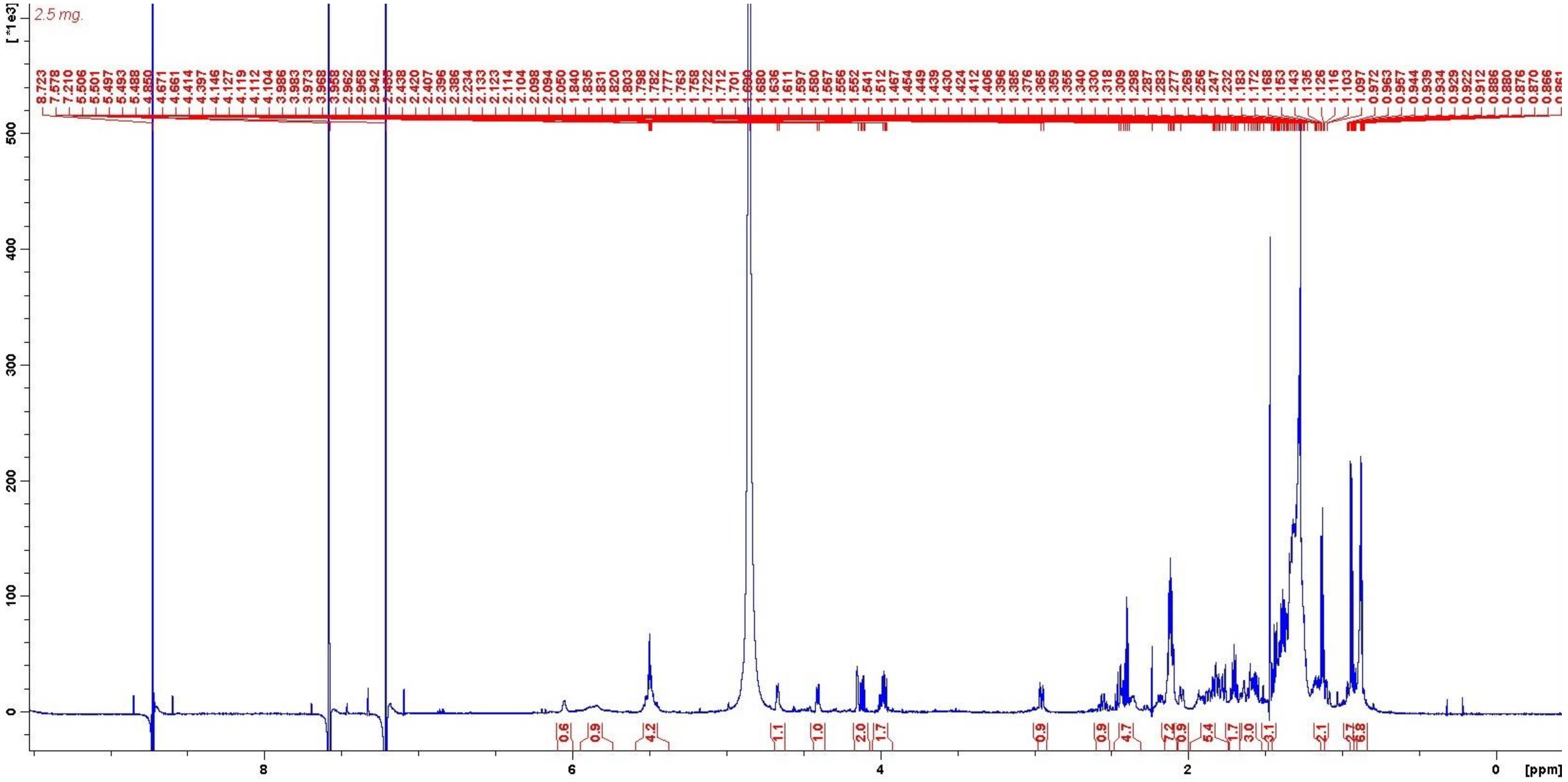

Figure S25. ^{13}C-NMR spectrum of compound **2** in C_5D_5N.

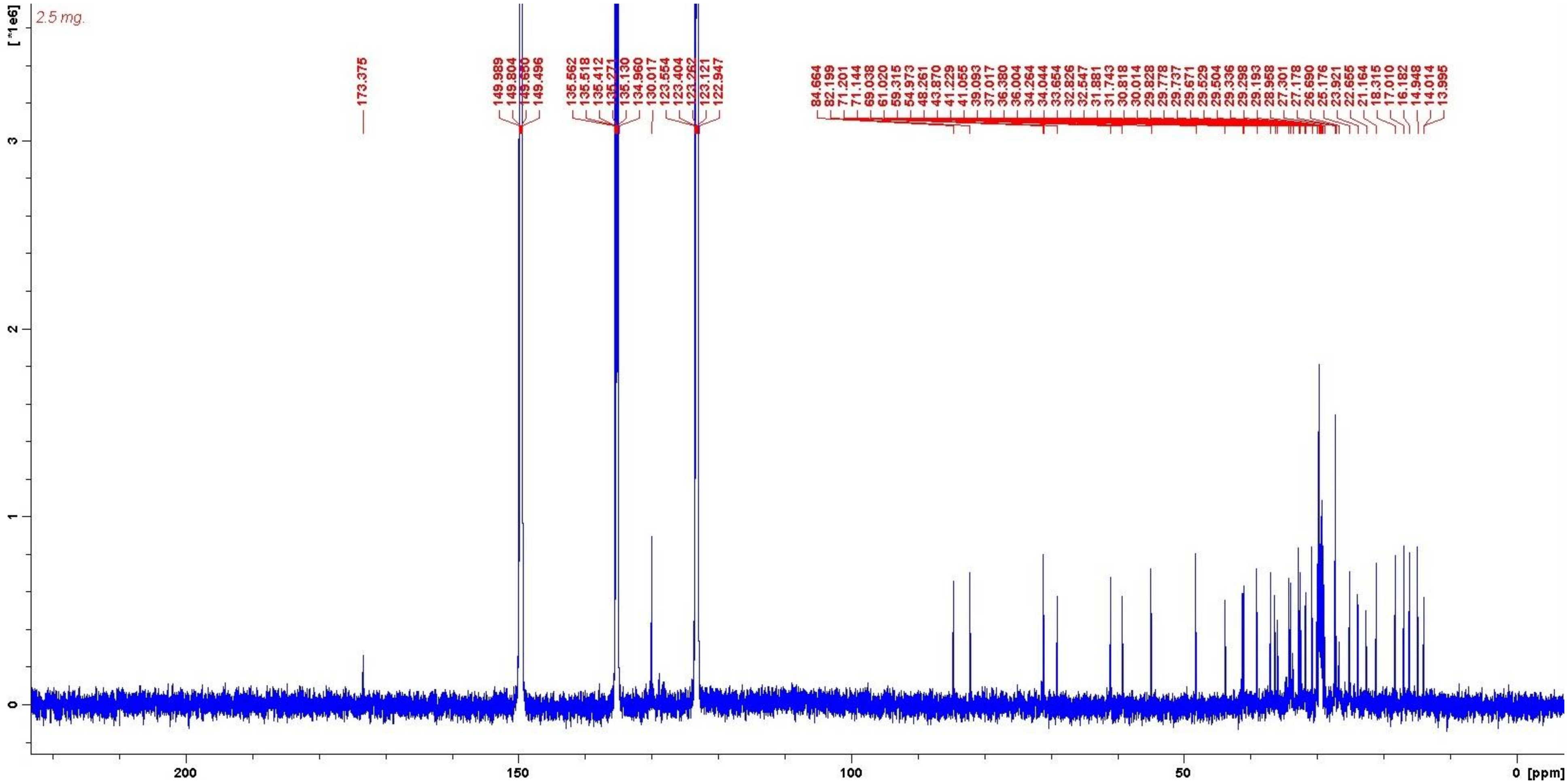

Figure S26. ^{1}H-^{1}H-COSY spectrum of compound **2** in C_5D_5N.

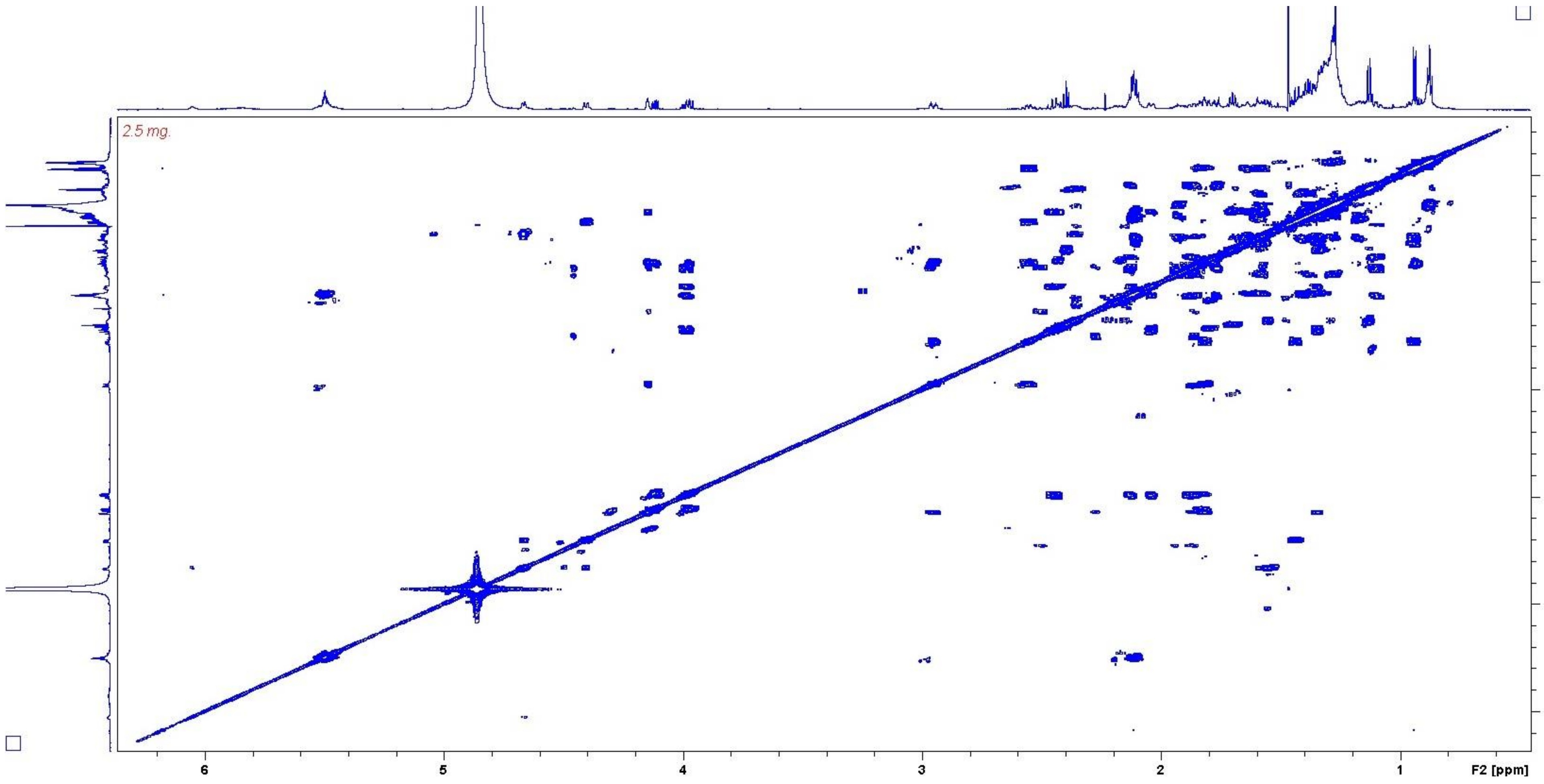

Figure S27. HSQC spectrum of compound **2** in C_5D_5N.

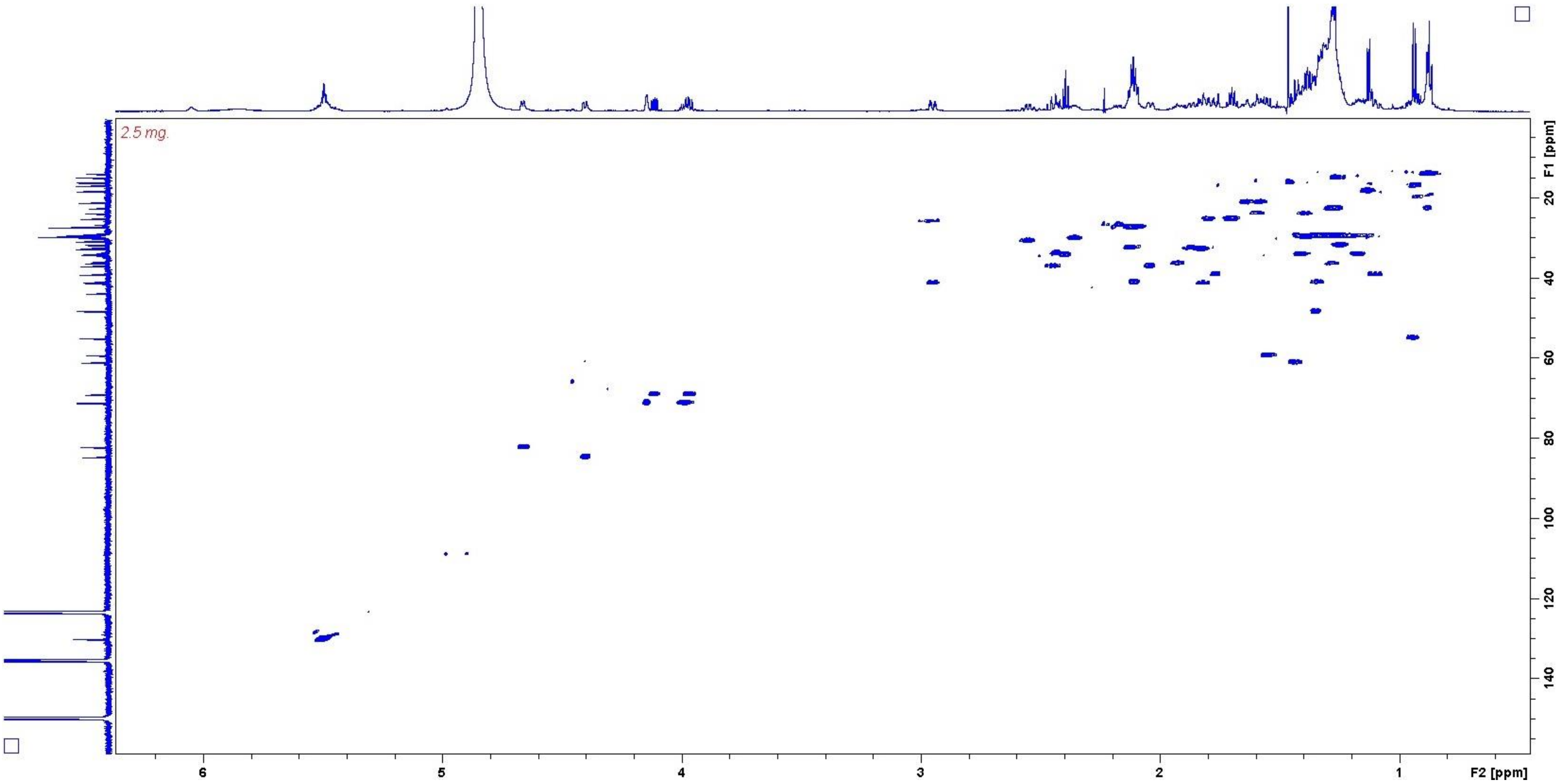

Figure S28. HMBC spectrum of compound **2** in C_5D_5N.

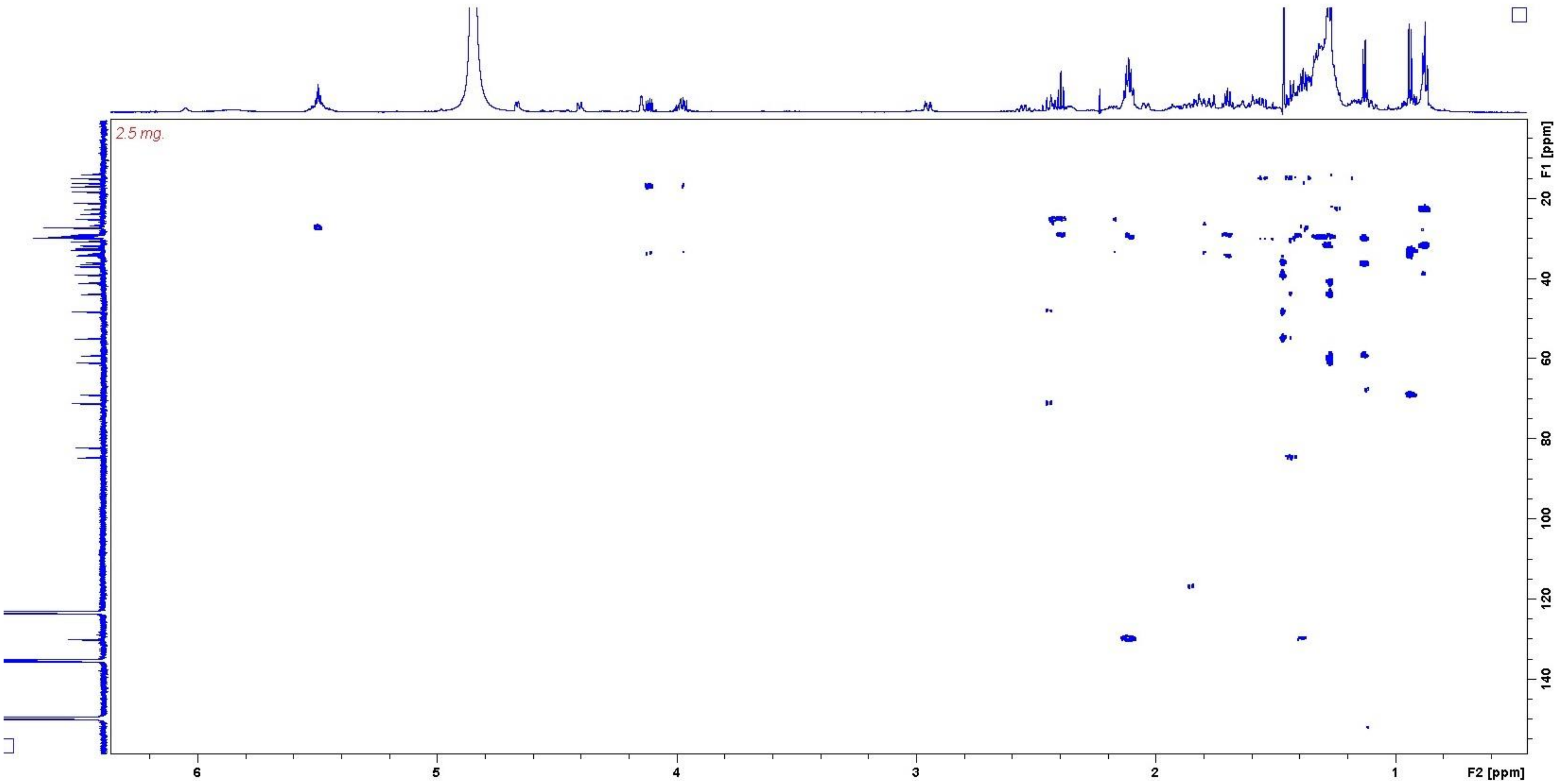

Figure S29. ROESY spectrum of compound **2** in C_5D_5N.

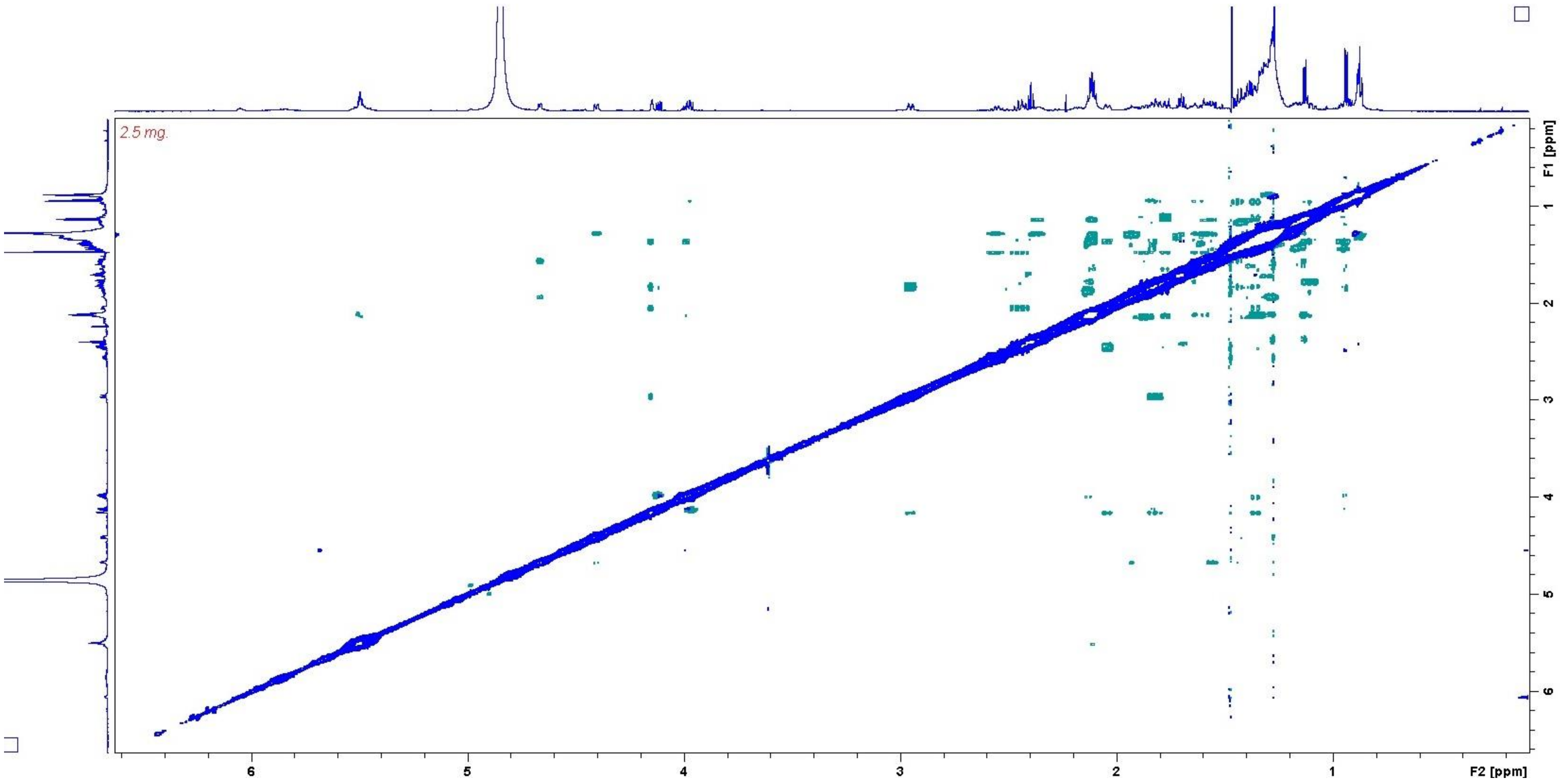

Figure S30. (+)-HRESIMS spectrum of compound **3**.

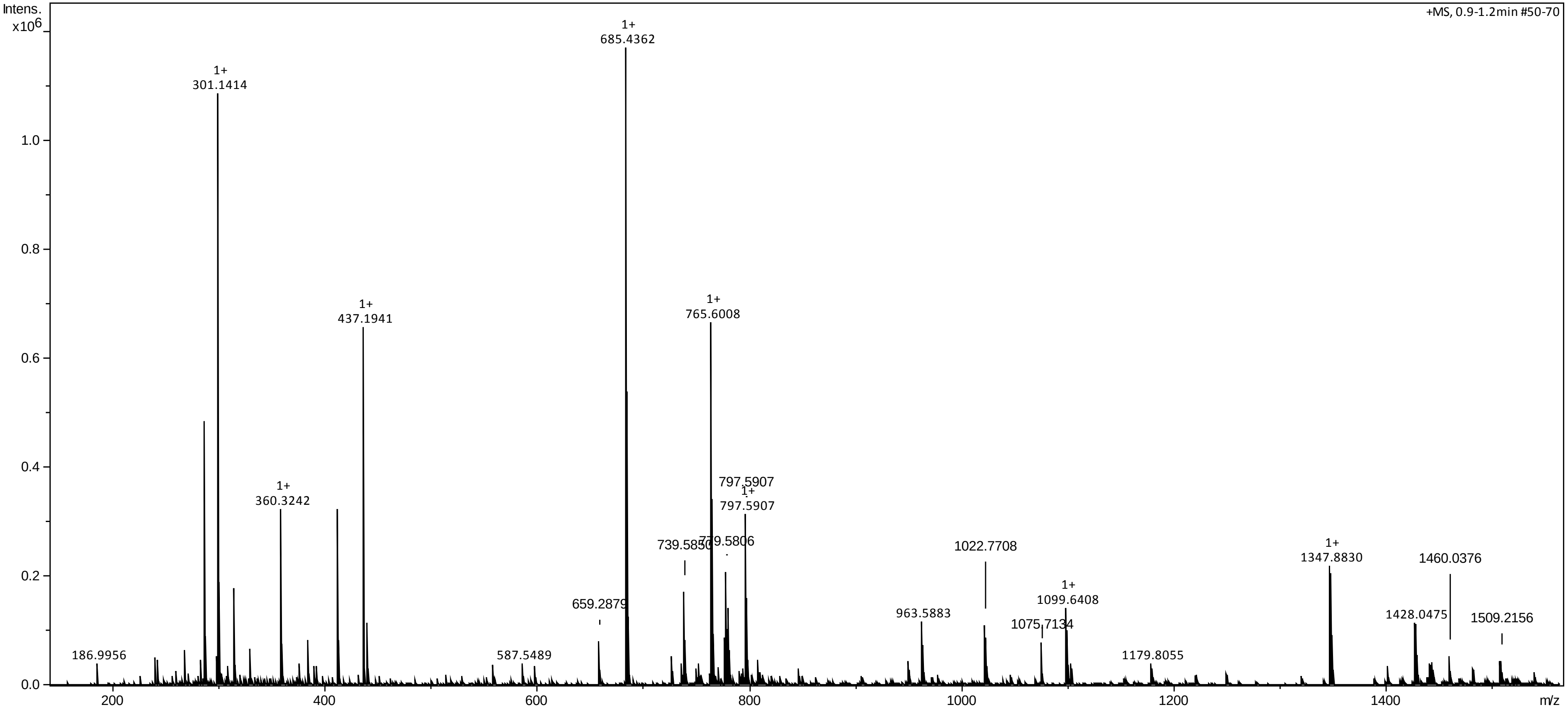

Figure S31. (–)-HRESIMS spectrum of compound **3**.

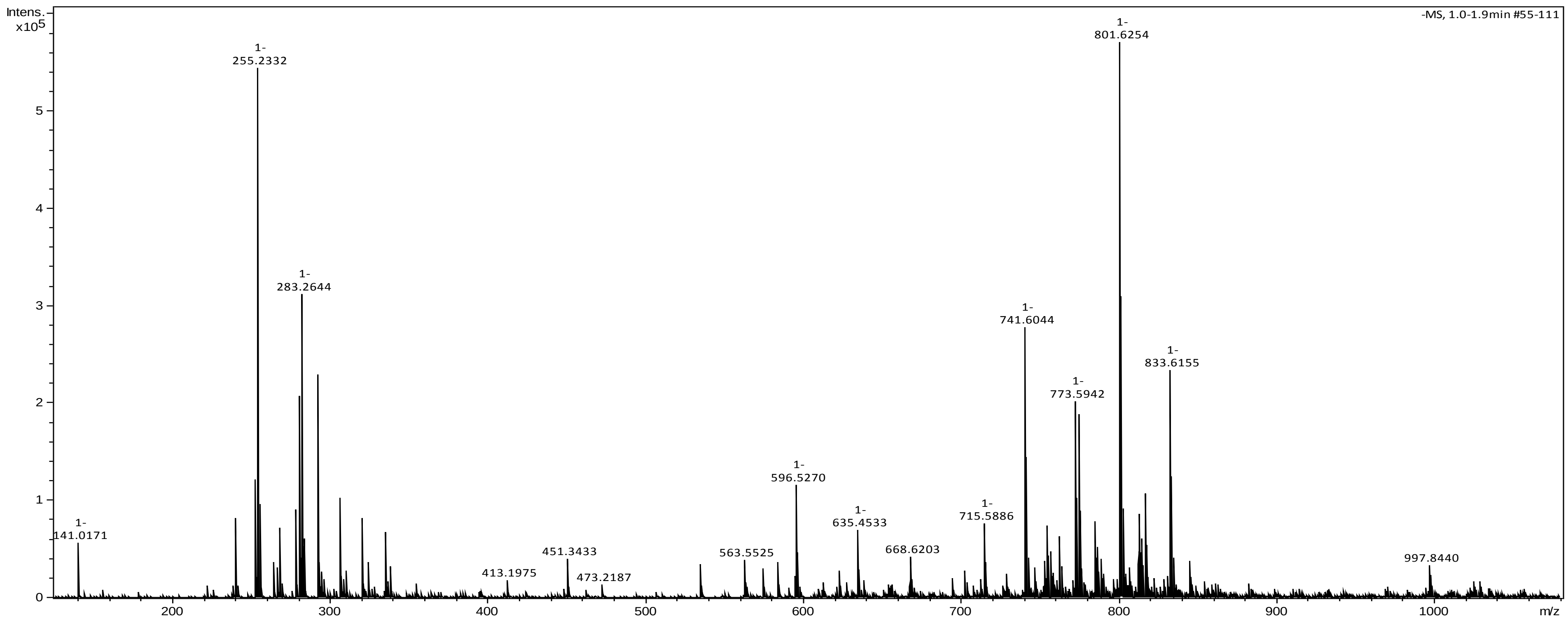

Figure S32. (–)-ESIMS/MS spectrum of compound **3**.

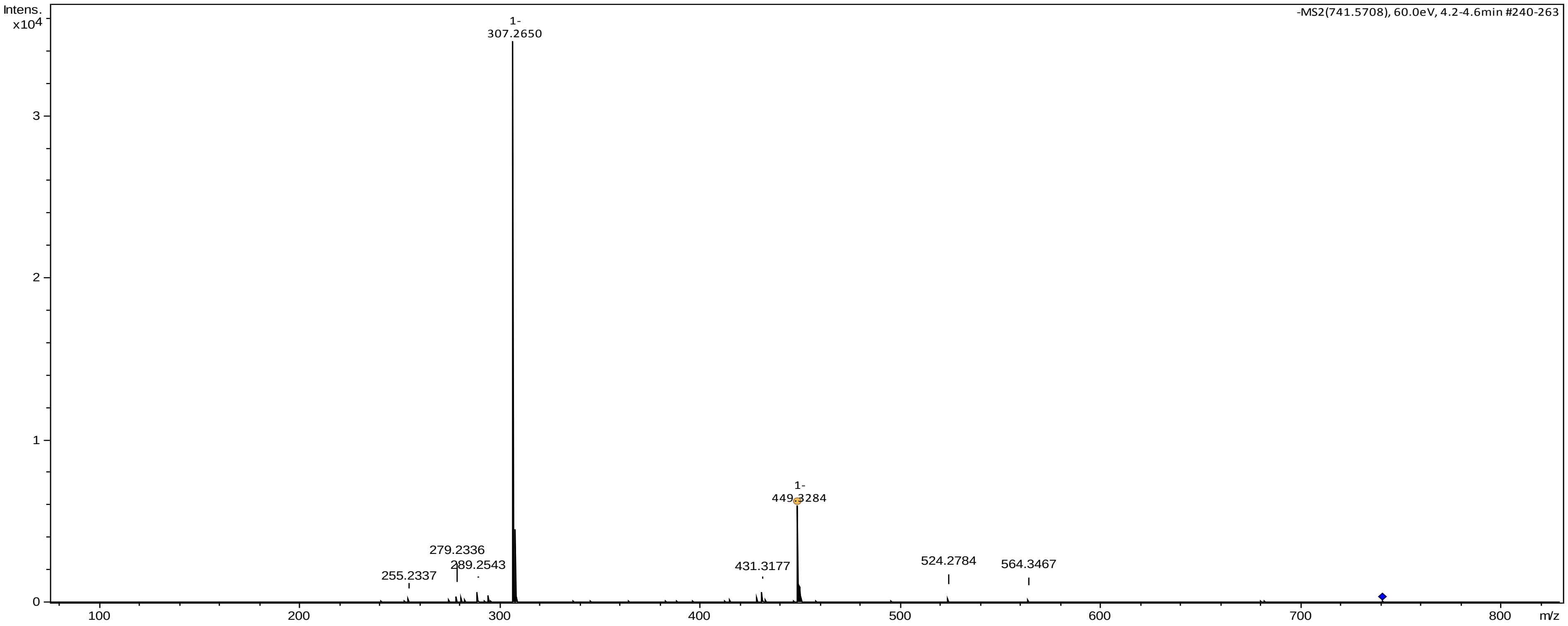

Figure S33. ^{1}H-NMR spectrum of compound **3** in C_5D_5N.

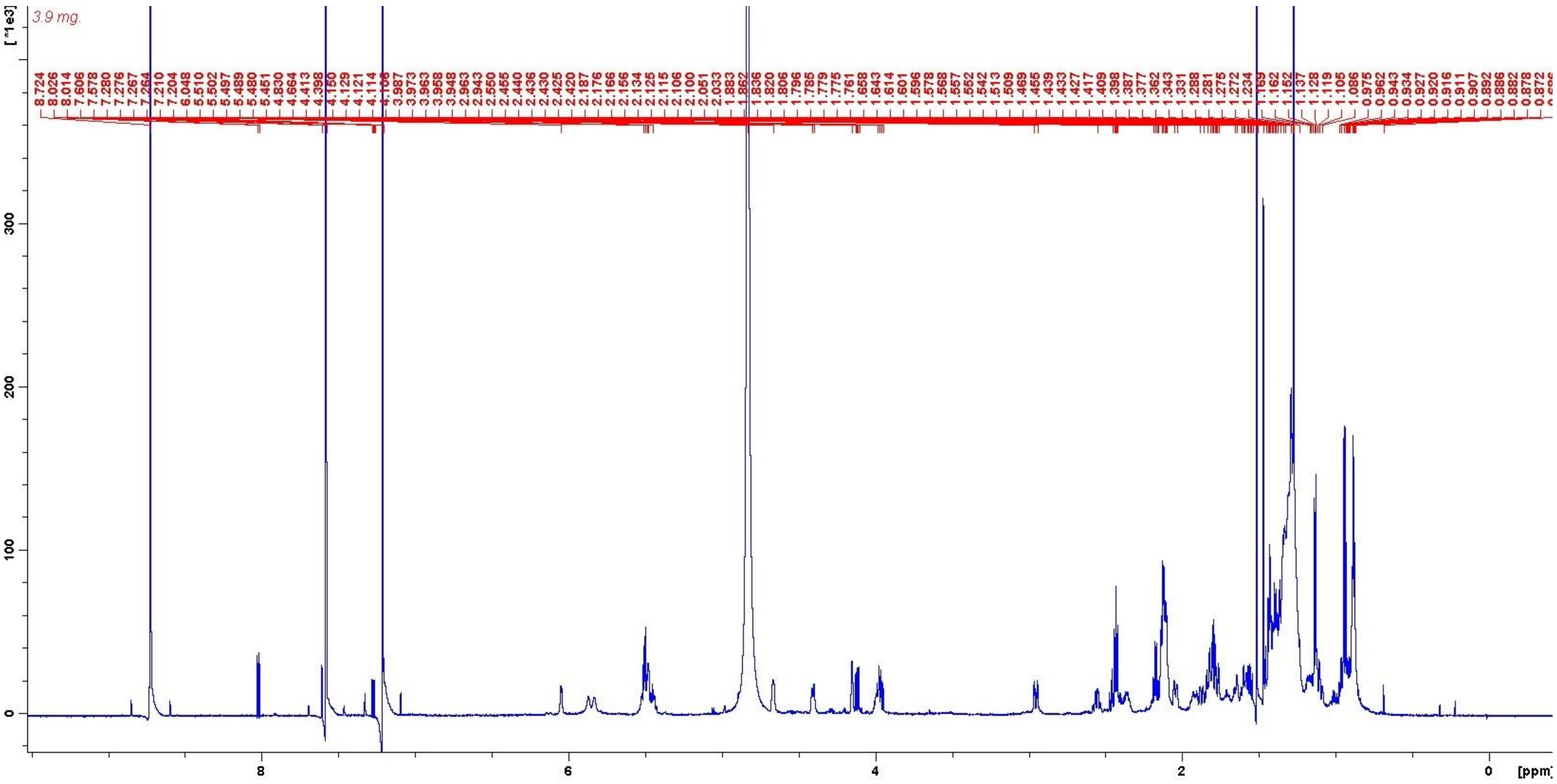

Figure S34. ^{13}C-NMR spectrum of compound **3** in C_5D_5N.

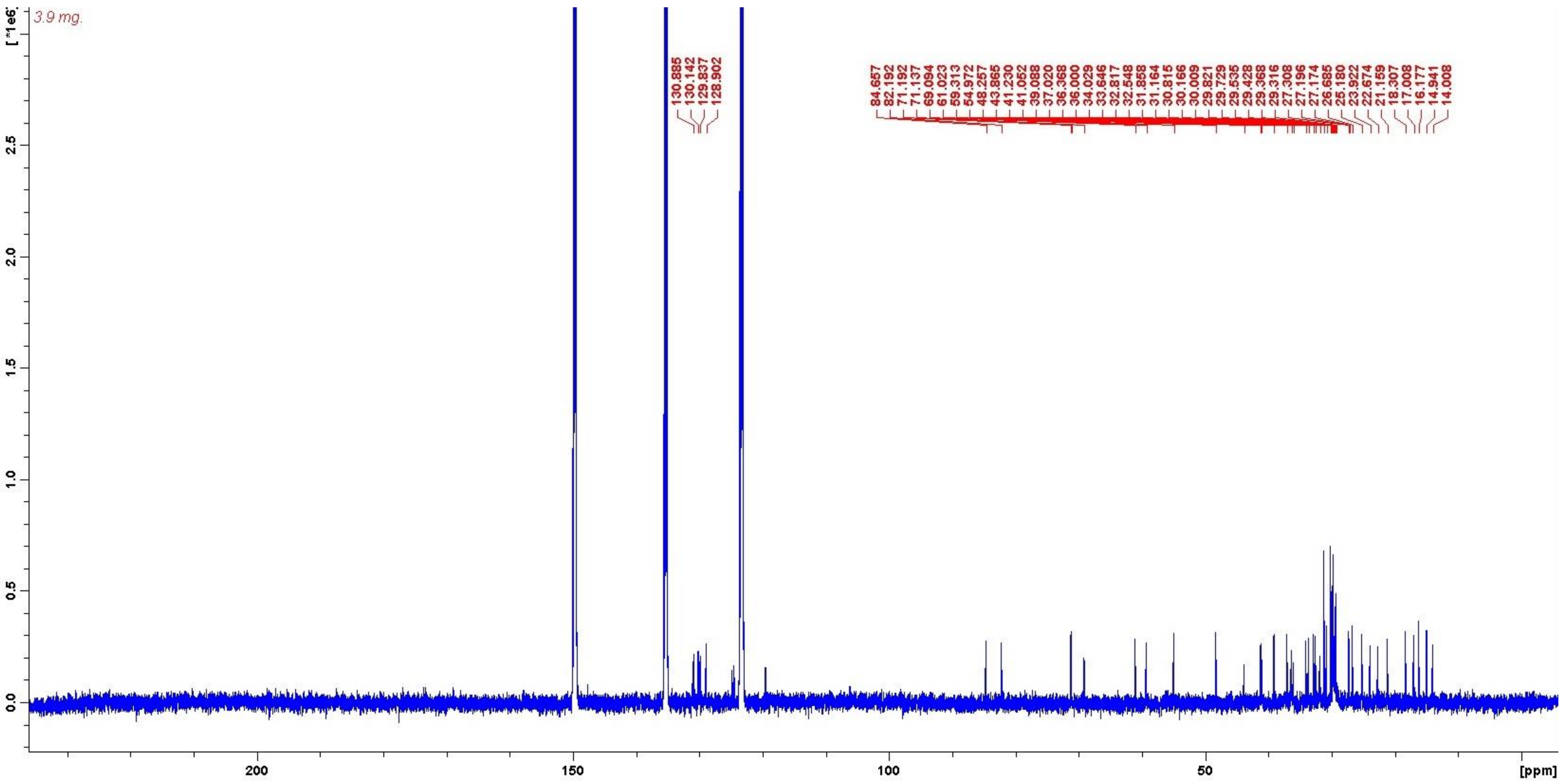

Figure S35. ^1H-^1H-COSY spectrum of compound **3** in C_5D_5N.

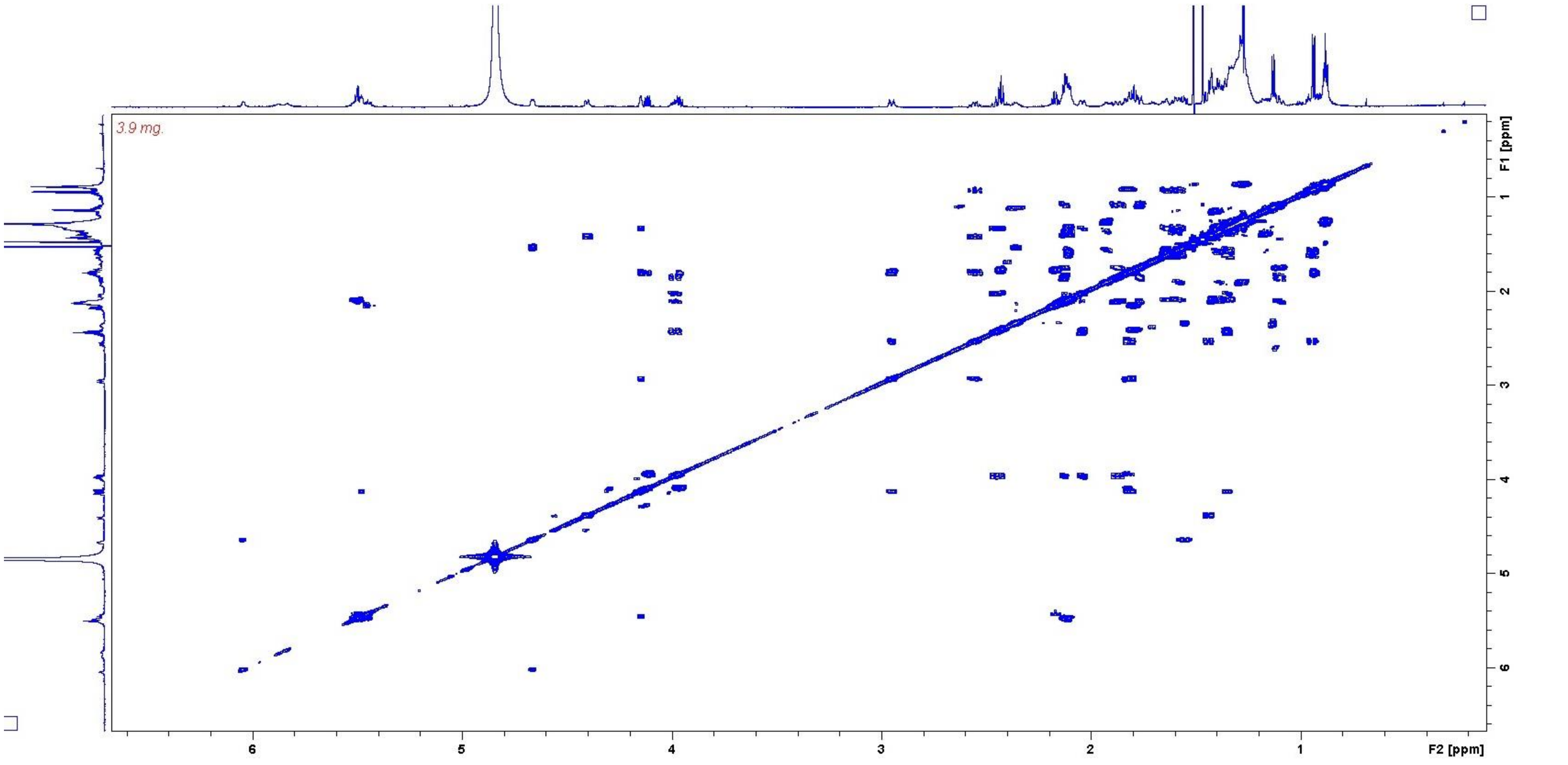

Figure S36. HSQC spectrum of compound **3** in C_5D_5N.

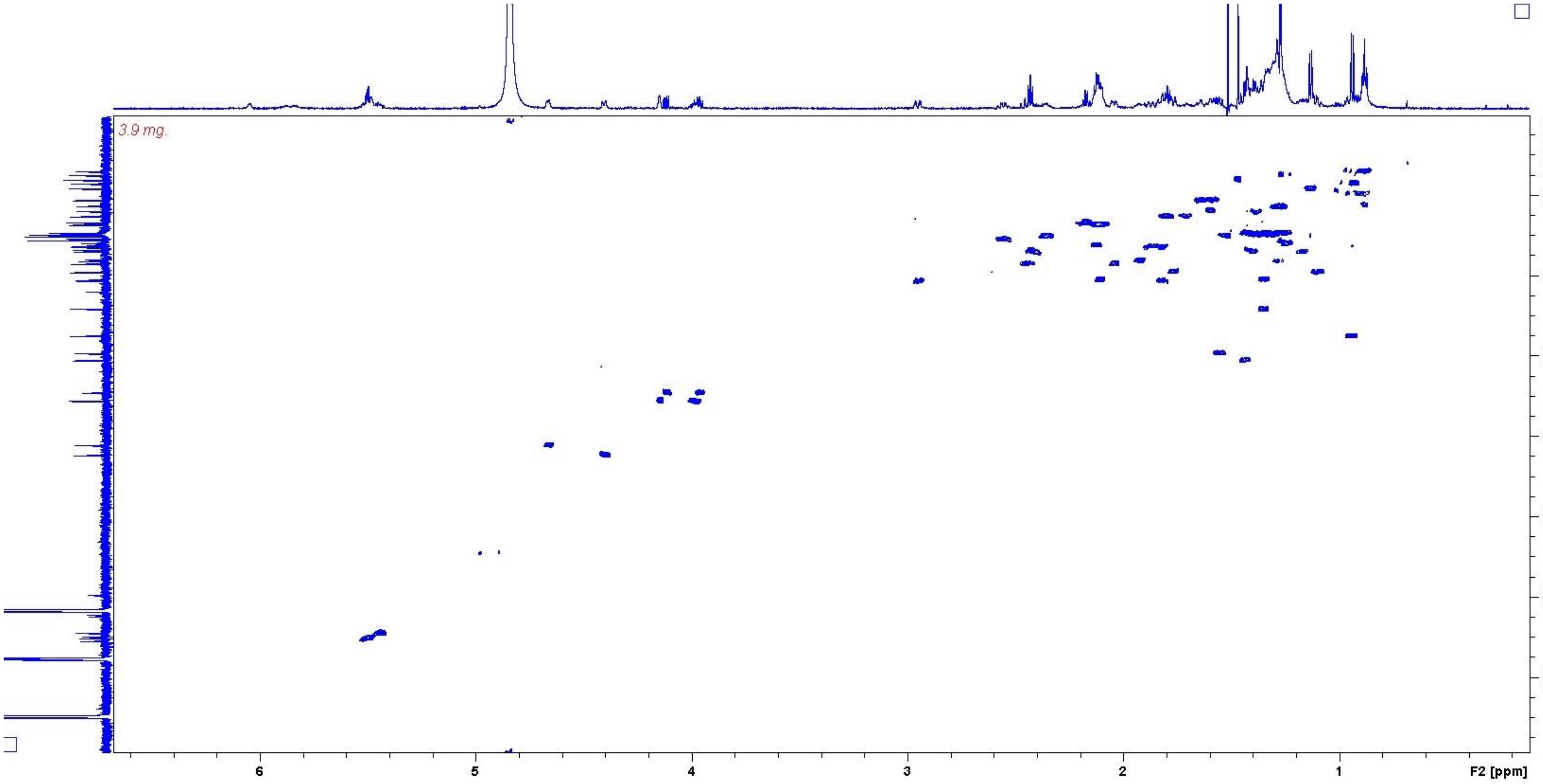

Figure S37. HMBC spectrum of compound **3** in C_5D_5N.

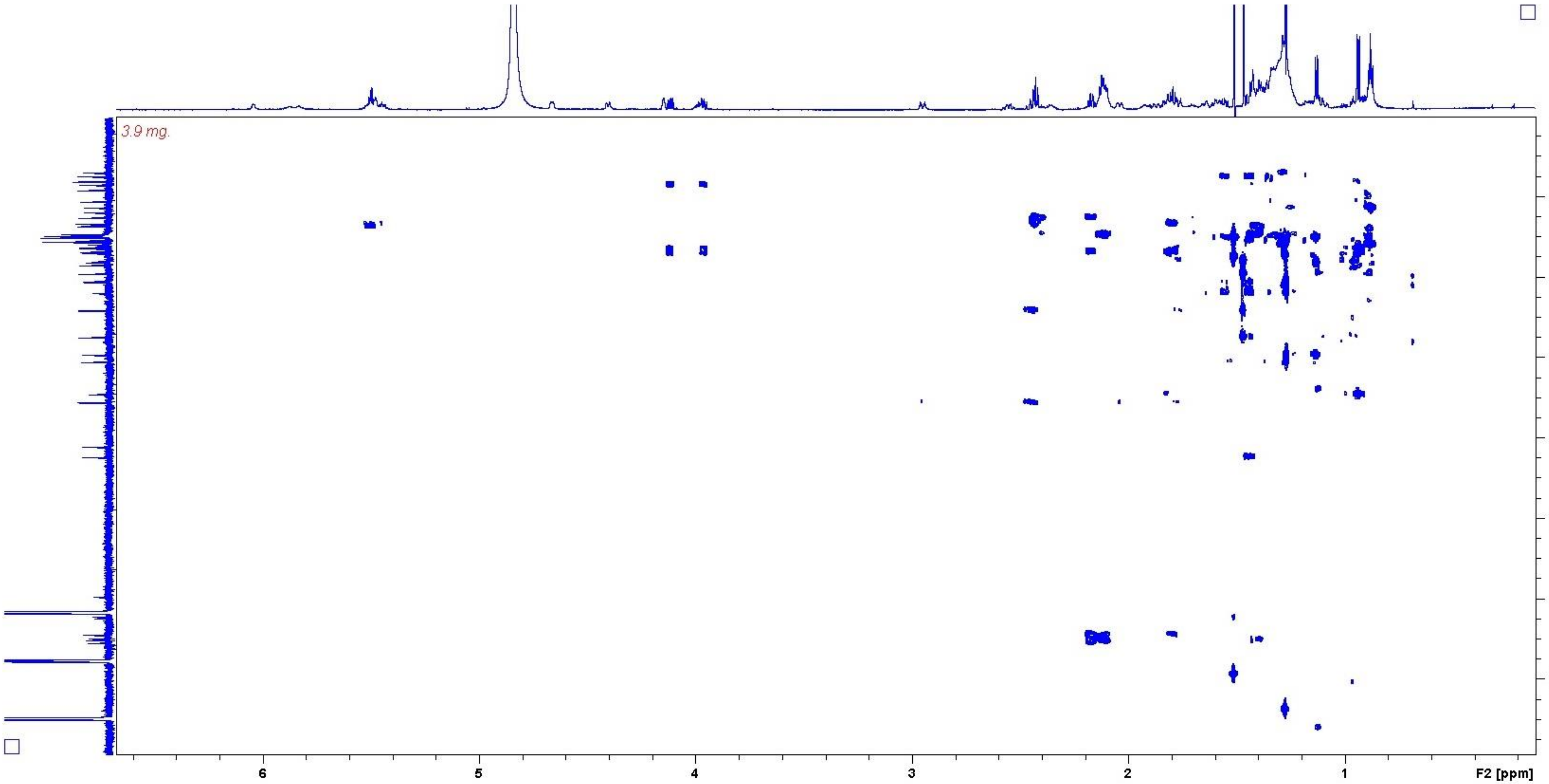

Figure S38. ROESY spectrum of compound **3** in C_5D_5N.

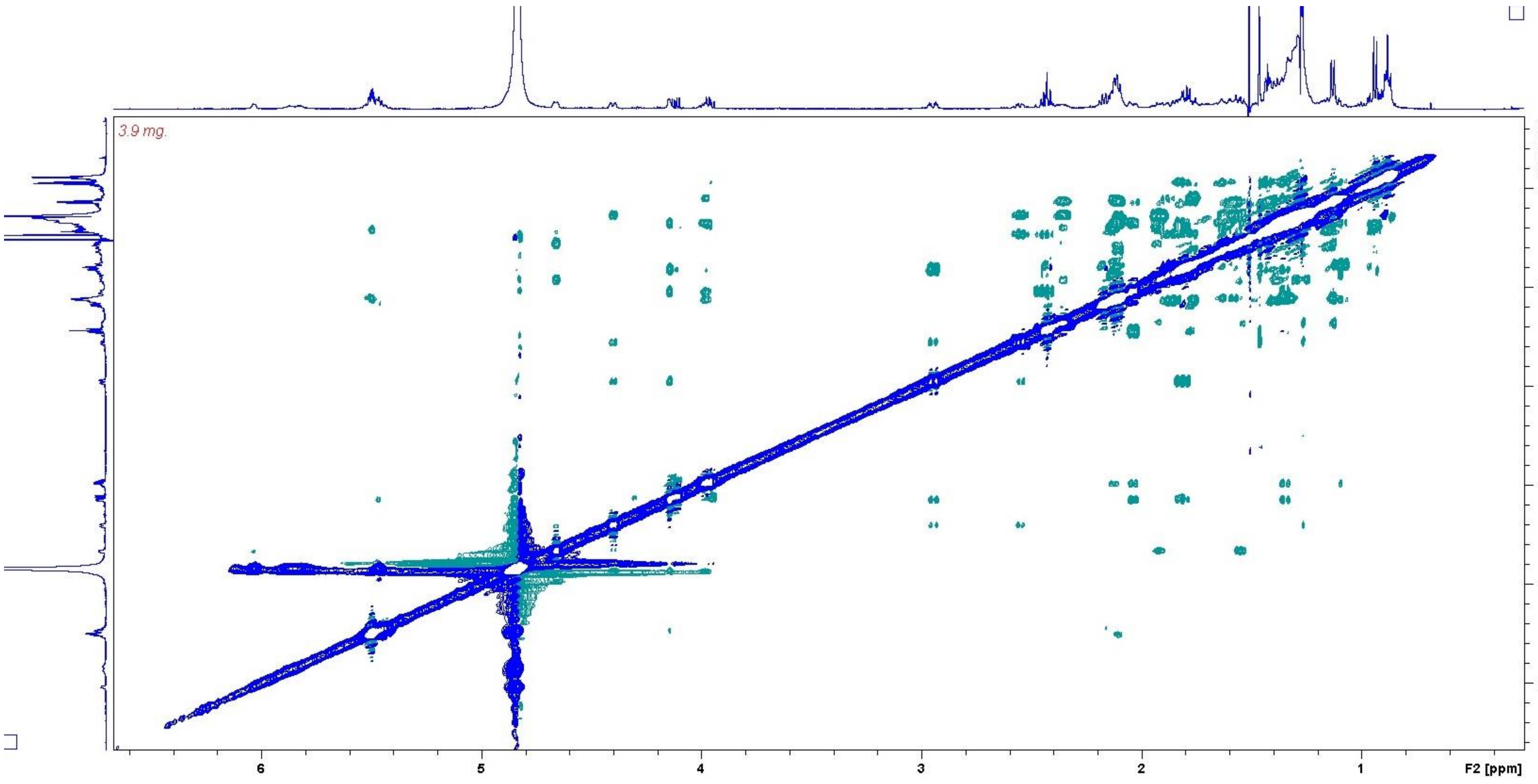

Figure S39. (+)-HRESIMS spectrum of compound **4**.

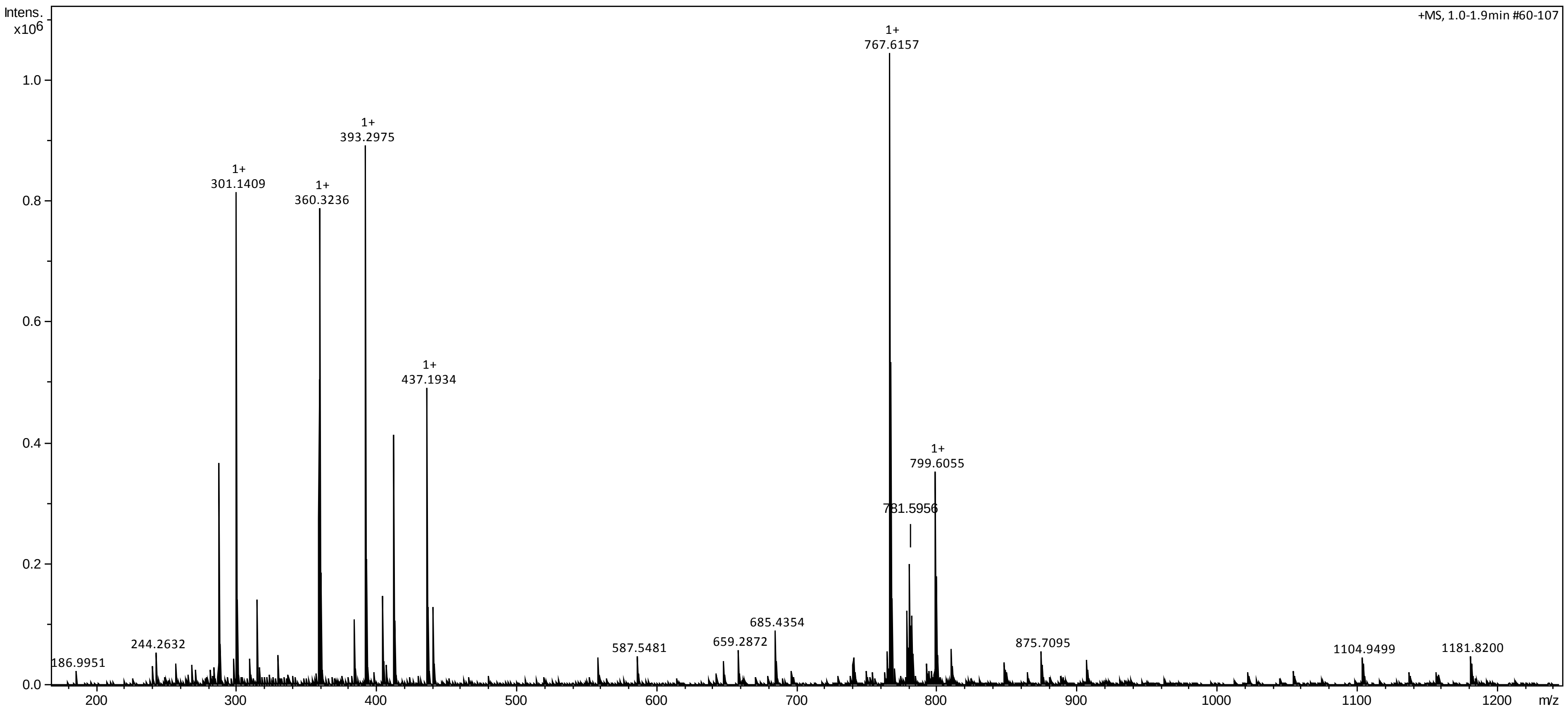

Figure S40. (–)-HRESIMS spectrum of compound **4**.

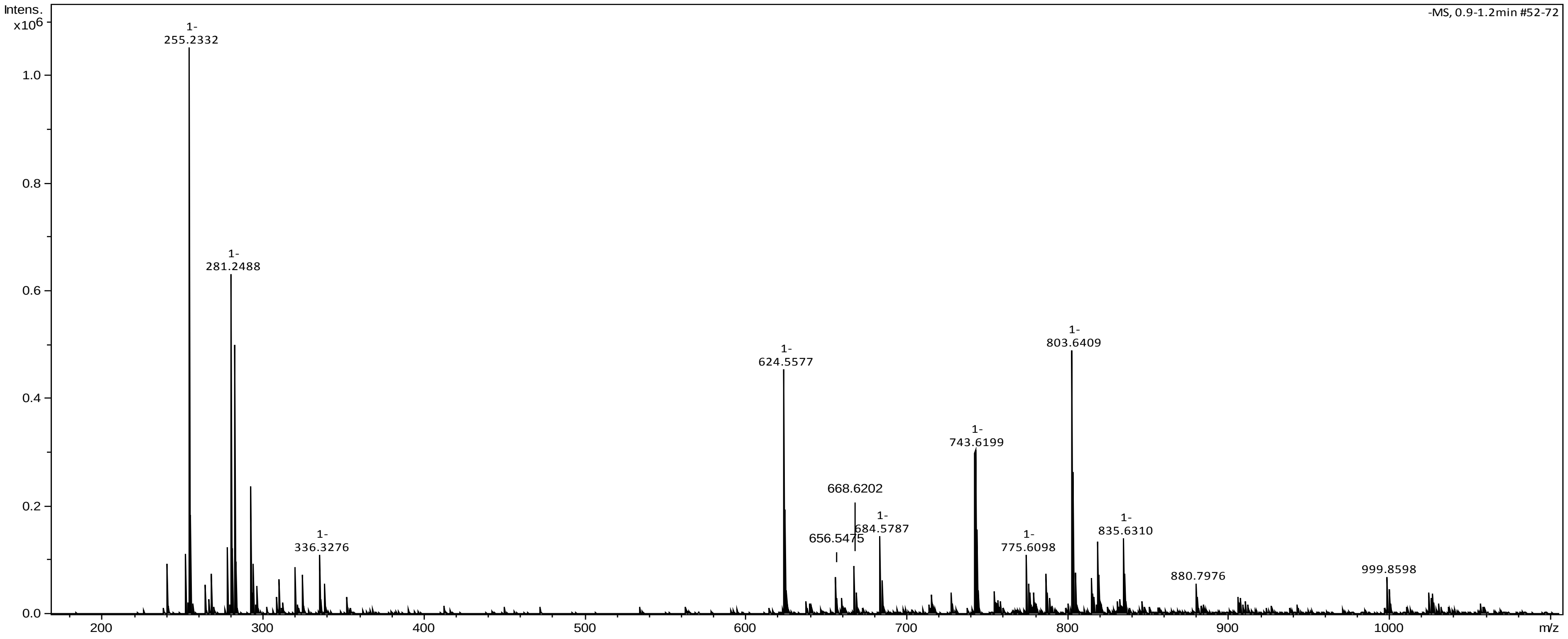

Figure S41. (–)-ESIMS/MS spectrum of compound **4**.

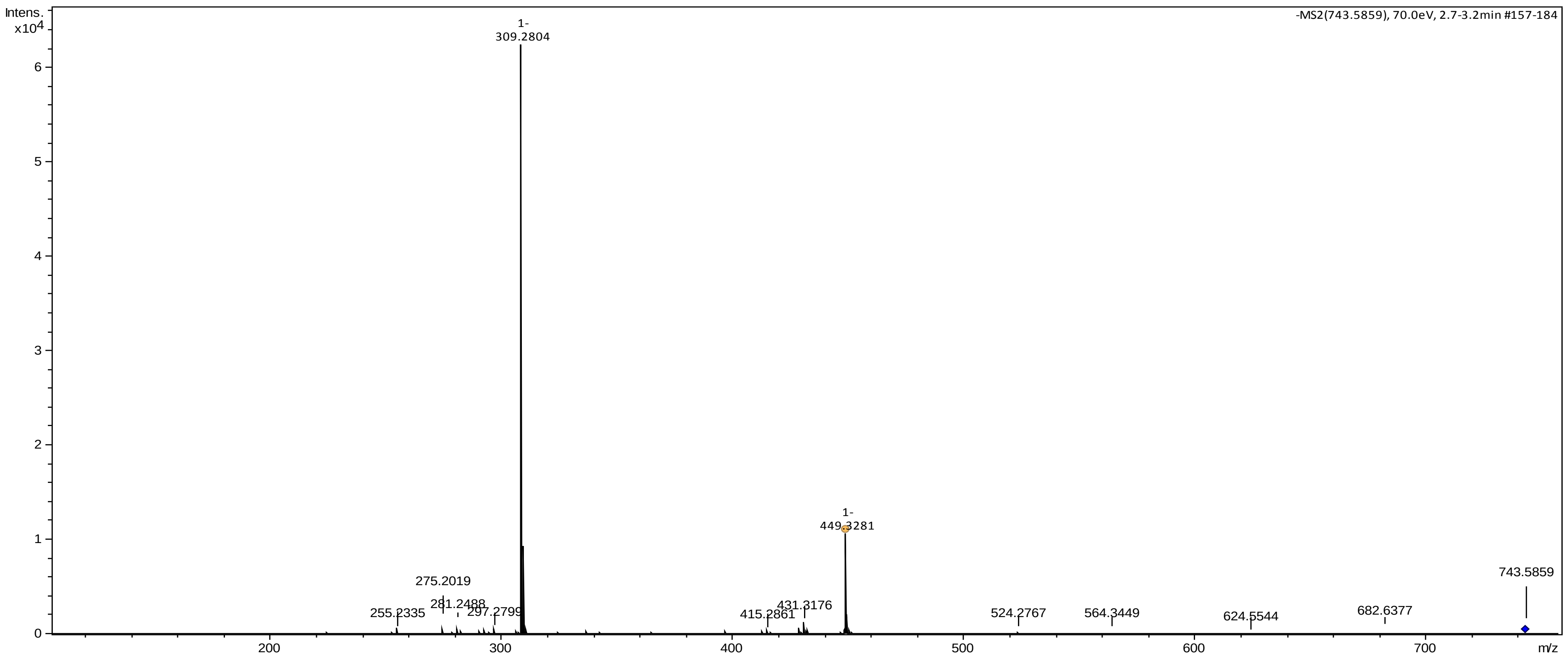

Figure S42. ^{1}H-NMR spectrum of compound **4** in C_5D_5N.

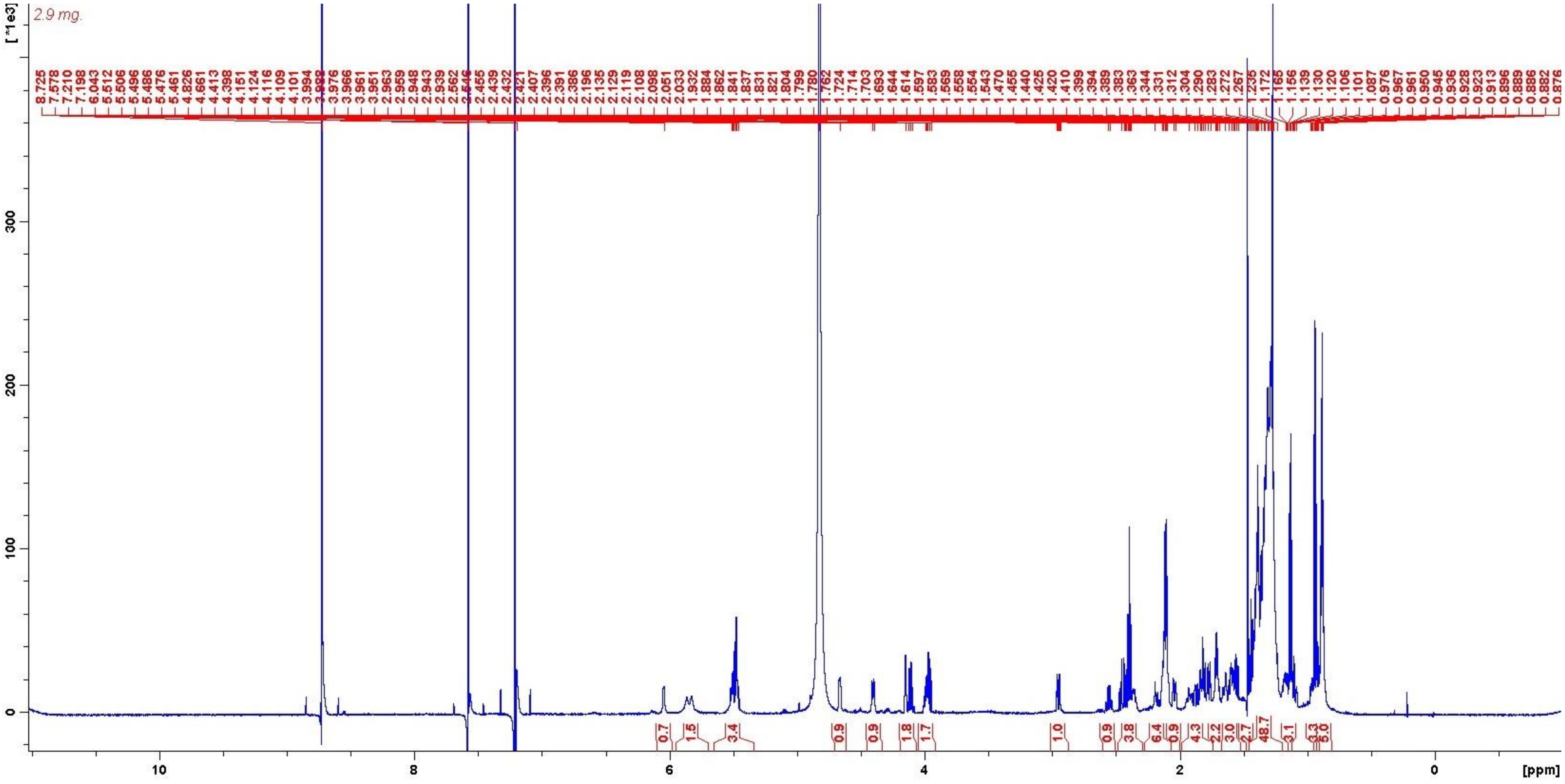

Figure S43. ^{13}C-NMR spectrum of compound **4** in C_5D_5N.

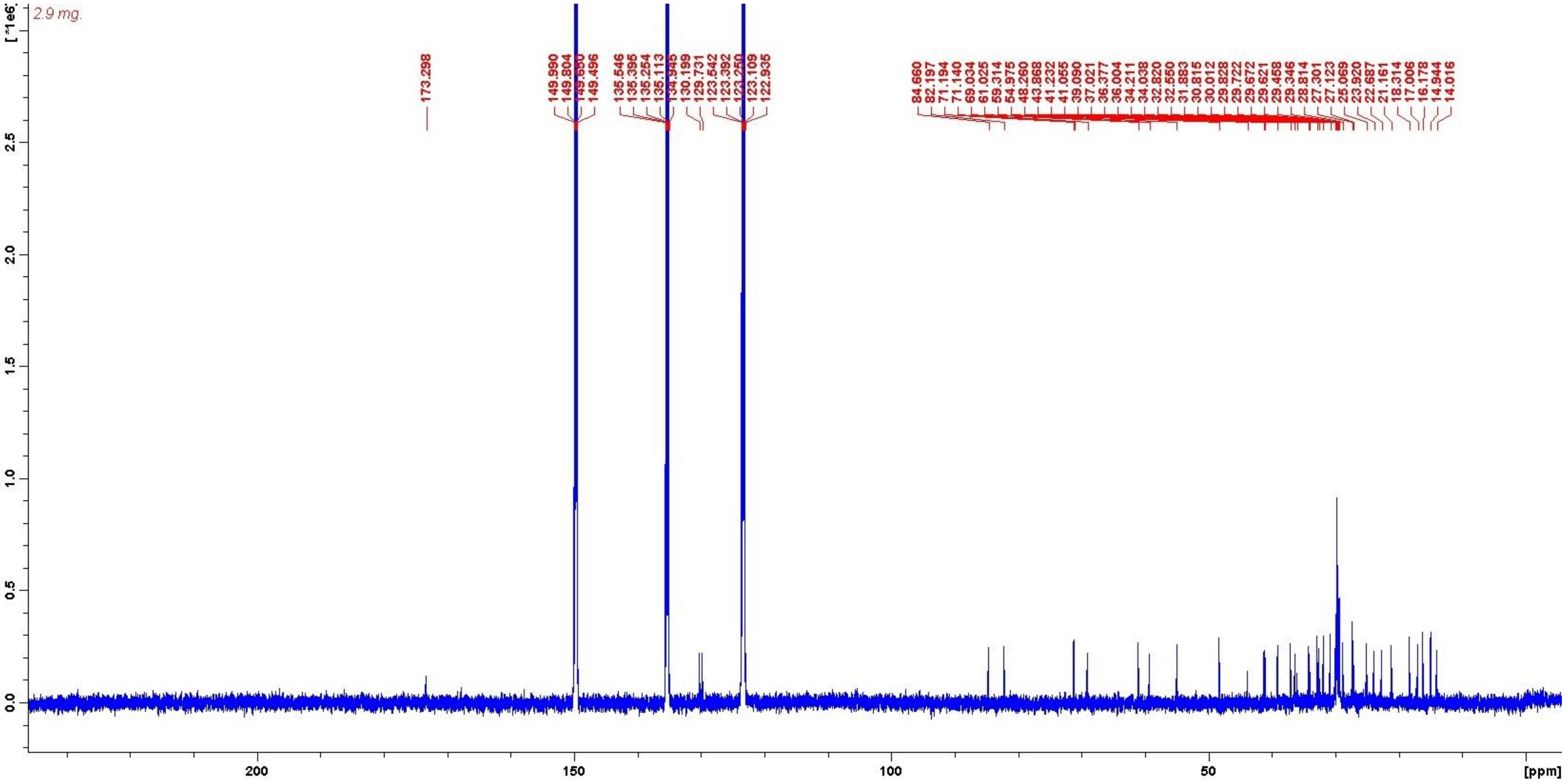

Figure S44. ^{1}H-^{1}H-COSY spectrum of compound **4** in C_5D_5N.

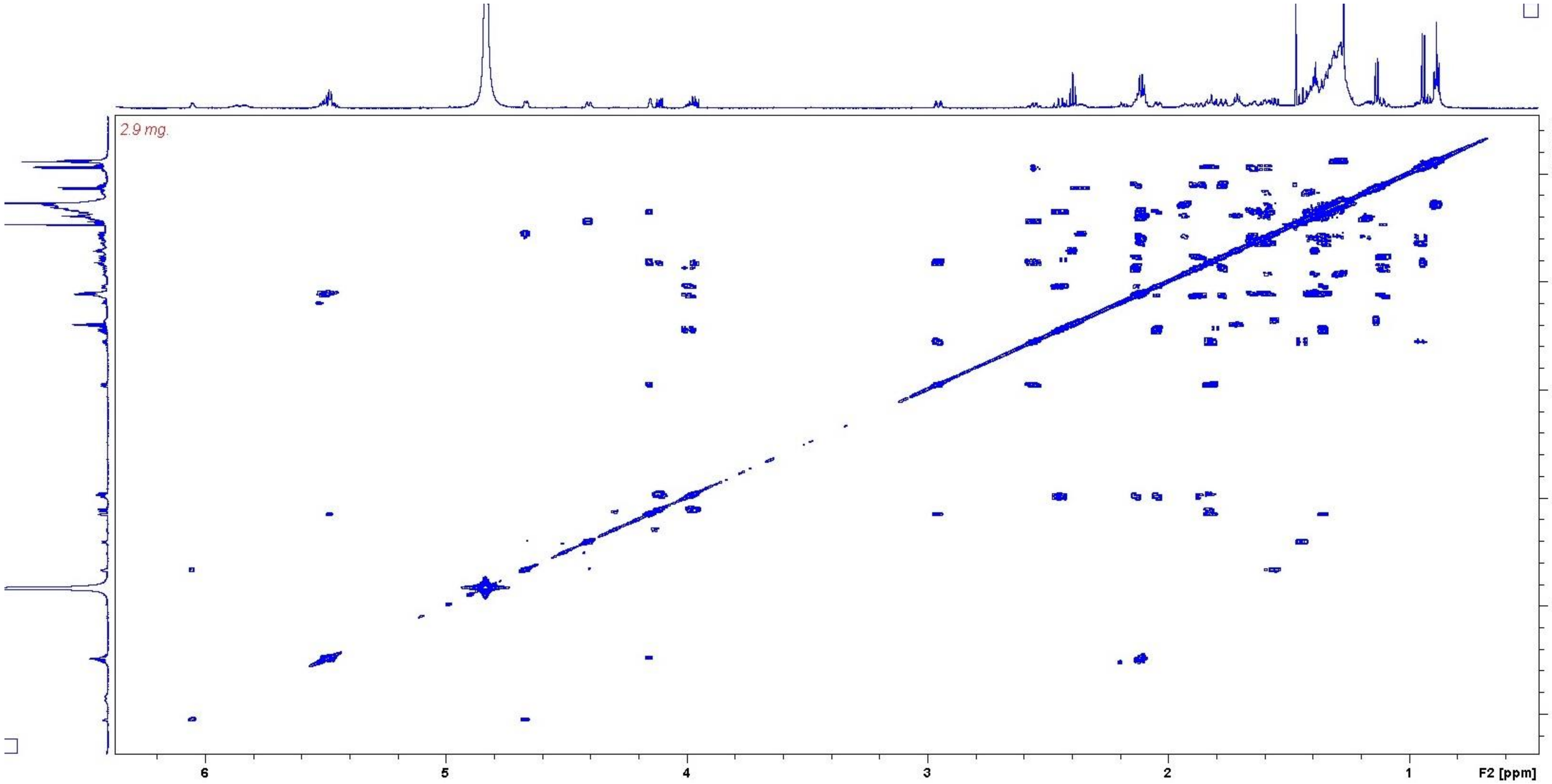

Figure S45. HSQC spectrum of compound **4** in C_5D_5N.

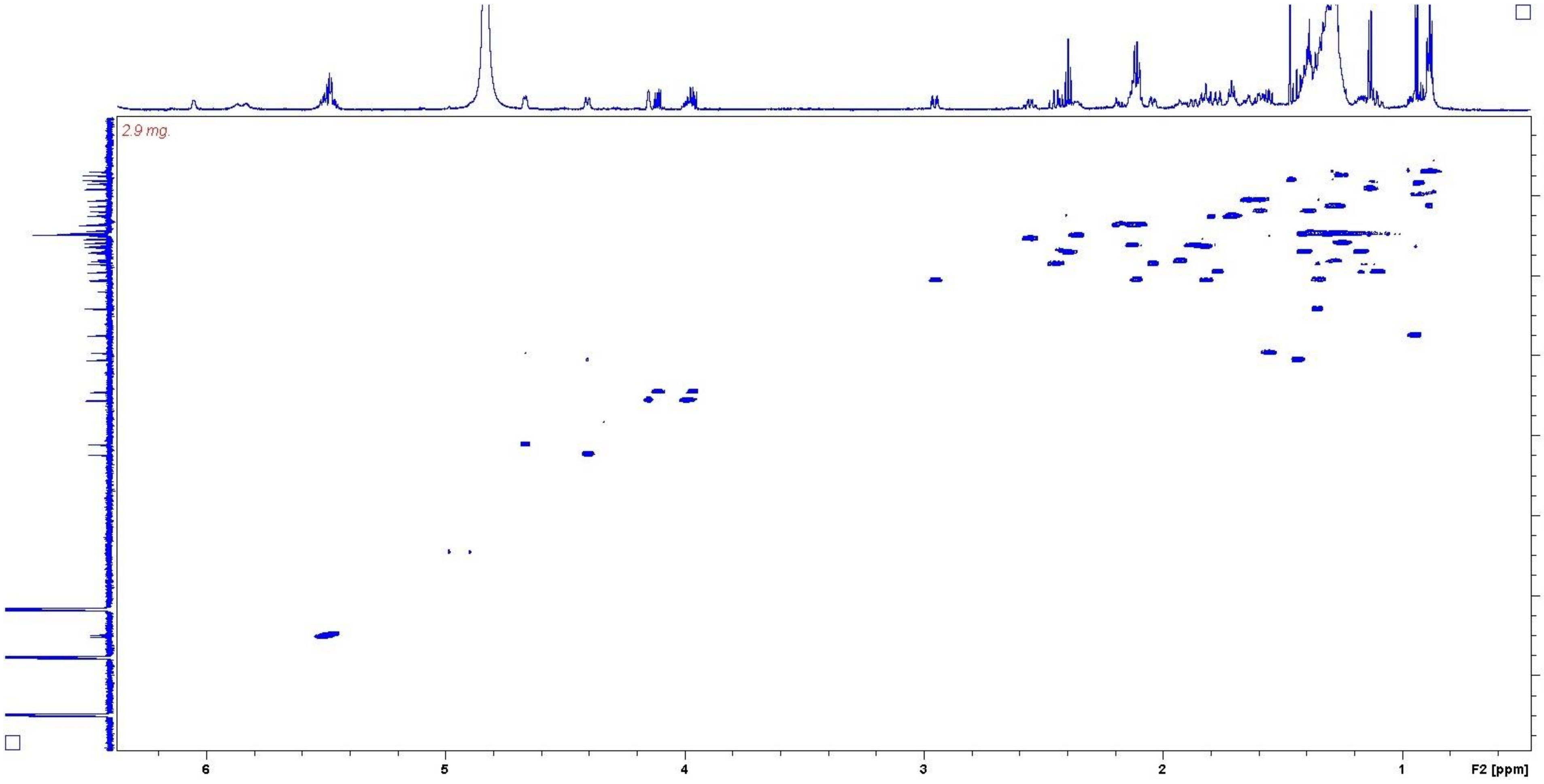

Figure S46. HMBC spectrum of compound **4** in C_5D_5N.

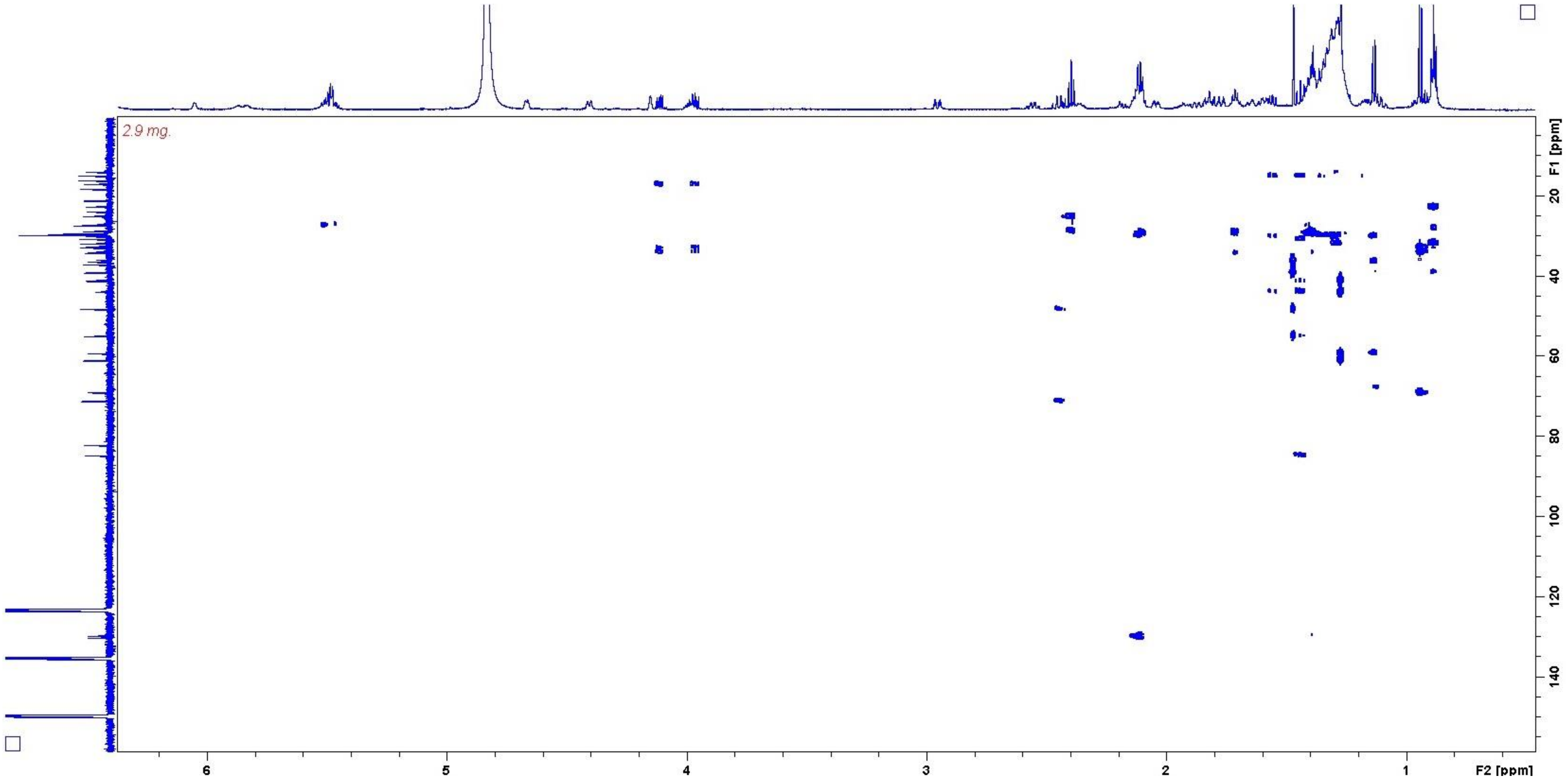

Figure S47. ROESY spectrum of compound **4** in C_5D_5N.

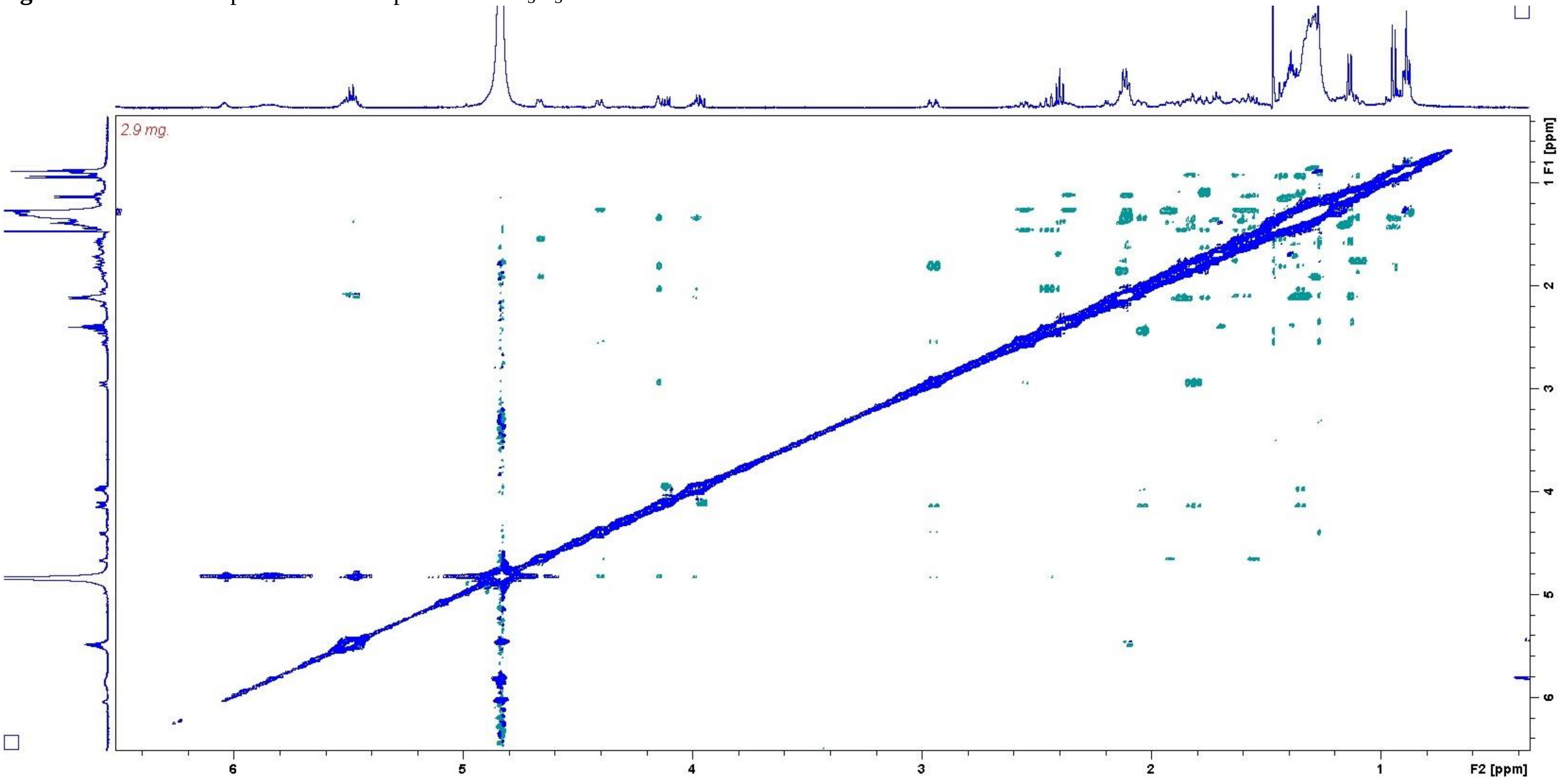

Figure S48. Cytotoxicity compounds **1–4** after 24 h.

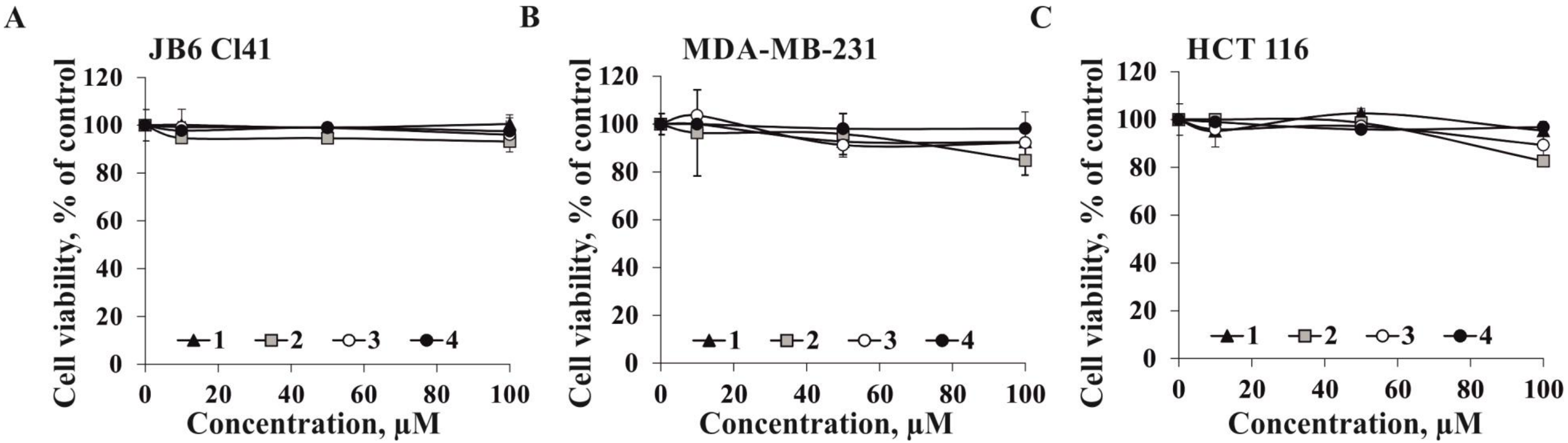

Supplement: Supplementary file 1 [file marinedrugs-18-00260-s001.pdf]
